# Copper-mediated 1,2-bis(trifluoromethylation) of arynes†
†Electronic supplementary information (ESI) available. See DOI: 10.1039/c8sc03754j


**DOI:** 10.1039/c8sc03754j

**Published:** 2018-09-21

**Authors:** Xinkan Yang, Gavin Chit Tsui

**Affiliations:** a Department of Chemistry , The Chinese University of Hong Kong , Shatin , New Territories , Hong Kong SAR . Email: gctsui@cuhk.edu.hk

## Abstract

We herein describe an unprecedented 1,2-bis(trifluoromethylation) of arynes with [CuCF_3_] in the presence of an oxidant DDQ. The method allows the rapid construction of a new class of 1,2-bis(trifluoromethyl)arenes in one-step from aryne precursors under mild conditions. Its synthetic utility has been demonstrated in the preparation of bis(trifluoromethylated) molecules with potential pharmaceutical and materials science applications. Mechanistic studies indicated the presence of an *o*-trifluoromethyl aryl radical intermediate *via* CF_3_ group transfer from [CuCF_3_] to the aryne.

## Abstract 2

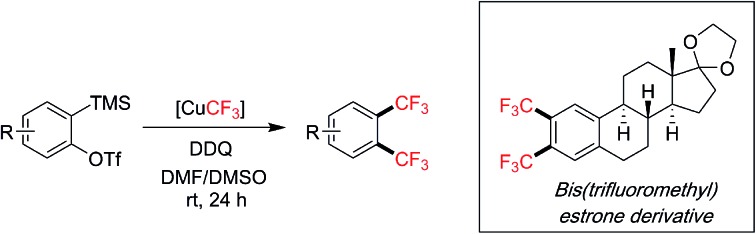
A novel 1,2-bis(trifluoromethylation) of arynes using [CuCF_3_] is described.

## Introduction

**Scheme 1:**
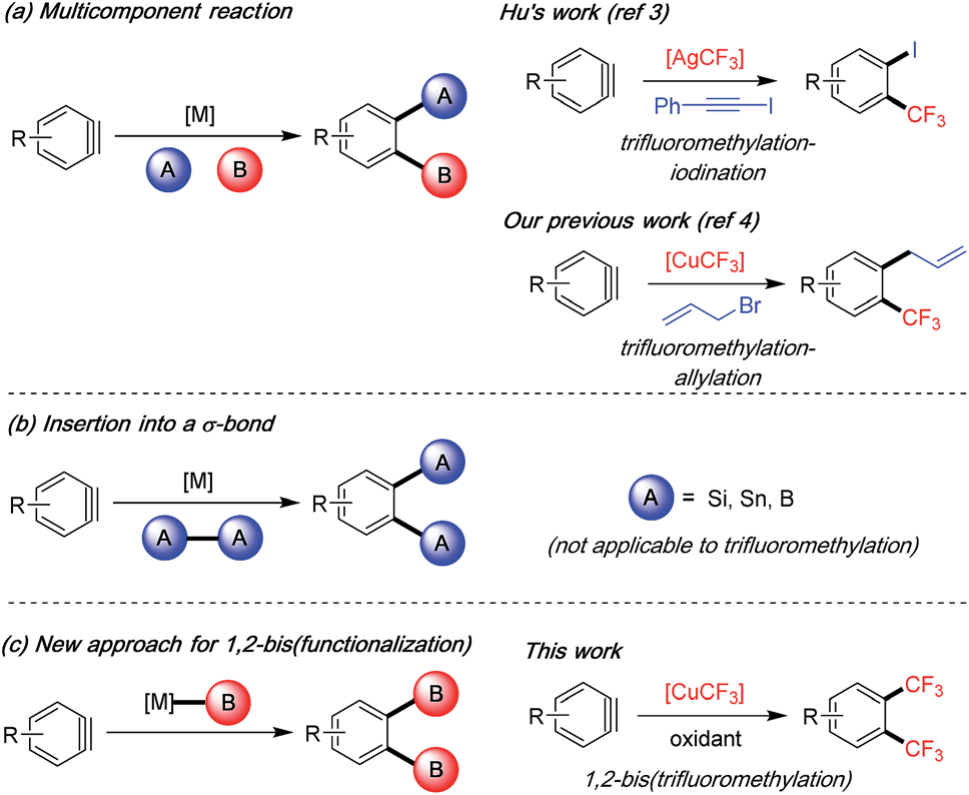
Transition metal-catalyzed/-mediated 1,2-difunctionalization of arynes and applications in trifluoromethylation reactions.

Arynes are versatile reactive intermediates for the rapid synthesis of multifunctionalized arenes.^1^ In particular, multicomponent reactions of arynes in the presence of a transition metal can provide easy access to diverse 1,2-difunctionalized arenes.1a,b The two new bonds are formed in one step on the aryne intermediate to install orthogonal functional groups adjacent to each other (Scheme 1a). This strategy has only been recently applied by Hu's group and us to the synthesis of highly functionalized trifluoromethylated arenes, which are important building blocks in widely used pharmaceuticals and agrochemicals.^2^ Hu and co-workers developed a silver-mediated trifluoromethylation–iodination^3^ of arynes whereas we reported a copper-mediated trifluoromethylation–allylation protocol;^4^ both methods successfully realized vicinal difunctionalization of arenes involving C–CF_3_ bond and C–I/C–C bond construction in one-step from aryne intermediates. On the other hand, to install two identical vicinal functional groups onto arenes, metal-catalyzed aryne insertion to a σ-bond has been utilized. Under Pd, Cu or Pt catalysis, arynes/hetarynes can insert into heteroatom–heteroatom bonds such as Si–Si, Sn–Sn and B–B bonds to provide 1,2-bis(functionalized) arenes with high synthetic utility (Scheme 1b).^5^ However, such a method is not amenable to the preparation of bis-CF_3_ products due to the fact that an infeasible aryne insertion to a “CF_3_–CF_3_” bond would be required.^6^ We herein describe a new approach for 1,2-bis(functionalization) of arenes by reacting the aryne intermediate with an organometallic reagent twice, in this case [CuCF_3_], thereby achieving an unpresented 1,2-bis(trifluoromethylation) of arynes (Scheme 1c).

## Results and discussion

During our investigation of the trifluoromethylation–allylation of aryne precursor **1a** using the fluoroform-derived [CuCF_3_],^4^ we observed that the reaction produced an unexpected 1,2-bis(trifluoromethyl)arene product **2a** (30% yield) when open to air without the electrophile allylbromide (Table 1, entry 1). The [CuCF_3_] reagent was prepared from CuCl, *t*-BuOK and CF_3_H as a solution in DMF according to Grushin's procedure,^7^,^8^ and stabilized with Et_3_N·3HF. The oxidative condition was crucial for the formation of **2a** (Table 1, entry 2). Screening of various oxidants revealed that DDQ (2,3-dichloro-5,6-dicyano-1,4-benzoquinone) was capable of increasing the yield (58%) (Table 1, entries 3–7). A major side product was the mono-trifluoromethylated arene (regioisomeric mixture); its formation could be reduced by using DMSO as a co-solvent (1 : 1 ratio), thus further improving the yield (77%) (Table 1, entry 8). Reaction at room temperature was equally effective (Table 1, entry 9). Different DMF/DMSO ratios were tested showing that a larger amount of DMSO was generally beneficial for the reaction, and at 1 : 2 ratio (DMF/DMSO) the mono-CF_3_ side products could be completely suppressed (Table 1, entries 10 and 11). Other reaction parameters such as additives, co-solvents and reagent equivalents were screened with no further improvement.^8^ The reaction design requires at least two equivalents of [CuCF_3_] due to its role as a “carrier of CF_3_”; we found that four equivalents were necessary to provide the highest yield. However, this stable reagent can be prepared from inexpensive copper and CF_3_ sources at scale7*d* (fluoroform is an industrial byproduct and commercially available at <$0.10/mol),^9^ therefore justifying its use in excess.

**Table 1:** Optimization studies for 1,2-bis(trifluoromethylation) of aryne precursor **1a**^*a*^

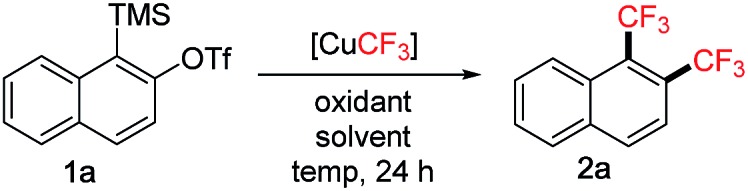				
Entry	Oxidant	Solvent	Temp. (°C)	Yield^*b*^ (%)
1^*c*^	Air	DMF	50	30
2	None	DMF	50	0
3	BQ	DMF	50	4
4	Cu(OAc)_2_	DMF	50	7
5	AgOAc	DMF	50	26
6	PhI(OAc)_2_	DMF	50	26
7	DDQ	DMF	50	58
8^*d*^	DDQ	DMF/DMSO	50	77
9^*d*^	DDQ	DMF/DMSO	rt	78
10^*e*^	DDQ	DMF/DMSO	rt	62
11^*f*^	DDQ	DMF/DMSO	rt	77

^*a*^Unless specified otherwise, reactions were carried out using **1a** (0.1 mmol), [CuCF_3_] (0.4 mmol in 1.0 mL DMF), oxidant (0.2 mmol) and DMF (1.0 mL), under argon.

^*b*^Determined by ^19^F NMR analysis using benzotrifluoride as the internal standard.

^*c*^Reaction was open to air.

^*d*^DMF : DMSO = 1.0 : 1.0 mL.

^*e*^DMF : DMSO = 1.0 : 0.5 mL.

^*f*^DMF : DMSO = 1.0 : 2.0 mL.

**Scheme 2:**
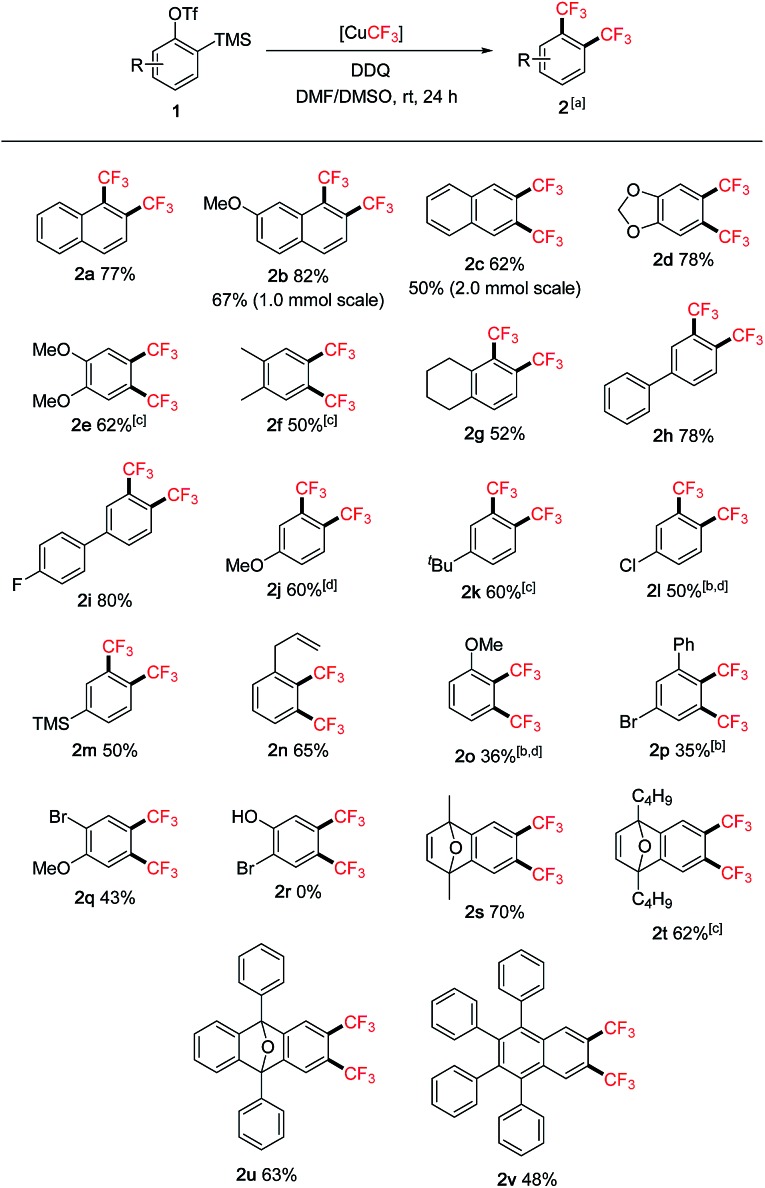
Scope of 1,2-bis(trifluoromethyl)arenes **2**. ^a^General conditions: **1** (0.4 mmol), DDQ (0.8 mmol), [CuCF_3_] (1.6 mmol in 4.0 mL DMF), DMSO (8.0 mL), under argon. Isolated yields. ^b^DMF : DMSO = 4.0 : 4.0 mL. ^c^DMF : DMSO = 4.0 : 12.0 mL. ^d^Yield determined by ^19^F NMR analysis using benzotrifluoride as the internal standard.

**Scheme 3:**
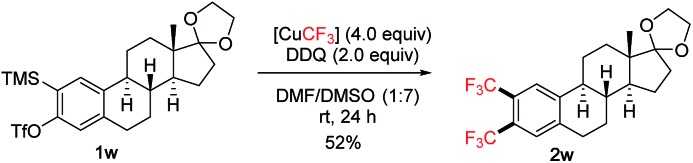
1,2-Bis(trifluoromethylation) of an estrone derivative.

The scope of the reaction was subsequently investigated using various 2-(trimethylsilyl)aryltriflates **1** as the aryne precursors (Scheme 2). Although many methods are available for generating aryne intermediates,1*f* the 2-(trimethylsilyl)aryl triflates **1**, developed by Kobayashi and co-workers in 1983,^10^ remain the most convenient and widely used precursors owing to the mild conditions (usually when exposed to fluoride) and broad synthetic applications.1a,bThey are either commercially available or can be prepared in a few steps at scale according to known procedures. In our reaction, conveniently no extra fluoride was needed to generate arynes from **1** due to the addition of Et_3_N·3HF as a stabilizer to the [CuCF_3_] reagent (*in situ* generating KF with *t*-BuOK).^7^ Moderate to good yields were obtained for symmetrical and unsymmetrical 1,2-bis(trifluoromethyl)arenes (**2a–g**). Functional groups such as acetal (**2d**), chloro (**2l**), bromo (**2p**, **2q**), allyl (**2n**) and even silyl (**2m**) were tolerated. Hydroxy group (**2r**), on the other hand, was not compatible. Substituents adjacent to the reaction centre (**2o**, **2p**) generally caused lower yields than remote substituents (**2j**, **2h**). Oxabicyclic compounds containing the bis-CF_3_ moiety were also synthesized in reasonable yields (**2s–u**), they could serve as useful substrates for asymmetric ring-opening reactions^11^ towards drug analogue preparation. The bis-CF_3_ polyaromatic compound (**2v**) was also synthesized which may exhibit interesting material properties; the lower yield was mainly due to the solubility issue of the precursor. In some cases, larger amounts of DMSO were required to inhibit the formation of mono-CF_3_ side products (**2e**, **2f**, **2k**, **2t**). Several products were very volatile, and their NMR yields were determined (**2j**, **2l**, **2o**). Pharmaceutical compounds containing two trifluoromethyl groups on arenes are well-precedented,^12^ however, the class of 1,2-bis(trifluoromethyl)arenes has been much less represented possibly due to the difficulty in their synthesis. Traditional methods often involved hazardous conditions (using SF_4_ and HF) and lengthy procedures from trifluoromethylated building blocks.^13^ Our approach is more operationally simple and general for synthesizing structurally diverse 1,2-bis(trifluoromethyl)arenes. Furthermore, aryne precursor **1w** derived from estrone^6^ was successfully converted into the 1,2-bis(trifluoromethylated) derivative **2w** (Scheme 3) demonstrating relevance of the current method to the modification of biologically active compounds.

**Scheme 4:**
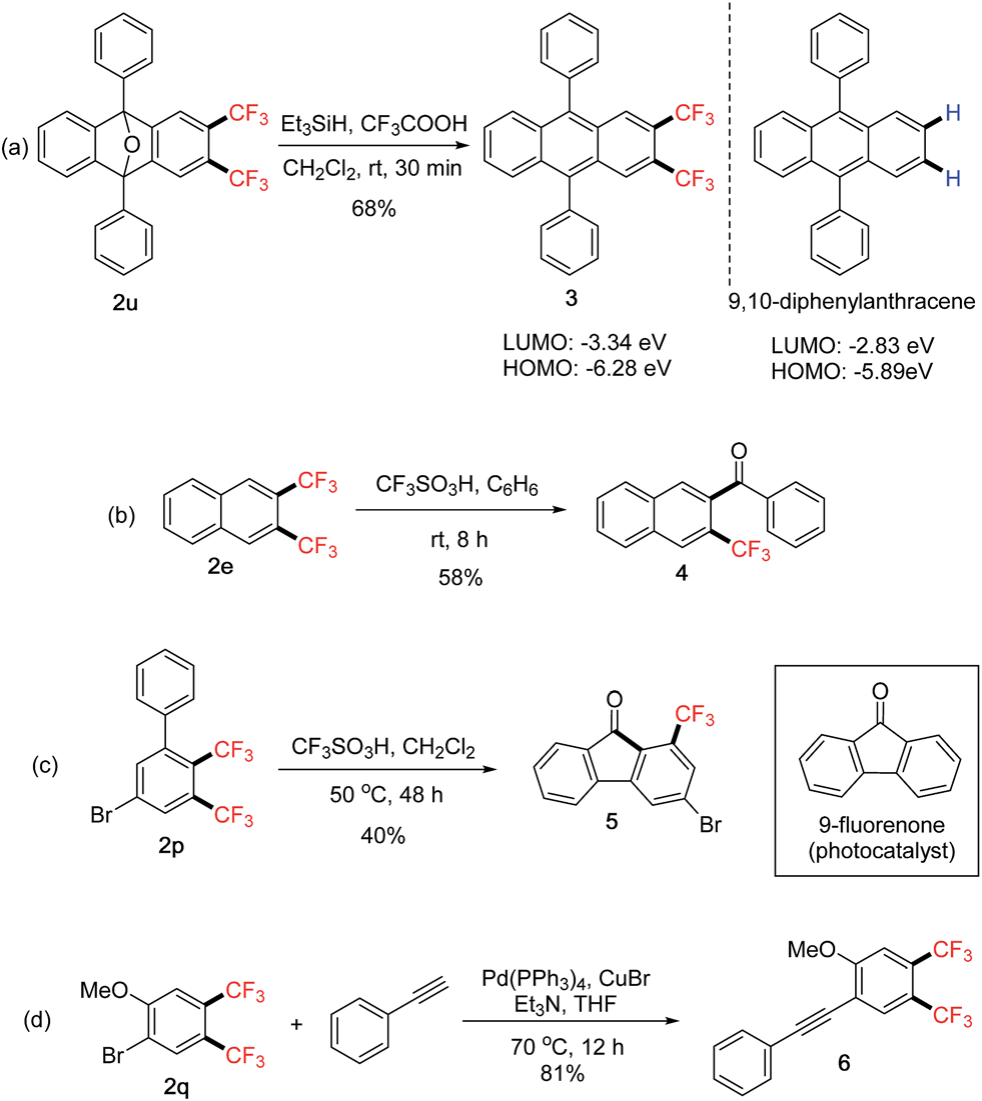
Further transformations of 1,2-bis(trifluoromethyl)arenes **2**.

The above 1,2-bis(trifluoromethyl)arenes **2** are useful intermediates for further transformations and their synthetic applications were explored (Scheme 4). Deoxygenation of compound **2u**^14^ directly led to the bis(trifluoromethylated) 9,10-diphenylanthracene derivative **3**. UV-Vis absorption and cyclic voltammetry (CV) studies showed decreased HOMO and LUMO energy levels compared with the parent compound (Scheme 4a).^8^ This “tuning” effect by the bis-CF_3_ groups could have potential applications in the development of organic semiconductors.^15^ Under protolytic defluorination protocols,^16^ trifluoromethylated ketone **4** could be obtained from **2e** (Scheme 4b). The intramolecular reaction afforded compound **5** from **2p** (Scheme 4c), which is a trifluoromethylated analogue of 9-fluorenone that has been recently shown as an effective metal-free photocatalyst.^17^ Finally, Sonogashira cross-coupling of **2q** with a terminal alkyne afforded compound **6**, which could provide access to bis(trifluoromethylated)benzofurans *via* cyclization of the –OMe group.^18^

**Scheme 5:**
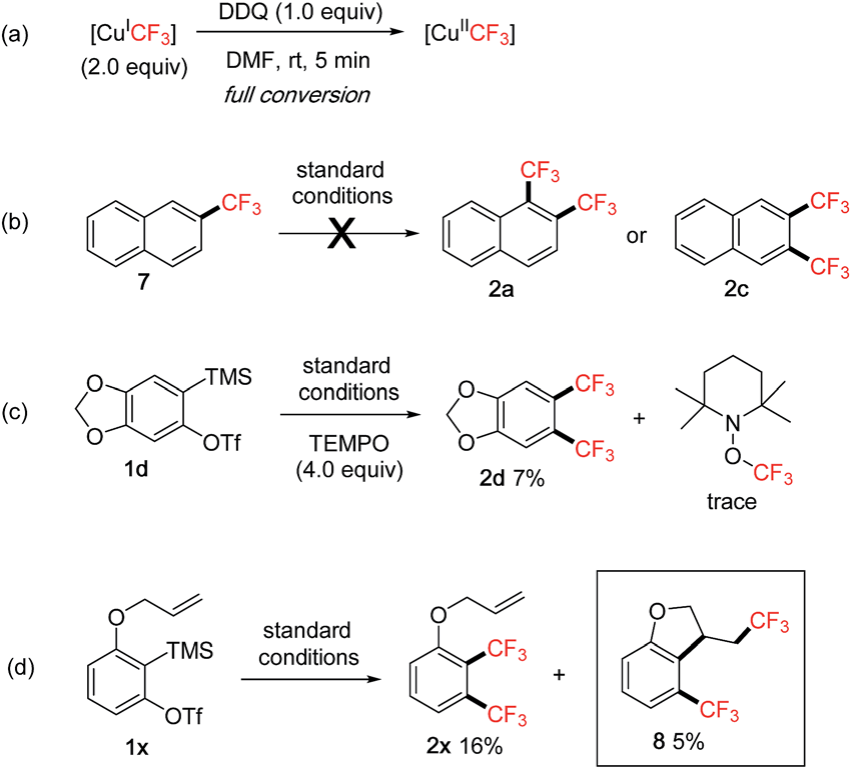
Mechanistic studies.

To gain more insights into the reaction mechanism, additional studies were performed (Scheme 5).^8^ In the ^19^F NMR experiment, we observed that the peak of the initial [Cu^I^CF_3_]7*a* quickly disappeared after adding DDQ and stirring for 5 min at rt, indicating a facile oxidation of [Cu^I^CF_3_] presumably to [Cu^II^CF_3_]7*c* by DDQ (Scheme 5a). Subjecting mono-CF_3_ compound **7** to the standard conditions did not give any bis-CF_3_ products **2a** or **2c**, thus ruling out the C–H trifluoromethylation pathway (Scheme 5b).^19^ Adding a known radical scavenger TEMPO (2,2,6,6-tetramethyl-1-piperidinyloxy)20*a* to the reaction of **1d** under standard conditions dramatically decreased the yield of **2d** (Scheme 5c, 7% *vs.*78% *cf.*Scheme 2). Significant amounts of the mono-CF_3_ product (29%) and the CF_3_-containing dimer product (12%) were detected, however, only trace TEMPO–CF_3_ adduct was observed. Also, styrene derivatives20b,c were added to the standard conditions with **1d** to trap any CF_3_-adducts, but only trace amounts were detected. These studies suggested that CF_3_ radicals were not likely to be present in the reaction. On the other hand, a radical clock experiment20*d* using substrate **1x** gave both bis-CF_3_ product **2x** and cyclized product **8**, therefore hinting at the intermediacy of a transient aryl radical (Scheme 5d).

**Scheme 6:**
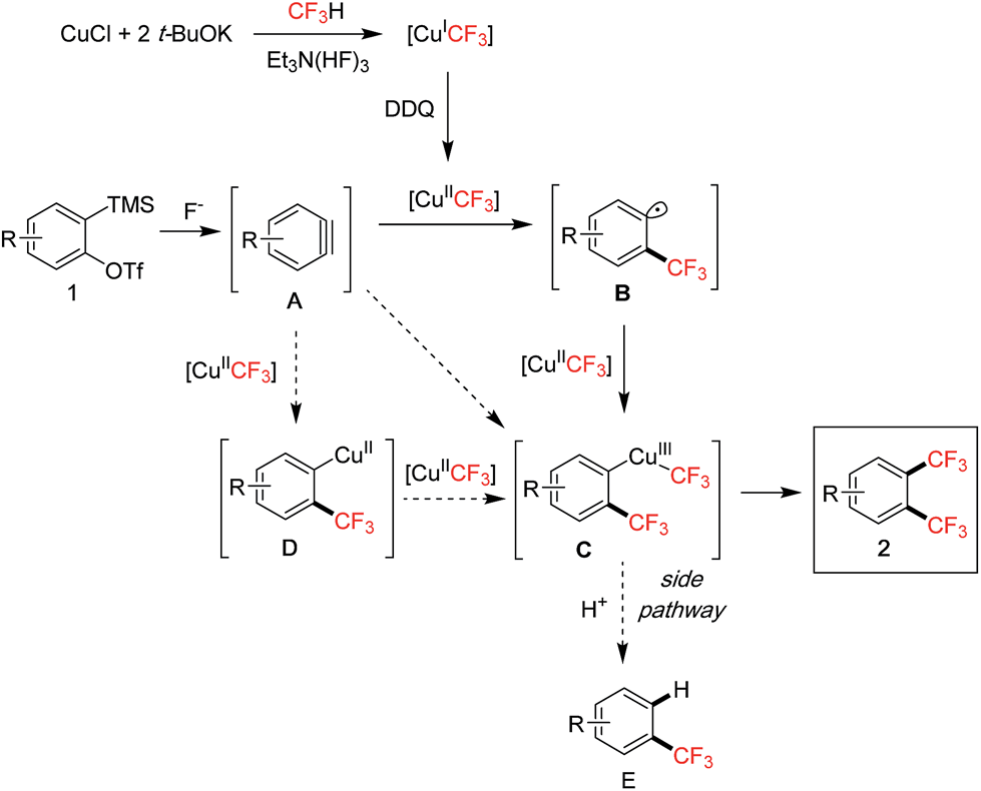
Proposed mechanism.

Based on the above studies and literature examples, we propose the following mechanism for the 1,2-bis(trifluoromethylation) of arynes (Scheme 6). The initial fluoroform-derived [Cu^I^CF_3_] is quickly oxidized by DDQ to [Cu^II^CF_3_].7*c* The [Cu^II^CF_3_] is capable of transferring a CF_3_ group to aryne **A** resulting in an aryl radical species **B**,^21^ supported by the radical clock experiment (*cf.*Scheme 5d) and our own observation of CF_3_ group transfer to alkenes with [Cu^II^CF_3_] for generating alkyl radicals.^22^ Intermediate **B** reacts with a second equivalent of [Cu^II^CF_3_] presumably leading to a Cu^III^–CF_3_ species **C**.20*b* Final reductive elimination affords the 1,2-bis(trifluoromethyl)arene product **2**. Related reactions of aryl radicals and [CuCF_3_] to form aryl–CF_3_ bonds have been reported.^23^ There also exists the possibility that intermediate **C** may arise *via* carbocupration^24^ processes with [CuCF_3_] under oxidative conditions. For instance, aryne **A** may undergo carbocupration with a [Cu(CF_3_)_*n*_]^25^ species to give **C**. Alternatively, an arylcopper intermediate **D** may be formed first, which then reacts with another molecule of [Cu^II^CF_3_] leading to **C**. It is difficult to pinpoint the exact pathway at the moment due to the complicated nature of the fluoroform-derived [CuCF_3_] reagent, especially in oxidized forms. A major side reaction was the formation of the mono-CF_3_ product **E ***via* protodemetallation of **C** (*t*-BuOH is present in the reagent and can act as a proton source). This pathway is inhibited by adding DMSO as a co-solvent, probably due to its role as a coordinating ligand to stabilize the copper complex **C** thus favouring reductive elimination.^26^

## Conclusions

In conclusion, a novel 1,2-bis(trifluoromethylation) of arynes using [CuCF_3_] has been developed. By employing 2-(trimethylsilyl)aryl triflates as aryne precursors, structurally diverse 1,2-bis(trifluoromethyl)arenes can be synthesized in one-step under mild and safe conditions. Notably the ultimate source of CF_3_ in all of these valuable compounds is the inexpensive industrial waste fluoroform. New mechanistic insights will further the field of copper-mediated/-catalyzed trifluoromethylation–functionalization of arynes, and related studies are currently ongoing in our laboratory.

## Conflicts of interest

There are no conflicts to declare.

## Supplementary Material

Supplementary information
