# Supplementary material for: Copper-mediated 1,2-bis(trifluoromethylation) of arynes
Source: Chem Sci. 2018 Sep 21;9(47):8871–5. doi: 10.1039/c8sc03754j (PMC6296300; doi:10.1039/c8sc03754j)
Supplement: Supplementary file 1 [file SC-009-C8SC03754J-s001.pdf]

Electronic Supplementary Material (ESI) for Chemical Science.
This journal is © The Royal Society of Chemistry 2018

Copper-Mediated 1,2-Bis(trifluoromethylation) of Arynes

Xinkan Yang and Gavin Chit Tsui*

Table of Contents

General Experimental S1
Materials S1
Instrumentation S1
Experimental Procedures S2
Mechanistic studies S11
Table S1 Optimization studies. S14
Characterization Data S16
Spectra S23

General Experimental. Unless otherwise noted, reactions were carried out under argon in a 25mL round-bottom flask with magnetic stirring. Analytical thin layer chromatography (TLC) was performed with EM Science silica gel 60 F254 aluminum plates. Visualization was done under a UV lamp (254 nm) and by immersion in ethanolic phosphomolybdic acid (PMA) or potassium permanganate ($KMnO_4$), followed by heating using a heat gun. Organic solutions were concentrated by rotary evaporation at 23–35 °C. Purification of reaction products were generally done by flash column chromatography with Grace Materials Technologies 230–400 mesh silica gel.

Materials. Fluoroform (Research Grade, Purity: 99.999% min., 9.1kg in 16 L size cylinder) was purchased from SynQuest Laboratories, USA. Copper(I) chloride (extra pure, 99.99%) was purchased from Acros. $Et_3N{\cdot}3HF$ (97%) and anhydrous DMSO was purchased from J&K Scientific. Potassium *tert*-butoxide (97%) was purchased from Alfa Aesar. DMF was dried over Solvent Purification System then bubbled with argon for 24 h. Other chemicals for substrates preparation were purchased from Acros, J&K Scientific, Aldrich and Dikemann.

Instrumentation. Proton nuclear magnetic resonance spectra (^{1}H NMR) spectra, carbon nuclear magnetic resonance spectra (^{13}C NMR) and fluorine nuclear magnetic resonance spectra (^{19}F NMR) were recorded at 23 °C on a Bruker 400 spectrometer in $CDCl_3$ (400 MHz for ^{1}H, 101 MHz for ^{13}C and 376 MHz for ^{19}F) and Bruker 500 spectrometer in $CDCl_3$ (500 MHz for ^{1}H, 126 MHz for ^{13}C and 470 MHz for ^{19}F). Chemical shifts for protons were reported as parts per million in δ scale using solvent residual peak ($CHCl_3$: 7.26 ppm) or tetramethylsilane (0.00 ppm) as internal standards. Chemical shifts of ^{13}C NMR spectra were reported in ppm from the central peak of $CDCl_3$ (77.16 ppm) on the δ scale. Chemical shifts of ^{19}F NMR are reported as parts per million in δ scale using benzotrifluoride (-63.72 ppm) as internal standards. Data are represented as follows: chemical shift, integration, multiplicity (s = singlet, d = doublet, t = triplet, q = quartet, qn = quintuplet, sx = sextet, sp = septuplet, m = multiplet, br = broad), and coupling constant (*J*, Hz). High resolution mass spectra (HRMS) were obtained on a Finnigan MAT 95XL GC Mass Spectrometer or a Thermo Scientific Q Exactive Focus Mass Spectrometer or Bruker 9.4T FTICR Mass Spectrometer. The control experiment results were obtained on a Shimadzu GCMS-QP2010 SE GC MS Spectrometer.

Experimental Procedures.

Preparation of fluoroform-derived [$CuCF_3$] reagent:[1]

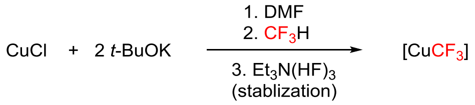

In a glove box, to a glass tube was charged CuCl (400 mg, 4.0 mmol), *t*-BuOK (944 mg, 8.0 mmol) and a stirrer bar. The tube was sealed with a septum, brought out of the glove box and put under an argon atmosphere. Degassed DMF (8.0 mL) was added *via* syringe and the mixture was stirred at room temperature for 30 min. Then fluoroform was bubbled into the mixture by using a needle connected to the fluoroform cylinder at room temperature for 3 min. After removing the fluoroform inlet, the mixture was stirred for 5 min and $Et_3N(HF)_3$ (212 µL, 1.32 mmol) was slowly added under argon and the mixture was stirred for another 5 min. A slightly brown solution with some white solid was obtained as the [$CuCF_3$] solution in DMF (~0.40 M).

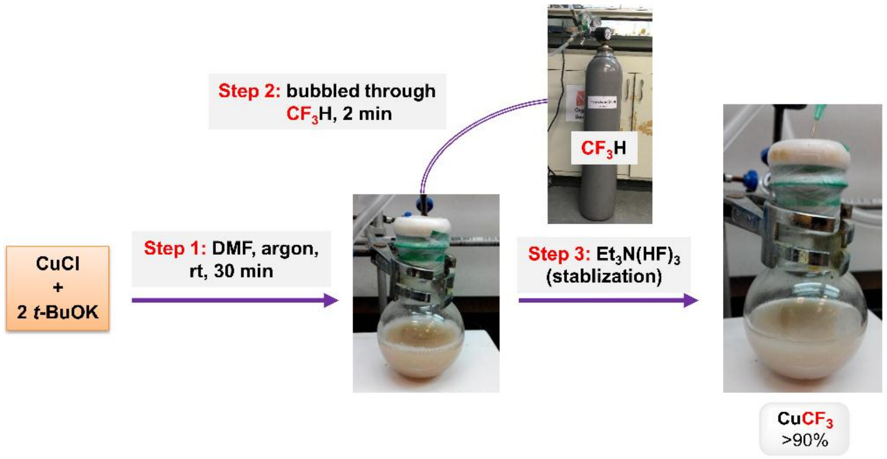

General procedure for 1,2-bis(trifluoromethylation) of arynes:

Under argon, to a 25 mL round-bottom flask equipped with a magnetic stir bar was added benzyne precursor **1** (0.4 mmol), DDQ (0.8 mmol) and DMSO. A solution of [$CuCF_3$] in DMF (4.0 mL, 1.6 mmol) was added dropwise to the above mixture under argon at 0 °C. The reaction mixture was warmed up to room temperature and stirred under argon for 24 h, then quenched with sat. aq. $NaHCO_3$ solution, neutralized with 1 M HCl, and extracted with diethyl ether for three times. The organic layers were combined, washed with water then brine, dried over anhydrous Na_2SO_4, filtered and concentrated by rotary evaporator. The crude product was purified by flash column chromatography on silica gel.

(1) (a) Zanardi, A.; Novikov, M. A.; Martin, E.; Benet-Buchholz, J.; Grushin, V. V. *J. Am. Chem. Soc.* **2011**, *133*, 20901. (b) Yang, X.; Tsui, G. C. *Org. Lett.* **2018**, *20*, 1179.

Aryne precursors:

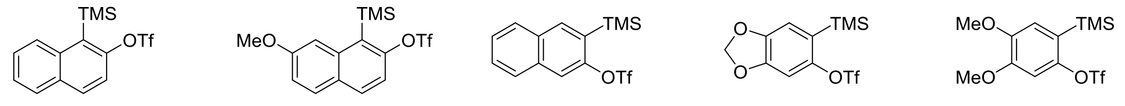

1a **1b** **1c** **1d** **1e**

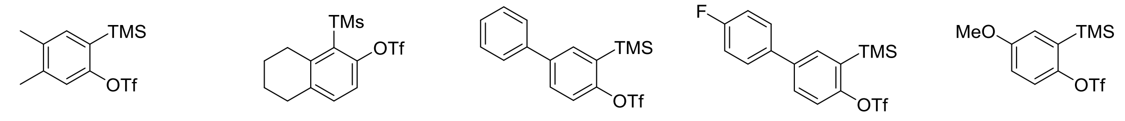

1f **1g** **1h** **1i** **1j**

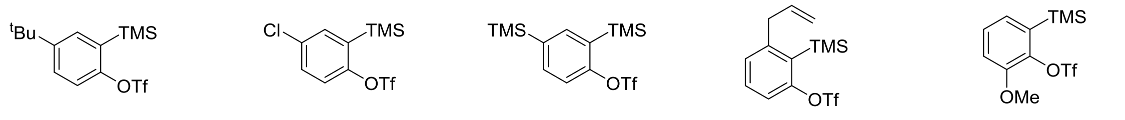

1k **1l** **1m** **1n** **1o**

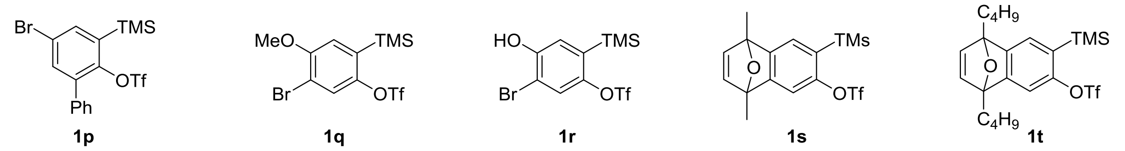

1p **1q** **1r** **1s** **1t**

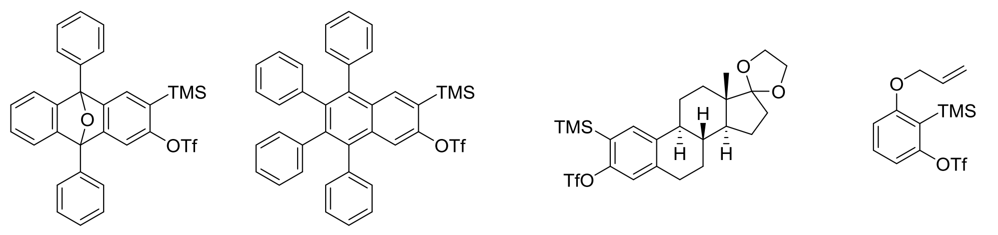

1u **1v** **1w** **1x**

The aryne precursors **1a-1h, 1l-1p, 1r, 1v,** were synthesized according to the literature procedures[2] from the corresponding 2-bromophenol. **1q** was synthesized from **1r**, **1w** was prepared from estrone through five steps according to literature reported procedure[2q].

(2) (a) Medina, M. J.; Mackey, J. L.; Garg, N. K.; Houk, K. N. *J. Am. Chem. Soc.* **2014**, *136*, 15798. (b) Dubrovskiy, A. V.; Larock. R. C. *Org. Lett.* **2010**, *12*, 1180. (c) Łączkowski, K. Z.; Garcia, D.; Peña, D.; Cobas, A.; Pérez, D.; Guitián, E. *Org. Lett.* **2011**, *13*, 960. (d) Yoshida, H.; Yoshida, R.; Takaki, K. *Angew. Chem. Int. Ed.* **2013**, *52*, 8629. (e) Zeng, Y.; Hu, J. *Org. Lett.* **2016**, *18*, 856. (f) Zeng, Y.; Zhang, L.; Zhao, Y.; Ni, C.; Zhao, J.; Hu, J. *J. Am. Chem. Soc.* **2013**, *135*, 2955. (g) Chen, Q.; Yan, X.-X.; Du, Z.-Y.; Zhang, K.; Wen, C.-X. *J. Org. Chem.* **2016**, *81*, 276. (h) Hiroto, Y.; Junnai, I.; Miwa, S.; Joji, O.; Kunai, A. *J. Am. Chem. Soc.* **2003**, *125*, 6638. (i) Yoshida, H.; Sugiura, S.; Kunai, A. *Org. Lett.* **2002**, *4*, 2767. (j) Mesgar, M.; Daugulis, O. *Org. Lett.* **2017**, *19*, 4247. (k) Sakai, H.; Kubota, T.; Yuasa, J.; Araki, Y.; Sakanoue, T.; Takenobu, T.; Wada, T.; Kawai, T.; Hasobe, T. *J. Phys. Chem. C* **2016**, *120*, 7860. (l) Ikawa, T.; Masuda, S.; Takagi, A.; Akai, S. *Chem. Sci.* **2016**, *7*, 5206. (m) Alonso, J. M.; Díaz-Álvarez, A. E.; Criado, A.; Perez, D.; Peña, D.; Guitián, E. *Angew. Chem., Int. Ed.* **2012**, *51*, 173. (n) Rodríguez-Lojo, D.; Peña, D.; Pérez, D.; Guitián, E. *Synlett* **2015**, *26*, 1633–1637. (o) Moreira, B. V.; Muraca, A. C. A.; Raminelli, C. *Synthesis* **2017**, *49*, 1093. (p) Hendrick, C. E.; Wang, Q. *J. Org. Chem.* **2015**, *80*, 1059. (q) Mesgar, M.; Daugulis, O. *Org. Lett.* **2017**, *19*, 4247.

Preparation of 4'-fluoro-3-(trimethylsilyl)-[1,1'-biphenyl]-4-yl trifluoromethanesulfonate (1i):

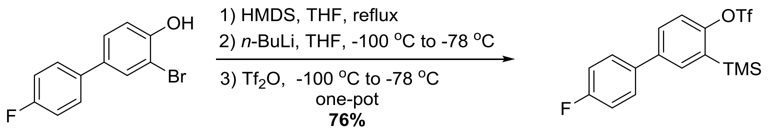

S1 **1i**

A mixture of ortho-bromohydroxyarene **S1** (1.16 g, 4.36 mmol) and HMDS (1.26 ml, 6.10 mmol) in THF (4 ml) was refluxed for 3h. The solvent was evaporated under reduced pressure and the residue was subjected to vacuum to remove excess NH_3 and unreacted HMDS. After 1H NMR confirmation of the quantitative formation of the corresponding silyl ether, the crude product was dissolved in THF (30 ml), the solution was cooled to -100 °C and n-BuLi (1.6 M in hexane, 3.0 ml, 4.8 mmol) was added drop wise. The mixture was stirred for 20 min while the temperature reached to -78 °C. Then the mixture was again cooled to -100 °C, Tf_2O (0.88 ml, 5.23 mmol) was added drop wise and stirring was continued for 20 min while the temperature reached to -78 °C. Cold sat. aq. $NaHCO_3$ was added, the phases were separated and the aqueous layer was extracted with Et_2O. The combined organic layers were dried with Na_2SO_4. Filtered and concentrated under reduced pressure. Purification of the residue by column chromatography afforded silyl triflate **1i** (1.28 g, 3.27 mmol, 76% yield). R_f = 0.8 (hexane : EtOAc = 8:1). **^{1}H NMR** (500 MHz, $CDCl_3$): δ 7.67 (s, 1H), 7.58 (d, J = 8.5 Hz, 1H), 7.54-7.51 (m, 2H), 7.52 (d, J = 8.5 Hz, 1H), 7.16 (t, J = 8.5 Hz, 2H), 0.44 (s, 9H) ppm. **^{13}C NMR** (126 MHz, $CDCl_3$): δ 162.9 (d, J_{C-F} = 249.9 Hz), 154.6, 139.8, 136.0 (d, J_{C-F} = 3.3 Hz), 134.9, 133.3, 129.9, 129.0 (d, J_{C-F} = 8.1 Hz), 120.0, 118.7 (q, J_{C-F} = 320.7 Hz), 116.0 (d, J_{C-F} = 21.5 Hz), -0.686 ppm. **^{19}F NMR** (470 MHz, $CDCl_3$): δ -74.93 (s, 3F), -115.6 (m, 1F) ppm. **HRMS m/z (APCI)**: calcd. for $C_{16}H_{16}F_4O_3SSi$ $[M]^+$: 392.0520; found: 392.0524.

Preparation of 5-bromo-4-methoxy-2-(trimethylsilyl)phenyl trifluoromethanesulfonate (1q):

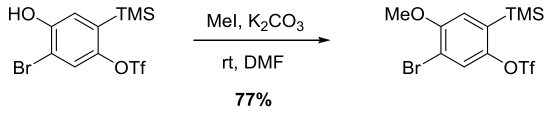

1r **1q**

Under argon, to a 10 mL round-bottom flask equipped with a magnetic stir bar was added **1r** (412 mg, 1.05 mmol), iodomethane (231 mg, 1.63 mmol) and DMF (2.1 mL), then K_2CO_3 (225 mg, 1.63mmol) was added in one portion and the reaction system was stirred at room temperature for 3 h, then quenched by adding water, extracted with ether for three times. The organic layers were combined, washed with water then brine, dried over anhydrous Na_2SO_4. Filtered and concentrated by rotary evaporator. The crude product was purified by flash column chromatography on silica gel to afford **1q** (330 mg, 0.81 mmol, 77% yield). R_f= 0.6 (hexane : EtOAc = 8:1). **^{1}H NMR** (400 MHz, $CDCl_3$): δ 7.50 (s, 1H), 6.95 (m, 2H), 3.92 (s, 3H), 0.37 (s, 9H) ppm. **^{13}C NMR** (101 MHz, $CDCl_3$): δ 155.1, 147.6, 133.5, 125.1, 118.6 (q, J_{C-F} = 321.3 Hz), 117.4, 113.4, 56.7, -0.772 ppm. **^{19}F NMR** (376 MHz, $CDCl_3$): δ -74.89 (s, 3F) ppm. **HRMS m/z (APCI)**: calcd. for $C_{11}H_{14}BrF_3O_4SSi$ $[M]^+$: 407.9492; found: 407.9493.

Preparation of 1,4-dibutyl-7-(trimethylsilyl)-1,4-dihydro-1,4-epoxynaphthalen-6-yl trifluoromethanesulfonate (1t):

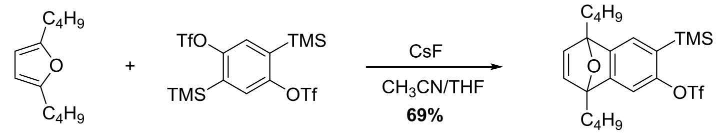

S3 **S2** **1t**

Under argon, CsF (456 mg, 3.0 mmol) was added to a solution of **S3** (504 mg, 1.4 mmol) and **S2** (1.04 g, 2.0 mmol) in dry CH_3CN (20 mL) and THF (20 mL), the mixture was stirred at room temperature, monitored by TLC. After completion, the system was diluted with EA, passed through a short pad, evaporated the solvent and the residue was purified by flash column chromatography on silica gel to afford **1t** (570 mg, 1.2 mmol, 60% yield), R_f = 0.2 (hexane : CH_2Cl_2 = 8:1). **^{1}H NMR** (500 MHz, $CDCl_3$): δ 7.15 (s, 1H), 7.06 (m, 1H), 6.78 (q, J = 5.8 Hz, 2H), 2.54-2.24 (m, 2H), 2.22-2.15 (m, 2H), 1.66-1.52 (m, 4H), 1.52-1.43 (m, 4H), 0.98 (q, J = 6.8 Hz, 6H), 0.34 (s, 9H) ppm. **^{13}C NMR** (126 MHz, $CDCl_3$): δ 157.7, 152.5, 151.8, 146.1, 145.6, 127.7, 124.6, 118.6 (q, $J_{C\text{-}F}$ = 320.7 Hz), 111.7, 92.0, 91.9, 29.0, 28.9, 27.0, 26.9, 23.3, 23.2, 14.2, 14.1, -0.582 ppm. **^{19}F NMR** (471 MHz, $CDCl_3$): δ -75.06 (s, 3F) ppm. **HRMS m/z (APCI)**: calcd. for $C_{22}H_{31}F_3O_4SSi$ $[M]^+$: 477.1737; found: 477.1732.

Preparation of 9,10-diphenyl-3-(trimethylsilyl)-9,10-dihydro-9,10-epoxyanthracen-2-yl trifluoromethanesulfonate (1u):

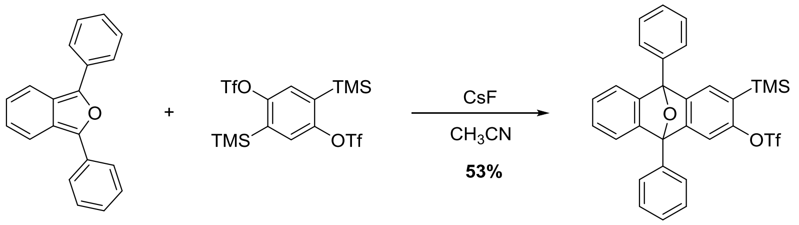

S4 **S2** **1u**

Under argon, CsF (456 mg, 3.0 mmol) was added to a solution of **S4** (810 mg, 3.0 mmol) and **S2** (1.04 g, 2.0 mmol) in dry CH_3CN (20 mL), the mixture was stirred at room temperature, monitored by TLC. After completion, the system was diluted with EA, passed through a short pad, evaporated the solvent and the residue was purified by flash column chromatography on silica gel to afford **1u** (600 mg, 1.06 mmol, 53% yield), R_f = 0.3 (hexane : CH_2Cl_2 = 4:1). **^{1}H NMR** (500 MHz, $CDCl_3$): δ 7.96-7.91 (m, 4H), 7.69-7.61 (m, 4H), 7.59-7.51 (m, 2H), 7.49 (s, 1H), 7.43 (s, 2H), 7.37 (s, 1H), 7.12 (s, 2H), 0.33 (s, 9H) ppm. **^{13}C NMR** (126 MHz, $CDCl_3$): δ 155.0, 153.0, 149.9, 149.6, 149.5, 134.4, 134.0, 129.8, 129.1, 129.1, 128.9, 128.7, 126.8, 126.7, 126.7, 126.4, 126.4, 121.1, 120.8, 118.5 (q, $J_{C\text{-}F}$ = 320.5 Hz), 113.0, 90.7, 90.6, -0.743 ppm. **^{19}F NMR** (471 MHz, $CDCl_3$): δ -75.08 (s, 3F) ppm. **HRMS m/z (APCI)**: calcd. for $C_{30}H_{25}F_3O_4SSi$ $[M]^+$: 567.1268; found: 567.1264.

Preparation of 1,2-bis(trifluoromethylation) estrone derivative 2w (*cf.* Scheme 3).

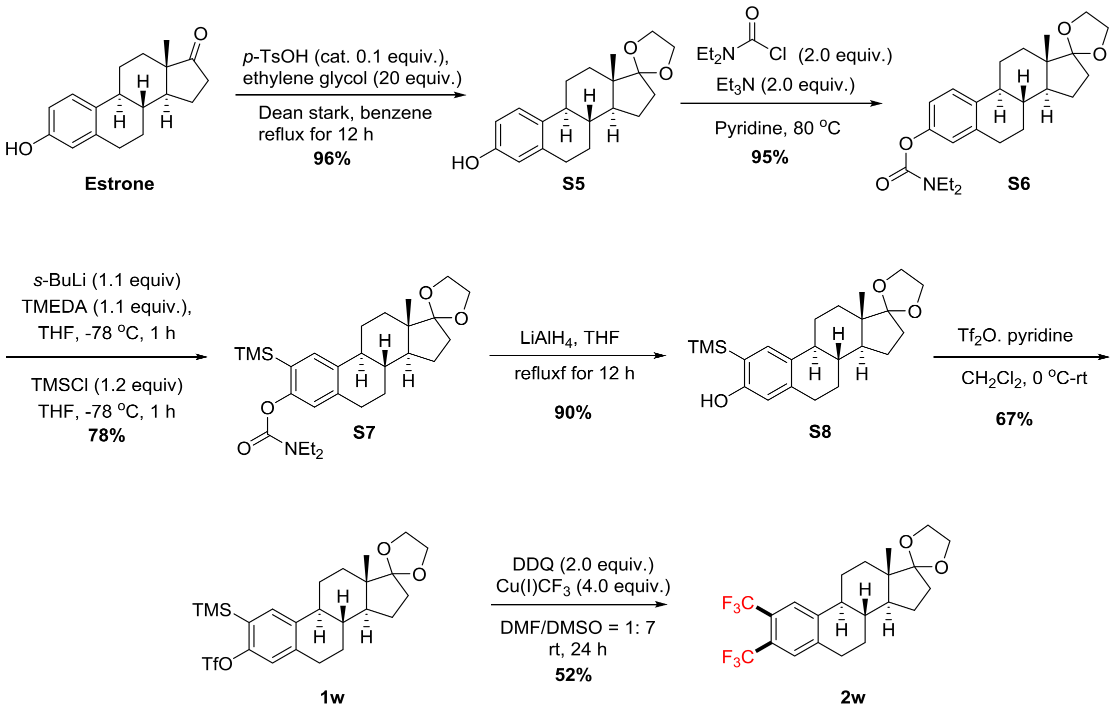

Solvent screening for the 1,2-bis(trifluoromethylation) of **1w.**[a]

entry	solvent (DMF : DMSO)	yield of **2w**[b]
1	1:1	9%
2	1:2	34%
3	1:3	44%
4	1:5	62%
5[c]	1:7	66%

[a]General condition: **1w** (0.1 mmol), DDQ (0.2 mmol), [$CuCF_3$] (0.4 mmol 1.0 mL in DMF). [b]Determined by ^{19}F NMR analysis using benotrifluoride as the internal standard. [c]DMF/ DMSO = 1.0 : 7.0 mL.

A mixture of estrone (1.28 g, 4.74 mmol), *p*-TsOH·H_2O (74.2 mg, 0.43 mmol), and ethylene glycol (5.30 mL, 94.8 mmol) in benzene (33 mL) was stirred under refluxing conditions in a Dean–Stark device for overnight. After cooling to room temperature, the mixture was poured into H_2O (65 mL) and extracted with CH_2Cl_2. The combined organic extracts were washed with H_2O and brine then dried over Na_2SO_4. The solvent was evaporated and the residue was purified by flash chromatography on silica gel (hexane–EtOAc, 2:1) to afforded **S5** as a colorless solid (1.43 g, 4.55 mmol, 96% yield). R_f = 0.4 (hexane : EtOAc = 2:1).[2q]

To a solution of **S5** (1.43 g, 4.55 mmol) in anhydrous pyridine (18 mL) was added diethylcarbamoyl chloride (1.15 mL, 9.10 mmol) and Et_3N (1.27 mL, 9.10 mmol) in one portion. The mixture was stirred at 80 °C overnight and then cooled to r.t. H_2O (100 mL) was added, the mixture was stirred for 30 min, and extracted with EtOAc. The combined organic phases were washed with H_2O and aq 1 M HCl for 5 times separately, then washed with brine, dried over Na_2SO_4. Filtered and the solvent was evaporated and the residue was purified by flash chromatography on silica gel (hexane–EtOAc, 2:1) to afforded **S6**

(1.79 g, 4.32 mmol, 95% yield). R_f = 0.5 (hexane : EtOAc = 2:1).[2q]

Under argon, a solution of carbamate **S6** (1.79 g, 4.33 mmol) in THF (15 mL) was added via a syringe to a stirred solution of *s*-BuLi (3.67 mL of 1.3 M solution in cyclohexane, 4.77 mmol) and TMEDA (715 µL, 0.165 mmol) in THF (30 mL) at –78 °C. The resulting greenish solution was stirred at –78 °C for 1 h, treated dropwise via a syringe with a solution of an TMSCl (607 µL, 4.77 mmol) in THF (15 mL), stirred at this temperature for 1 h, and then the reaction mixture was allowed to warm to r.t. The mixture was hydrolyzed with sat. aq NH_4Cl and extracted with EtOAc. The combined organic extracts were washed with brine and dried over Na_2SO_4. Filtered and concentration under reduced pressure then the residue was directly purified by flash chromatography on the silica gel using hexane–EtOAc as an eluent afforded the product **S7** (1.63 g, 3.37 mmol, 78% yield.). R_f = 0.5 (hexane : EtOAc = 4:1).[2q]

Under argon, a solution of **S7** (1.63 g, 3.37 mmol) in anhydrous THF (14 mL) was added at 0 °C to a stirred suspension of $LiAlH_4$ (640 mg, 16.9 mmol) in anhydrous THF (20 mL). After stirring under refluxing conditions for 24h, the mixture was cooled to r.t. and treated with H_2O (0.64 mL), 15% aq NaOH (0.64 mL), and H_2O (1.90 mL). After stirring for 10 min, the precipitate formed was filtered off and washed on the filter with EtOAc. The combined organic phases were dried over Na_2SO_4. Filtered and concentration under vacuum then the residue was directly purified by flash chromatography of the residue on silica gel afforded **S8** (1.17 g, 3.03 mmol, 90% yield). R_f = 0.5 (hexane : EtOAc = 5:1).[2q]

Under argon, to a solution of S8 (1.16 g, 3.00 mmol) and pyridine (0.36 mL, 4.5 mmol) in CH_2Cl_2 (12 mL) was slowly added Tf_2O (0.76 mL, 4.5 mmol) at 0 °C, stirred for 1 h and additional 30 mins at room temperature, quenched by sat. aq $NaHCO_3$, extracted with Et_2O, the combined organic layer was washed with H_2O, brine then dried over Na_2SO_4. Filtered and concentration under vacuum then the residue was directly purified by flash chromatography of the residue on silica gel afforded **1w** (1.07 g, 2.00 mmol, 67% yield). R_f = 0.6 (hexane : EtOAc = 5:1).[3] **^{1}H NMR** (400 MHz, $CDCl_3$): δ 7.42 (s, 1H), 7.02 (s, 1H), 3.97-3.91 (m, 4H), 2.87 (s, 2H), 2.36-2.27 (m, 2H), 2.04 (s, 1H), 1.94-1.77 (s, 4H), 1.65-1.26 (m, 6H), 0.890 (s, 3H), 0.345 (s, 9H) ppm. **^{13}C NMR** (126 MHz, $CDCl_3$): δ 153.2, 141.0, 139.9, 133.3, 128.7, 119.6, 119.4, 118.6 (q, J_{C-F} = 320.0 Hz), 65.4, 64.7, 49.5, 46.2, 44.0, 38.8, 34.3, 30.7, 29.8, 26.7, 26.0, 22.5, 14.4, -0.584 ppm. The spectral data are in full accordance with the literature report.[2q]

Under argon, to a 50 mL round-bottom flask equipped with a magnetic stir bar was added **1w** (207 mg, 0.4 mmol), DDQ (181.6 mg, 0.8 mmol) and DMSO (28 mL). A solution of [$CuCF_3$] in DMF (4.0 mL, 1.6 mmol) was added dropwise to the above mixture under argon at 0 °C. The reaction mixture was warmed up to room temperature and stirred under argon for 24 h, then quenched with saturated $NaHCO_3$ aqueous solution then neutralized with 1 M HCl, extracted with diethyl ether for three times. The organic layers were combined, washed with water then brine, dried over anhydrous Na_2SO_4, filtered and concentrated by rotary evaporator. The crude product was purified by preparative TLC to afford **2w** (90.2 mg, 0.21 mmol, 52%). R_f = 0.4 (hexane : acetone = 12:1). **^{1}H NMR** (500 MHz, $CDCl_3$): δ 7.72 (s, 1H), 7.52 (s, 1H), 3.99-3.90 (m, 4H), 2.98-2.89 (m, 2H), 2.39-2.30 (m, 2H), 2.07-1.96 (m, 2H), 1.89-1.76 (m, 3H), 1.69-1.49 (m, 3H), 1.48-1.31 (m, 3H), 0.89 (s, 3H) ppm. **^{13}C NMR** (126 MHz, $CDCl_3$): δ 145.0, 141.6, 128.6 (q, J_{C-F} = 3.5 Hz), 125.3 (m), 125.1 (q, J_{C-F} = 32.6 Hz), 123.4 (q, J_{C-F} = 274.8 Hz), 123.2 (q, J_{C-F} = 274.2 Hz), 119.3, 65.4 (m), 64.7(m), 49.4, 46.1, 44.1, 38.4, 34.3, 30.6, 29.4, 26.4, 25.8, 22.4, 14.4 (q, J_{C-F} = 2.1 Hz) ppm (one carbon missing due to overlap). **^{19}F NMR** (471 MHz, $CDCl_3$): δ -60.18 (q, J_{F-F} = 12.2 Hz, 3F), -60.39 (q, J_{F-F} = 12.2 Hz, 3F) ppm. **HRMS m/z (APCI)**: calcd. for $C_{22}H_{25}F_6O_2$ $[M+H]^+$: 435.1753; found: 435.1752.

3 Asgari, P.; Dakarapu, U. S.; Nguyen, H. H.; Jeon, J. *Tetrahedron* **2017**, *73*, 4052

Preparation of compound 3 (*cf.* Scheme 4a).

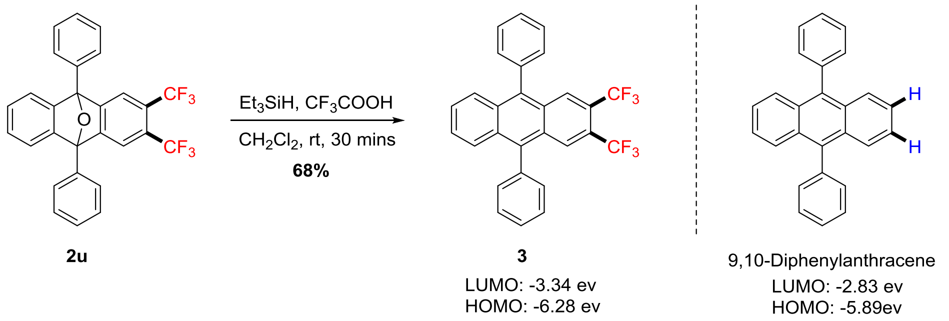

A glass vial equipped with a magnetic stir bar and a cap was charged with **2u** (96.4 mg, 0.2 mmol), wet CH_2Cl_2 (0.4 mL), Et_3SiH (48 µL, 0.3 mmol), CF_3COOH (20 µL, 0.3 mmol), then the via was sealed and stirred at room temperature for 3 h, after completion, quenched by adding water, extracted with Et_2O for 3 times, the combined organic phase was washed with H_2O, brine then dried over Na_2SO_4. Filtered and concentrated under reduced pressure. Purification of the residue by column chromatography afforded **3** (63.5 mg, 0.2 mmol, 68% yield). R_f = 0.7 (hexane : CH_2Cl_2 = 5:1). **^{1}H NMR** (400 MHz, $CDCl_3$): δ 8.32 (s, 2H), 7.85 (dd, *J* = 6.4 Hz, *J* = 2.8 Hz, 2H), 7.72-7.63 (m, 6H), 7.52-7.50 (m, 6H) ppm. **^{13}C NMR** (101 MHz, $CDCl_3$): δ 139.6, 137.2, 132.1, 131.2, 129.4, 129.0, 128.6, 128.3, 127.5, 127.1, 123.4 (q, J_{C-F} = 277.0 Hz), 122.4 (m) ppm **^{19}F NMR** (376 MHz, $CDCl_3$): δ -60.43 (s, 6F) ppm. **HRMS m/z (EI)**: calcd. for $C_{28}H_{16}F_6$ $[M]^+$: 466.1151; found: 466.1152.

Cyclic Voltammetry (CV):

Cyclic voltammetry was performed in a solution in CH_2Cl_2 on a PAR Potentiostat/Galvanostat Model 263A Electrochemical Station (Princeton Applied Research). The solution contained 0.1 M Bu_4NPF_6 as the supporting electrolyte. A platinum bead was used as a working electrode, a platinum wire was used as an auxiliary electrode, and a silver wire was used as a pseudo-reference. Ferrocene/ferrocenium was used as the internal standard. Potentials were referenced to ferrocenium/ferrocene ($FeCp_2^+/FeCp_2^0$).

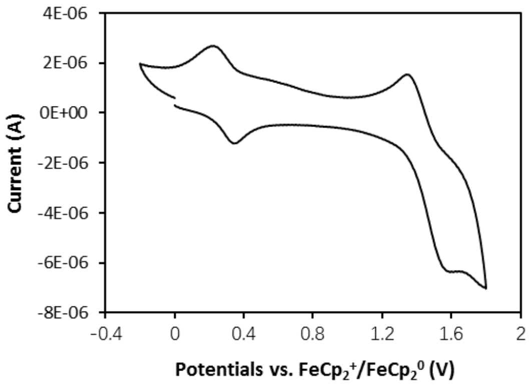

Figure 1: Cyclic voltammogram of **3**

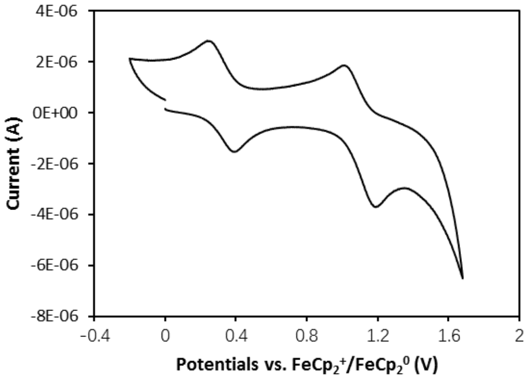

Figure 2: Cyclic voltammogram of 9,10-diphenylanthracene

UV-Vis absorption spectra (recorded on a Varian CARY 1E UV-vis spectrophotometer):

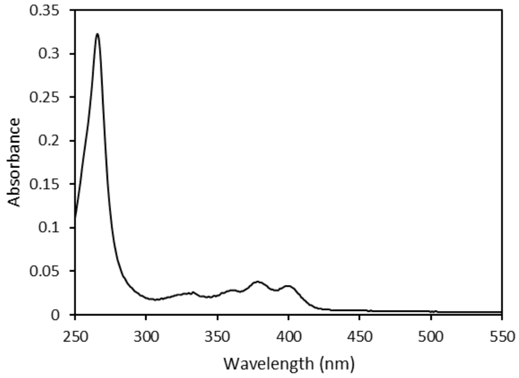

Figure 3: Absorption spectra of **3**

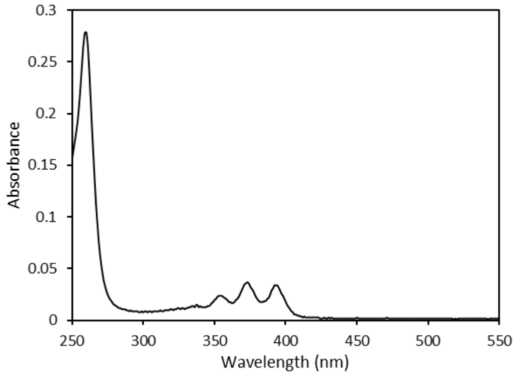

Figure 4: Absorption spectra of 9,10-diphenylanthracene

Summary of electrochemical potentials and energy levels of LUMO and HOMO

Compound	E_{ox} vs Fc^+/Fc (V)[a]	HOMO-LUMO Gap[b]	HOMO (eV)[c]	LUMO (eV)[d]
**3**	1.18	422 nm / 2.94	-6.28	-3.34
9,10-diphenylanthracene	0.79	405 nm / 3.06	-5.89	-2.83

[a]Half-wave potential versus ferrocenium/ferrocene for the oxidation wave. [b]λmax of the longest-

wavelength absorption in the UV-vis absorption spectrum from a solution in dicholromethane. [c]Estimated from HOMO = -5.10 - Eox (eV). [d]Calculated from the HOMO-LUMO gap and the HOMO energy level.[4]

Preparation of compound 4 and 5 (*cf.* Scheme 4b & 4c).

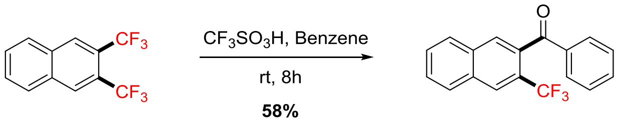

2e **4**

Under argon, to a solution of **2e** (52.8 mg, 0.2 mmol) in dry C_6D_6 (0.4 mL) was added CF_3SO_3H (0.4 mL) was added, the mixture was stirred at room temperature for 6 hours, after which it was poured over several grams of ice, extracted with CH_2Cl_2 for 3 times, washed with water and brine, dried over Na_2SO_4. Filtered and concentrated under reduced pressure. Purification of the residue by column chromatography afforded **4** (35 mg, 0.12 mmol, 58% yield). R_f = 0.3 (hexane : EtOAc = 8:1). **^{1}H NMR** (500 MHz, $CDCl_3$): δ 8.33 (s, 1H), 8.00 (dd, J = 6.0 Hz, J = 3.5 Hz, 1H), 7.88-7.86 (m, 4H), 7.68 (dd, J = 3.5 Hz, 2H), 7.62 (t, J = 7.5 Hz, 1H), 7.48 (t, J = 7.5 Hz, 2H) ppm. **^{13}C NMR** (126 MHz, $CDCl_3$): δ 195.6, 137.0, 134.3, 133.8, 133.3, 132.5, 130.5 (q, $J_{C\text{-}F}$ = 6.8 Hz), 129.3, 129.2, 129.1, 128.9 (q, $J_{C\text{-}F}$ = 6.6 Hz), 128.6, 128.3 (q, $J_{C\text{-}F}$ = 6.3 Hz), 128.0 (m), 125.8 (q, $J_{C\text{-}F}$ = 32.4 Hz), 123.9 (q, $J_{C\text{-}F}$ = 274.2 Hz) ppm **^{19}F NMR** (470 MHz, $CDCl_3$): δ -58.52 (s, 3F) ppm. **HRMS m/z (APCI)**: calcd. for $C_{18}H_{12}F_3O$ $[M+H]^+$: 301.0833; found: 301.0835.

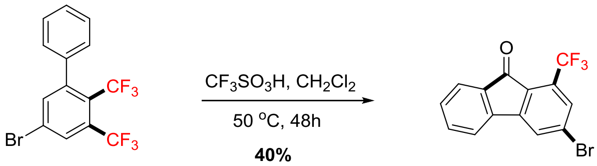

2p **5**

Under argon, a glass vial equipped with a magnetic stir bar was charged with **2p** (44.0 mg, 0.12 mmol), CH_2Cl_2 (0.4 mL), then CF_3SO_3H (106 µL, 1.2 mmol) was added, the vial was sealed and the mixture was heated at 50 °C for 48 hours, then cooled to room temperature, quenched with sat. aq. $NaHCO_3$, extracted with CH_2Cl_2 for 3 times, washed with water and brine, dried over Na_2SO_4. Filtered and concentrated under reduced pressure. Purification of the residue by column chromatography afforded **5** (18.1 mg, 0.056 mmol, 40% yield). R_f = 0.4 (hexane : EtOAc = 5:1). **^{1}H NMR** (400 MHz, $CDCl_3$): δ 7.87 (s, 1H), 7.72-7.70 (m, 2H), 7.55 (q, J = 6.7 Hz, 1H), 7.54 (s, 1H), 7.40 (dt, J = 7.0 Hz, J = 2.0 Hz, 1H) ppm. **^{13}C NMR** (101 MHz, $CDCl_3$): δ 188.9, 148.2, 141.8, 135.2, 134.8, 133.6, 130.8, 129.4, 129.2 (q, $J_{C\text{-}F}$ = 35.8 Hz), 129.1 (q, $J_{C\text{-}F}$ = 23.6 Hz), 127.0, 125.9, 121.7 (q, $J_{C\text{-}F}$ = 275.7 Hz), 120.7 ppm **^{19}F NMR** (376 MHz, $CDCl_3$): δ -62.90 (s, 3F) ppm. **HRMS m/z (APCI)**: calcd. for $C_{14}H_7BrF_3O$ $[M+H]^+$: 326.9626; found: 326.9627.

Preparation of compound 6 (*cf.* Scheme 4d).

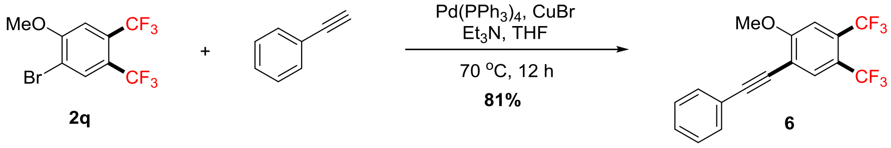

2q **6**

Under argon, a schlenk tube equipped with a magnetic stir bar was charged with $Pd(PPh_3)_4$ (2.2 mg,

4 Cardona, C. M.; Li, W.; Kaifer, A. E.; Stockdale, D.; Bazan, G. C. *Adv. Mater.* **2011**, *23*, 2367.

0.0019 mmol), CuBr (0.5 mg, 0.0038 mmol), then THF (0.2 mL) was added and the suspension was stirred for 5 mins followed by addition of **2q** (30.0 mg, 0.093 mmol) and Et_3N (0.14 mL), after 5 mins more stirring, phenylacetylene (12.3 μL, 0.11 mmol) was added. The reaction system was cooled to -78 °C, pumped and refilled with argon for 3 times, after which the tube was sealed and heated at 70 °C for 12 h. The solution was cooled to room temperature, filtered through a layer of celite on top of silica and eluted with diethyl ether. The solvent was removed in vacuo and the crude material was purified via column chromatography to yield **6** (26.0 mg, 0.076 mmol, 81% yield). R_f = 0.5 (hexane : CH_2Cl_2 = 8:1). **^{1}H NMR** (500 MHz, $CDCl_3$): δ 7.94 (s, 2H), 7.59-7.58 (m, 2H), 7.39-7.38 (m, 3H), 7.29 (s, 1H) ppm. **^{13}C NMR** (126 MHz, $CDCl_3$): δ 161.8, 133.2 (q, $J_{C\text{-}F}$ = 6.0 Hz), 132.0, 129.2, 128.9 (q, $J_{C\text{-}F}$ = 35.5 Hz), 128.6, 122.8 (q, $J_{C\text{-}F}$ = 273.2 Hz), 122.6 (q, $J_{C\text{-}F}$ = 274.4 Hz), 122.5, 120.4 (q, $J_{C\text{-}F}$ = 33.4 Hz), 116.5, 110.2 (q, $J_{C\text{-}F}$ = 6.2 Hz), 97.2, 83.2, 56.6 ppm **^{19}F NMR** (470 MHz, $CDCl_3$): δ -59.52 (q, $J_{F\text{-}F}$ = 12.7 Hz, 3F), -60.47 (q, $J_{F\text{-}F}$ = 12.7 Hz, 3F) ppm. **HRMS m/z (EI)**: calcd. for $C_{17}H_{10}F_6O$ $[M]^+$: 344.0530; found: 344.0630.

Mechanistic studies:

^{19}F NMR experiment (*cf.* Scheme 5a).

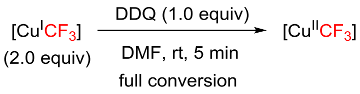

Under argon, to an NMR tube charged with DDQ (11.4 mg, 0.05 mmol) and freshly prepared [$CuCF_3$] (0.1 mmol in 0.4 mL DMF) then sealed with a cap. The mixture was monitored by ^{19}F NMR using benzotrifluoride as the internal standard over 5 min at room temperature.

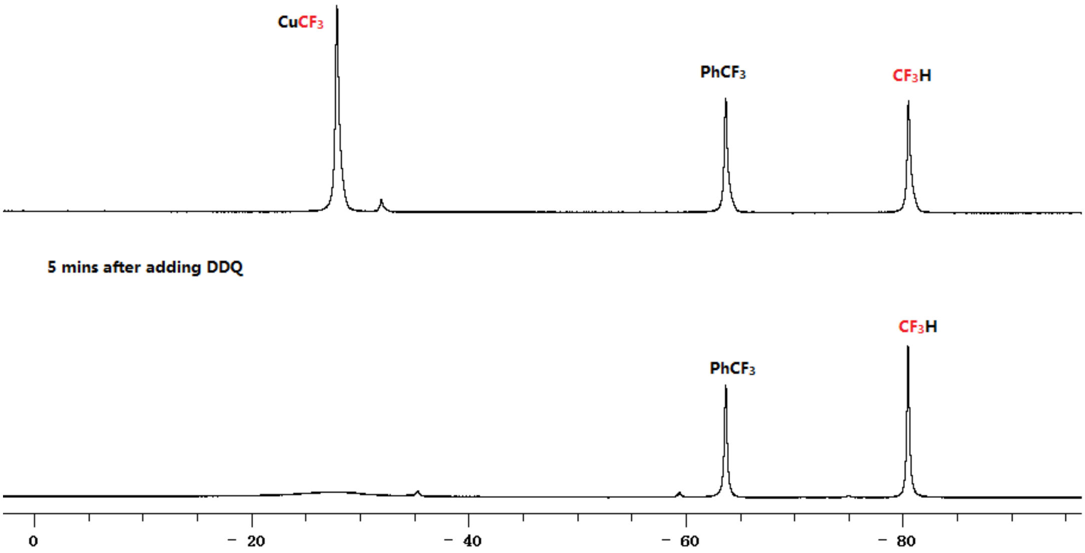

Radical scavenger experiment (*cf.* Scheme 5c):

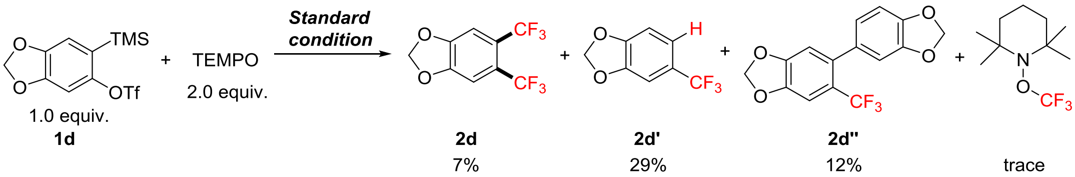

Under argon, to a glass tube equipped with a magnetic stir bar was added **1d** (34.2 mg, 0.1 mmol), DDQ (45.4 mg, 0.2 mmol), TEMPO (31.2 mg, 0.2 mmol) and DMSO (2.0 mL). A solution of [$CuCF_3$]

in DMF (1.0 mL, 0.4 mmol) was added dropwise to the above mixture under argon at 0 °C. The reaction mixture was warmed up to room temperature and stirred under argon for 24 h, The crude yield of each product was analyzed by ^{19}F NMR using benzotrifluoride as the internal standard.

Isolation of 2d”: quenched with saturated $NaHCO_3$ aqueous solution then neutralized with 1 M HCl, extracted with diethyl ether for three times. The organic layers were combined, washed with water then brine, dried over anhydrous Na_2SO_4, filtered and concentrated by rotary evaporator. The crude product was purified by flash column chromatography on silica gel to afford **2d”**. R_f = 0.3 (hexane : CH_2Cl_2 = 10:1). **^{1}H NMR** (500 MHz, $CDCl_3$): δ 7.14 (s, 1H), 6.81 (d, J = 8.0 Hz, 1H), 6.77 (s, 1H), 6.75-6.71 (m, 2H), 6.07 (s, 2H), 6.00 (s, 2H) ppm. **^{13}C NMR** (126 MHz, $CDCl_3$): δ 149.6, 147.2, 147.1, 146.9, 136.4, 133.4, 124.1 (q, $J_{C\text{-}F}$ = 273.7 Hz), 122.8, 122.2 (q, $J_{C\text{-}F}$ = 30.4 Hz), 112.2, 110.0, 107.8, 106.5 (q, $J_{C\text{-}F}$ = 5.7 Hz), 102.2, 101.3 ppm **^{19}F NMR** (470 MHz, $CDCl_3$): δ-56.32 (s, 3F) ppm. **HRMS m/z (EI)**: calcd. for $C_{15}H_9F_3O_4$ $[M]^+$: 310.0447; found: 310.0446.

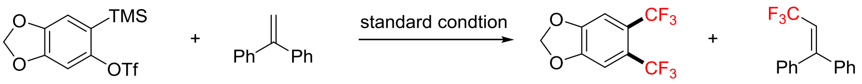

1.0 equiv. 3.0 equiv.
1d **2d** 78% trace

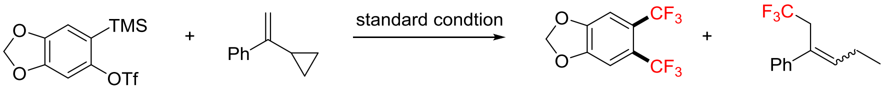

1.0 equiv. 3.0 equiv.
1d **2d** 62% trace

Under argon, to a glass tube equipped with a magnetic stir bar was added **1d** (34.2 mg, 0.1 mmol), DDQ (45.4 mg, 0.2 mmol), alkene (0.3 mmol) and DMSO (2.0 mL). A solution of $[CuCF_3]$ in DMF (1.0 mL, 0.4 mmol) was added dropwise to the above mixture under argon at 0 °C. The reaction mixture was warmed up to room temperature and stirred under argon for 24 h, The result was analyzed by ^{19}F NMR using benzotrifluoride as the internal standard.

Radical clock experiment (*cf.* Scheme 5d):

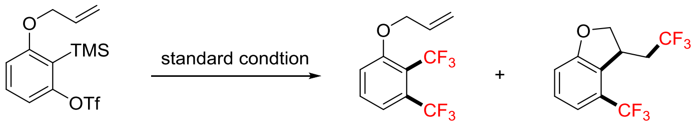

1x **2x** 16% (^{19}F NMR) **8** 5% (^{19}F NMR)

Under argon, to a 25 mL round-bottom flask equipped with a magnetic stir bar was added **1x** (141.6 mg, 0.4 mmol), DDQ (181.6 mg, 0.8 mmol) and DMSO (8.0 mL). A solution of $[CuCF_3]$ in DMF (4.0 mL, 1.6 mmol) was added dropwise to the above mixture under argon at 0 °C. The reaction mixture was warmed up to room temperature and stirred under argon for 24 h, The crude yield of each product was analyzed by ^{19}F NMR using benzotrifluoride as the internal standard. then quenched with saturated $NaHCO_3$ aqueous solution then neutralized with 1 M HCl, extracted with diethyl ether for three times. The organic layers were combined, washed with water then brine, dried over anhydrous Na_2SO_4, filtered and concentrated by rotary evaporator. The crude product was purified by flash column chromatography on silica gel to afford product **2x** and **8** (17.0 mg, 0.063 mmol) as inseparable mixure. R_f = 0.4 (hexane : CH_2Cl_2 = 8:1). **Compound 2x: ^{1}H NMR** (500 MHz, $CDCl_3$): δ 7.56 (t, J = 8.0 Hz, 1H), 7.43 (d, J = 8.0

Hz, 1H), 7.24 (d, J = 8.5 Hz, 1H), 6.07-5.99 (m, 2H), 5.48 (d, J = 17.0 Hz, 1H), 5.33 (d, J = 10.5 Hz, 1H), 4.67 (d, J = 4.5 Hz, 2H) ppm. **^{13}C NMR** (126 MHz, $CDCl_3$): δ 158.6 (q, $J_{C\text{-}F}$ = 1.8 Hz), 132.8, 131.9, 129.7 (q, $J_{C\text{-}F}$ = 32.8 Hz), 123.0 (q, $J_{C\text{-}F}$ = 274.3 Hz), 122.7 (q, $J_{C\text{-}F}$ = 275.0 Hz), 119.4 (q, $J_{C\text{-}F}$ = 7.2 Hz), 118.2, 118.1, 117.7 (q, $J_{C\text{-}F}$ = 32.8 Hz), 70.4 ppm **^{19}F NMR** (470 MHz, $CDCl_3$): δ -57.96 (q, $J_{F\text{-}F}$ = 16.0 Hz, 3F), -59.03 (q, $J_{F\text{-}F}$ = 16.0 Hz, 3F) ppm. **Compound 8: ^{1}H NMR** (500 MHz, $CDCl_3$): δ 7.30 (t, J = 8.0 Hz, 1H), 7.16 (d, J = 7.5 Hz, 1H), 7.02 (d, J = 8.0 Hz, 1H), 4.63 (d, J = 9.5 Hz, 1H), 4.55 (t, J = 8.5 Hz, 1H), 3.97 (t, J = 9.3 Hz, 1H), 2.54-2.33 (m, 2H) ppm. **^{13}C NMR** (126 MHz, $CDCl_3$): δ 160.8, 130.0, 127.3 (q, $J_{C\text{-}F}$ = 32.6 Hz), 124.1 (q, $J_{C\text{-}F}$ = 273.4 Hz), 124.0 (q, $J_{C\text{-}F}$ = 278.0 Hz), 118.5 (q, $J_{C\text{-}F}$ = 4.8 Hz), 113.9, 76.2, 37.8 (q, $J_{C\text{-}F}$ = 27.6 Hz), 36.4, 30.5 ppm **^{19}F NMR** (470 MHz, $CDCl_3$): δ -61.25 (s, 3F), -65.90 (t, J = 10.8 Hz, 3F) ppm. **HRMS m/z (EI)**: calcd. for $C_{11}H_8F_6O$ $[M]^+$: 270.0474; found: 270.0473.

Table S1: Optimization Studies for 1,2-Bis(trifluoromethylation) of Aryne Precursor **1a**[a]

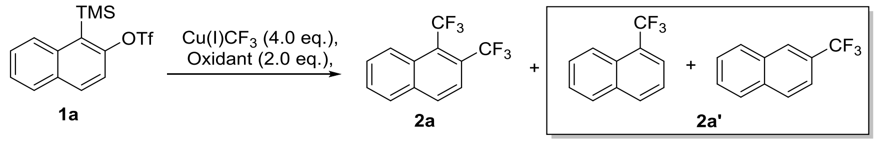

entry	oxidant	additive	solvent	temp (°C)	yield (%)[b]
1[c]	air	none	DMF	50	**2a** (30%), **2a'** (trace)
2	none	none	DMF	50	**2a** (0%)
3	$Cu(OAc)_2$	none	DMF	50	**2a** (7%), **2a'** (23%)
4	Ag_2CO_3	none	DMF	50	**2a** (9%), **2a'** (8 %)
5	AgOAc	none	DMF	50	**2a** (26%), **2a'** (21%)
6	BQ	none	DMF	50	**2a** (4%), **2a'** (4%)
7	$PhI(OAc)_2$	none	DMF	50	**2a** (26%), **2a'** (29%)
8	DDQ	none	DMF	50	**2a** (58%), **2a'** (10%)
9	DDQ	K_2CO_3	DMF	50	**2a** (28%), **2a'** (20%)
10	DDQ	20 mg 4A MS	DMF	50	**2a** (40%), **2a'** (34%)
11	DDQ	50 mg 4A MS	DMF	50	**2a** (41%), **2a'** (29%)
12	DDQ	1.5 eq. TBAF $3H_2O$	DMF	50	**2a** (50%), **2a'** (13%)
13[d]	DDQ	none	DMF	50	**2a** (50%), **2a'** (25%)
14[e]	DDQ	none	DMF	50	**2a** (26%), **2a'** (40%)
15[f]	DDQ	none	DMF	50	**2a** (51%), **2a'** (17%)
16[g]	DDQ	none	DMF	50	**2a** (39%), **2a'** (7%)
17[h]	DDQ	none	DMF	50	**2a** (46%), **2a'** (25%)
18[i]	DDQ	none	DMF	50	**2a** (28%), **2a'** (15%)
19	DDQ	none	CH_3CN	50	**2a** (19%), **2a'** (42%)
20	DDQ	none	Dioxane	50	**2a** (10%), **2a'** (60%)
21	DDQ	none	Toluene	50	**2a** (4.0%), **2a'** (39%)
22	DDQ	none	NMP	50	**2a** (56%), **2a'** (17%)
23[j]	DDQ	none	DMSO	50	**2a** (77%), **2a'** (trace)
24[j]	DDQ	none	DMSO	rt	**2a** (78%), **2a'** (5%)
25[k]	DDQ	none	DMSO	rt	**2a** (62%), **2a'** (12%)
26[l]	DDQ	none	DMSO	rt	**2a** (77%), **2a'** (trace)

[a]Unless specified otherwise, reactions were carried out using **1a** (0.1 mmol), [$CuCF_3$] (0.4 mmol in 1.0 mL DMF), oxidant (0.2 mmol) and DMF (1.0 mL). [b]Determined by ^{19}F NMR analysis using benotrifluoride as the internal standard. [c]Reaction was open to air. [d][$CuCF_3$] was stabilized with olah's

reagent. [e][CuCF$_3$] stabilized with Et_3N HCl and extra 4.0 eq. KF; [f]Using 0.3 mmol [CuCF$_3$]. [g]Using 0.1 mmol DDQ. [h]Using 0.3 mmol DDQ. [i]Using 0.4 mmol DDQ. [j]DMF/DMSO=1.0 : 1.0 mL. [k]DMF/DMSO=1.0 : 0.5 mL. [l]DMF/DMSO=1.0 : 2.0 mL. BQ = 1,4-Benzoquinone. DDQ = 2,3-Dichloro-5,6-Dicyanobenzoquinone.

Characterization data:

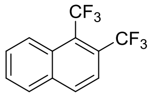

2a: 1,2-bis(trifluoromethyl)naphthalene. Prepared according to the general procedure. Reaction was run using **1a** (139.2 mg, 0.4 mmol), DDQ (181.6 mg, 0.8 mmol), DMSO (8.0 mL), [$CuCF_3$] in DMF solution (4.0 mL, 1.6 mmol). The product was purified by flash column chromatography on silica gel (hexane) and obtained a colorless oil (0.31 mmol, 81.3 mg, 77%), R_f = 0.60 (hexane). **^{1}H NMR** (400 MHz, $CDCl_3$): δ 8.37-8.34 (m, 1H), 8.07 (d, J = 8.8 Hz, 1H), 7.92-7.90 (m, 1H), 7.83 (d, J = 8.8 Hz, 1H), 7.70-7.64 (m, 2H) ppm. **^{13}C NMR** (101 MHz, $CDCl_3$): δ 135.2, 133.1, 130.1 (q, J_{C-F} = 1.4 Hz), 128.7, 128.5, 127.3 (q, J_{C-F} = 35.0 Hz), 126.2 (q, J_{C-F} = 4.7 Hz), 125.9 (q, J_{C-F} = 34.2 Hz), 123.7 (q, J_{C-F} = 277.1 Hz), 123.7 (q, J_{C-F} = 277.1 Hz), 123.5 (q, J_{C-F} = 275.3 Hz), 122.2 (q, J_{C-F} = 7.0 Hz) ppm. **^{19}F NMR** (470 MHz, $CDCl_3$): δ -53.70 (q, J_{F-F} = 16.5 Hz, 3F), -57.65 (q, J_{F-F} = 16.5 Hz, 3F) ppm. **HRMS m/z (APCI)**: calcd. for $C_{12}H_6F_6$ $[M]^+$: 264.0368; found: 264.0364.

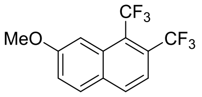

2b: 7-methoxy-1,2-bis(trifluoromethyl)naphthalene. Prepared according to the general procedure. Reaction was run using **1b** (151.2 mg, 0.4 mmol), DDQ (181.6 mg, 0.8 mmol), DMSO (8.0 mL), [$CuCF_3$] in DMF solution (4.0 mL, 1.6 mmol). The product was purified by flash column chromatography on silica gel (hexane) and obtained a white solid (0.33 mmol, 96.4 mg, 82%), R_f= 0.60 (hexane : CH_2Cl_2 = 8 : 1). **^{1}H NMR** (500 MHz, $CDCl_3$): δ 7.97-7.93 (m, 1H), 7.79-7.75 (m, 1H), 7.67 (d, J = 8.5 Hz, 1H), 7.56 (s, 1H), 7.29 (d, J = 9.0 Hz, 1H) ppm. **^{13}C NMR** (126 MHz, $CDCl_3$): δ 159.4, 132.6 (q, J_{C-F} = 7.9 Hz), 131.8, 131.0, 130.0 (q, J_{C-F} = 7.3 Hz), 127.8 (q, J_{C-F} = 33.0 Hz), 123.9 (q, J_{C-F} = 32.3 Hz), 123.9 (q, J_{C-F} = 276.3 Hz), 123.6 (q, J_{C-F} = 274.9 Hz), 121.7, 120.0 (m), 104.4 (m), 55.4 (q, J_{C-F} = 16.3 Hz) pspm. **^{19}F NMR** (470 MHz, $CDCl_3$): δ -54.97 (q, J_{F-F} = 16.9 Hz, 3F), -57.84 (q, J_{F-F} = 16.9 Hz, 3F) ppm. **HRMS m/z (APCI)**: calcd. for $C_{13}H_9F_6O$ $[M+H]^+$: 295.0552; found: 295.0552.

1 mmol scale reaction: Prepared according to the general procedure. Reaction was run using **1b** (378 mg, 1.0 mmol), DDQ (548 mg, 2.0 mmol), DMSO (20.0 mL), [$CuCF_3$] in DMF solution (10.0 mL, 4.0 mmol). The product was purified by flash column chromatography on silica gel (hexane) and obtained a white solid (0.67 mmol, 197 mg, 67%), R_f = 0.60 (hexane : CH_2Cl_2 = 8 : 1).

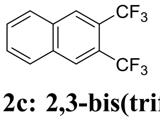

2c: 2,3-bis(trifluoromethyl)naphthalene. Prepared according to the general procedure. Reaction was run using **1c** (139.2 mg, 0.4 mmol), DDQ (181.6 mg, 0.8 mmol), DMSO (8.0 mL), [$CuCF_3$] in DMF solution (4.0 mL, 1.6 mmol). The product was purified by flash column chromatography on silica gel (hexane) and obtained a colorless oil (0.25 mmol, 65.4 mg, 62%), Rf = 0.60 (hexane). **^{1}H NMR** (500 MHz, $CDCl_3$): δ 8.33 (s, 2H), 7.98-7.96 (m, 2H), 7.74-7.72 (m, 2H) ppm. **^{13}C NMR** (126 MHz, $CDCl_3$): δ 133.1, 129.7, 129.4 (m), 128.8, 124.0 (m), 123.3 (q, J_{C-F} = 274.7 Hz) ppm. **^{19}F NMR** (470 MHz, $CDCl_3$): δ -60.09 (s, 6F) ppm. **HRMS m/z (APCI)**: calcd. for $C_{12}H_6F_6$ $[M]^+$: 264.0368; found: 264.0369.

2 mmol scale reaction: Prepared according to the general procedure. Reaction was run using **1b** (696 mg, 2.0 mmol), DDQ (1.096 g, 4.0 mmol), DMSO (40.0 mL), [$CuCF_3$] in DMF solution (20.0 mL, 8.0

mmol). The product was purified by flash column chromatography on silica gel (hexane) and obtained a colorless oil (1.0 mmol, 264 mg, 50%), R_f = 0.60 (hexane : CH_2Cl_2 = 8 : 1).

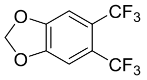

2d: 5,6-bis(trifluoromethyl)benzo[d][1,3]dioxole. Prepared according to the general procedure. Reaction was run using **1d** (139.2 mg, 0.4 mmol), DDQ (181.6 mg, 1.20 mmol), DMSO (8.0 mL), [$CuCF_3$] in DMF solution (4.0 mL, 1.6 mmol). The product was purified by flash column chromatography on silica gel (hexane) and obtained a colorless oil (0.31 mmol, 84.5 mg, 78%), R_f = 0.40 (hexane). **^{1}H NMR** (500 MHz, $CDCl_3$): δ 7.25 (s, 2H), 6.15 (s, 2H) ppm. **^{13}C NMR** (126 MHz, $CDCl_3$): δ 150.3, 123.3 (m), 122.8 (q, $J_{C\text{-}F}$ = 277.1 Hz), 108.4, 103.3 ppm. **^{19}F NMR** (470 MHz, $CDCl_3$): δ -59.18 (s, 6F) ppm. **HRMS m/z (APCI)**: calcd. for $C_9H_9F_6O_2$ $[M]^+$: 258.0110; found: 258.0112.

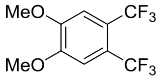

2e: 1,2-dimethoxy-4,5-bis(trifluoromethyl)benzene. Prepared according to the general procedure. Reaction was run using **1e** (143.2 mg, 0.4 mmol), DDQ (181.6 mg, 0.8 mmol), DMSO (12.0 mL), [$CuCF_3$] in DMF solution (4.0 mL, 1.6 mmol). The product was purified by flash column chromatography on silica gel (hexane/EtOAc) and obtained a colorless solid (0.25 mmol, 68.0 mg, 62%), R_f= 0.30 (hexane : EtOAc = 5 : 1). **^{1}H NMR** (500 MHz, $CDCl_3$): δ 7.23 (s, 2H), 3.96 (s, 6H) ppm. **^{13}C NMR** (126 MHz, $CDCl_3$): δ 150.9, 123.0 (q, $J_{C\text{-}F}$ = 274.6 Hz), 121.1 (m), 110.5, 56.4 ppm. **^{19}F NMR** (470 MHz, $CDCl_3$): δ -59.18 (s, 6F) ppm. **HRMS m/z (APCI)**: calcd. for $C_{10}H_8F_6O_2$ $[M]^+$: 274.0423; found: 274.0428.

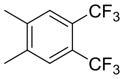

2f: 1,2-dimethyl-4,5-bis(trifluoromethyl)benzene. Prepared according to the general procedure. Reaction was run using **1f** (130.4 mg, 0.4 mmol), DDQ (181.6 mg, 0.8 mmol), DMSO (12.0 mL), [$CuCF_3$] in DMF solution (4.0 mL, 1.6 mmol). The product was purified by flash column chromatography on silica gel (hexane) and obtained a colorless oil (0.20 mmol, 48.4 mg, 50%), R_f = 0.60 (hexane). **^{1}H NMR** (500 MHz, $CDCl_3$): δ 7.58 (s, 2H), 2.37 (s, 6H) ppm. **^{13}C NMR** (126 MHz, $CDCl_3$): δ 141.3, 129.1 (m), 125.5 (m), 123.2 (q, $J_{C\text{-}F}$ = 274.9 Hz), 19.8 (q, $J_{C\text{-}F}$ = 7.8 Hz) ppm. **^{19}F NMR** (470 MHz, $CDCl_3$): δ -60.14 (s, 6F) ppm. **HRMS m/z (APCI)**: calcd. for $C_{10}H_8F_6$ $[M]^+$: 242.0523; found: 242.0524.

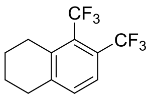

2g: 5,6-bis(trifluoromethyl)-1,2,3,4-tetrahydronaphthalene. Prepared according to the general procedure. Reaction was run using **1g** (144.4 mg, 0.4 mmol), DDQ (181.6 mg, 0.8 mmol), DMSO (8.0 mL), [$CuCF_3$] in DMF solution (4.0 mL, 1.6 mmol). The product was purified by preparative TLC (hexane) and obtained a colorless oil (0.21 mmol, 55.7 mg, 52%), R_f= 0.60 (hexane). **^{1}H NMR** (500 MHz, $CDCl_3$): δ 7.58 (d, J = 8.0 Hz, 1H), 7.33 (d, J = 8.5 Hz, 1H), 3.00-2.94 (m, 2H), 2.92-2.87 (m, 2H), 1.84-1.77 (m, 4H) ppm. **^{13}C NMR** (126 MHz, $CDCl_3$): δ 143.6, 139.7, 132.5, 127.3 (q, $J_{C\text{-}F}$ = 30.4 Hz), 126.4 (q, $J_{C\text{-}F}$ = 32.4 Hz), 124.6 (m), 123.8 (q, $J_{C\text{-}F}$ = 276.6 Hz), 123.6 (q, $J_{C\text{-}F}$ = 274.2 Hz), 30.2 (t, $J_{C\text{-}F}$ = 15.5 Hz), 27.5 (m), 22.6, 21.7 ppm. **^{19}F NMR** (470 MHz, $CDCl_3$): δ -56.22 (q, $J_{F\text{-}F}$ = 16.9 Hz, 3F), -

58.63 (q, J_{F-F} = 16.9 Hz, 3F) ppm. **HRMS m/z (ACPI)**: calcd. for $C_{12}H_{10}F_6$ $[M]^+$: 268.0681; found: 268.0683.

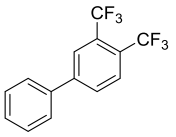

2h: 3,4-bis(trifluoromethyl)-1,1'-biphenyl. Prepared according to the general procedure. Reaction was run using **1f** (149.6 mg, 0.4 mmol), DDQ (181.6 mg, 0.8 mmol), DMSO (8.0 mL), $[CuCF_3]$ in DMF solution (4.0 mL, 1.6 mmol). The product was purified by flash column chromatography on silica gel (hexane) and obtained a colorless oil (0.31 mmol, 90.5 mg, 78%), R_f = 0.60 (hexane). **^{1}H NMR** (500 MHz, $CDCl_3$): δ 8.09 (s, 1H), 7.94 (d, J = 8.0 Hz, 1H), 7.88 (d, J = 8.0 Hz, 1H), 7.64 (d, J = 7.5 Hz, 2H), 7.53 (t, J = 7.5 Hz, 2H), 7.50 (t, J = 7.5 Hz, 1H) ppm. **^{13}C NMR** (126 MHz, $CDCl_3$): δ 145.4, 138.3, 130.3, 129.4, 129.2, 128.8 (q, J_{C-F} = 34.0 Hz), 128.7 (q, J_{C-F} = 5.9 Hz), 127.4, 126.7 (q, J_{C-F} = 33.1 Hz), 126.7 (q, J_{C-F} = 6.0 Hz), 123.2 (q, J_{C-F} = 275.1 Hz), 123.1 (q, J_{C-F} = 271.5 Hz) ppm. **^{19}F NMR** (470 MHz, $CDCl_3$): δ -60.07 (q, J_{F-F} = 12.7 Hz, 3F), -60.35 (q, J_{F-F} = 12.7 Hz, 3F) ppm. **HRMS m/z (APCI)**: calcd. for $C_{14}H_8F_6$ $[M]^+$: 290.0525; found: 290.0525.

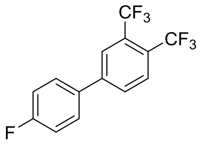

2i: 4'-fluoro-3,4-bis(trifluoromethyl)-1,1'-biphenyl. Prepared according to the general procedure. Reaction was run using **1i** (156.8 mg, 0.4 mmol), DDQ (181.6 mg, 0.8 mmol), DMSO (8.0 mL), $[CuCF_3]$ in DMF solution (4.0 mL, 1.6 mmol). The product was purified by flash column chromatography on silica gel (hexane) and obtained a colorless oil (0.32 mmol, 98.6 mg, 80%), R_f = 0.60 (hexane). **^{1}H NMR** (400 MHz, $CDCl_3$): δ 8.01 (s, 1H), 7.92 (d, J = 8.0 Hz, 1H), 7.83 (d, J = 8.0 Hz, 1H), 7.62-7.58 (m, 2H), 7.20 (t, J = 8.6 Hz, 2H) ppm. **^{13}C NMR** (101 MHz, $CDCl_3$): δ 163.6 (d, J_{C-F} = 250.4 Hz), 144.4, 134.4 (q, J_{C-F} = 3.2 Hz), 130.2, 129.2 (d, J_{C-F} = 8.4 Hz), 128.9 (q, J_{C-F} = 35.4 Hz), 128.7 (q, J_{C-F} = 5.9 Hz), 126.8 (q, J_{C-F} = 33.4 Hz), 126.5 (q, J_{C-F} = 6.0 Hz), 123.1 (q, J_{C-F} = 276.9 Hz), 123.0 (q, J_{C-F} = 275.3 Hz) 116.4 (d, J_{C-F} = 21.9 Hz), ppm. **^{19}F NMR** (376 MHz, $CDCl_3$): δ -61.29 (q, J_{F-F} = 12.8 Hz, 3F), -61.58 (q, J_{F-F} = 12.8 Hz, 3F), -114.7 (m) ppm. **HRMS m/z (APCI)**: calcd. for $C_{14}H_7F_7$ $[M]^+$: 308.0431; found: 308.0427.

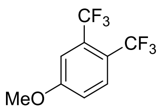

2j: 4-methoxy-1,2-bis(trifluoromethyl)benzene. Prepared according to the general procedure. Reaction was run using **1j** (131.2 mg, 0.4 mmol), DDQ (181.6 mg, 0.8 mmol), DMSO (8.0 mL), $[CuCF_3]$ in DMF solution (4.0 mL, 1.6 mmol). The product was purified by flash column chromatography on silica gel (hexane) and obtained a colorless oil (60% ^{19}F NMR yield), R_f = 0.40 (hexane). **^{1}H NMR** (500 MHz, $CDCl_3$): δ 7.77 (d, J = 8.5 Hz, 1H), 7.33 (s, 1H), 7.10 (d, J = 8.5 Hz, 1H), 3.90 (s, 2H) ppm. **^{13}C NMR** (101 MHz, $CDCl_3$): δ 162.0, 130.0 (q, J_{C-F} = 6.1 Hz), 129.9 (q, J_{C-F} = 33.7 Hz), 123.2 (q, J_{C-F} = 273.2Hz), 122.7 (q, J_{C-F} = 274.8 Hz), 120.1 (q, J_{C-F} = 35.2 Hz), 115.9, 114.7 (q, J_{C-F} = 6.2 Hz), 55.9 ppm. **^{19}F NMR**

(470 MHz, $CDCl_3$): δ -59.27 (q, J_{F-F} = 12.7 Hz, 3F), -60.72 (q, J_{F-F} = 12.7 Hz, 3F) ppm. **HRMS m/z (APCI)**: calcd. for $C_9H_9F_6O$ $[M]^+$: 244.0317; found: 244.0316.

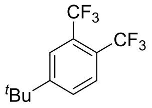

2k: 4-(tert-butyl)-1,2-bis(trifluoromethyl)benzene. Prepared according to the general procedure. Reaction was run using **1k** (141.6 mg, 0.4 mmol), DDQ (181.6 mg, 0.8 mmol), DMSO (8.0 mL), $[CuCF_3]$ in DMF solution (4.0 mL, 1.6 mmol). The product was purified by flash column chromatography on silica gel (hexane) and obtained a colorless oil (0.24 mmol, 64.8 mg, 60%), R_f = 0.60 (hexane). **^{1}H NMR** (500 MHz, $CDCl_3$): δ 7.84 (s, 1H), 7.77 (d, J = 8.0 Hz, 1H), 7.67 (d, J = 8.5 Hz, 1H), 1.37 (s, 9H) ppm. **^{13}C NMR** (126 MHz, $CDCl_3$): δ 156.1, 128.9, 128.0 (q, J_{C-F} = 5.8 Hz), 127.9 (q, J_{C-F} = 31.4 Hz), 125.4 (q, J_{C-F} = 31.2 Hz), 125.1 (q, J_{C-F} = 5.9 Hz), 123.2 (q, J_{C-F} = 275.2 Hz), 123.2 (q, J_{C-F} = 274.6 Hz), 35.3, 31.0 ppm. **^{19}F NMR** (470 MHz, $CDCl_3$): δ -60.23 (q, J_{F-F} = 12.7 Hz, 3F), -60.39 (q, J_{F-F} = 12.7 Hz, 3F) ppm. **HRMS m/z (APCI)**: calcd. for $C_{12}H_{12}F_6$ $[M-F]^-$: 251.0854; found: 251.0855.

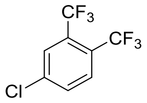

2l: 4-chloro-1,2-bis(trifluoromethyl)benzene. Prepared according to the general procedure. Reaction was run using **1l** (132.8 mg, 0.4 mmol), DDQ (181.6 mg, 0.8 mmol), DMSO (4.0 mL), $[CuCF_3]$ in DMF solution (4.0 mL, 1.6 mmol). The product was purified by flash column chromatography on silica gel (hexane) and obtained a colorless oil (50% ^{19}F NMR yield), R_f = 0.60 (hexane). The spectra contain hexane due to low boiling point. **^{1}H NMR** (500 MHz, $CDCl_3$): δ 7.84 (s, 1H), 7.80 (d, J = 8.5 Hz, 1H), 7.67 (d, J = 8.5 Hz, 1H) ppm. **^{13}C NMR** (126 MHz, $CDCl_3$): δ 138.9, 132.2, 130.0 (q, J_{C-F} = 35.8 Hz), 129.6 (m), 128.5 (m), 126.8 (q, J_{C-F} = 32.9 Hz), 122.6 (q, J_{C-F} = 271.5 Hz), 122.1 (q, J_{C-F} = 274.2 Hz) ppm. **^{19}F NMR** (470 MHz, $CDCl_3$): δ -60.26 (q, J_{F-F} = 12.7 Hz, 3F), -60.72 (q, J_{F-F} = 12.7 Hz, 3F) ppm. **HRMS m/z (APCI)**: calcd. for $C_8H_3ClF_6$ $[M]^+$: 247.9822; found: 247.9824.

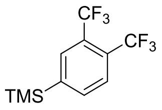

2m: (3,4-bis(trifluoromethyl)phenyl)trimethylsilane. Prepared according to the general procedure. Reaction was run using **1m** (148.0 mg, 0.4 mmol), DDQ (181.6 mg, 0.8 mmol), DMSO (8.0 mL), $[CuCF_3]$ in DMF solution (4.0 mL, 1.6 mmol). The product was purified by flash column chromatography on silica gel (hexane) and obtained a colorless oil (0.20 mmol, 57.2 mg, 50%), R_f = 0.70 (hexane). **^{1}H NMR** (500 MHz, $CDCl_3$): δ 7.94 (s, 1H), 7.81 (s, 2H), 0.33 (s, 9H) ppm. **^{13}C NMR** (126 MHz, $CDCl_3$): δ 147.0, 137.2, 132.3 (q, J_{C-F} = 5.8 Hz), 128.3 (q, J_{C-F} = 33.3 Hz), 127.2 (q, J_{C-F} = 33.4 Hz), 126.9 (q, J_{C-F} = 5.9 Hz), 123.3 (q, J_{C-F} = 275.9 Hz), 123.1 (q, J_{C-F} = 272.8 Hz), -1.42 ppm. **^{19}F NMR** (470 MHz, $CDCl_3$): δ -60.34 (q, J_{F-F} = 12.7 Hz, 3F), -60.68 (q, J_{F-F} = 12.7 Hz, 3F) ppm. **HRMS m/z (EI)**: calcd. for $C_{11}H_{12}F_6Si$ $[M-CH_3]^+$: 271.0372; found: 271.0371.

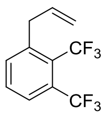

2n: 1-allyl-2,3-bis(trifluoromethyl)benzene. Prepared according to the general procedure. Reaction was run using **1n** (135.2 mg, 0.4 mmol), DDQ (181.6 mg, 0.8 mmol), DMSO (8.0 mL), [$CuCF_3$] in DMF solution (4.0 mL, 1.6 mmol). The product was purified by flash column chromatography on silica gel (hexane) and obtained a colorless oil (0.26 mmol, 66.0 mg, 65%), R_f = 0.60 (hexane). **^{1}H NMR** (500 MHz, $CDCl_3$): δ 7.74 (s, 1H), 7.60-7.55 (m, 2H), 5.97-5.90 (m, 1H), 5.15-5.07 (m, 2H), 3.63 (d, J = 5.5 Hz, 1H) ppm. **^{13}C NMR** (126 MHz, $CDCl_3$): δ 142.0 (q, J_{C-F} = 1.9 Hz), 136.2, 135.8, 131.4, 128.9 (q, J_{C-F} = 32.4 Hz), 127.1 (q, J_{C-F} = 32.4 Hz), 126.0 (q, J_{C-F} = 7.1 Hz), 123.5 (q, J_{C-F} = 276.2 Hz), 123.3 (q, J_{C-F} = 274.6 Hz), 117.3, 38.4 (q, J_{C-F} = 3.7 Hz) ppm. **^{19}F NMR** (470 MHz, $CDCl_3$): δ -54.93 (q, J_{F-F} = 16.0 Hz, 3F), -58.65 (q, J_{F-F} = 16.0 Hz, 3F) ppm. **HRMS m/z (APCI)**: calcd. for $C_{11}H_8F_6$ $[M]^+$: 254.0525; found: 254.0526.

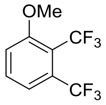

2o: 1-methoxy-2,3-bis(trifluoromethyl)benzene. Prepared according to the general procedure. Reaction was run using **1o** (131.2 mg, 0.4 mmol), DDQ (181.6 mg, 0.8 mmol), DMSO (4.0 mL), [$CuCF_3$] in DMF solution (4.0 mL, 1.6 mmol). The product was purified by flash column chromatography on silica gel (hexane) and obtained a colorless oil (36% ^{19}F NMR yield), R_f = 0.40 (hexane). **^{1}H NMR** (500 MHz, $CDCl_3$): δ 7.59 (t, J = 8.0 Hz, 1H), 7.43 (d, J = 8.0 Hz, 1H), 7.27 (d, J = 7.5 Hz, 1H), 3.95 (s, 3H) ppm. **^{13}C NMR** (126 MHz, $CDCl_3$): δ 159.6, 132.9, 129.7 (q, J_{C-F} = 34.3 Hz), 123.0 (q, J_{C-F} = 274.2 Hz), 122.7 (q, J_{C-F} = 274.8 Hz), 119.3 (m), 117.3 (q, J_{C-F} = 32.6 Hz), 116.7, 56.9 (q, J_{C-F} = 18.6 Hz) ppm. **^{19}F NMR** (470 MHz, $CDCl_3$): δ -58.07 (q, J_{F-F} = 16.0 Hz, 3F), -58.99 (q, J_{F-F} = 16.0 Hz, 3F) ppm. **HRMS m/z (APCI)**: calcd. for $C_9H_9F_6O$ $[M]^+$: 244.0317; found: 244.0319.

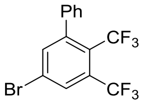

2p: 5-bromo-2,3-bis(trifluoromethyl)-1,1'-biphenyl. Prepared according to the general procedure. Reaction was run using **1p** (184.4 mg, 0.4 mmol), DDQ (181.6 mg, 0.8 mmol), DMSO (4.0 mL), [$CuCF_3$] in DMF solution (4.0 mL, 1.6 mmol). The product was purified by flash column chromatography on silica gel (hexane) and obtained a colorless oil (0.14 mmol, 51.5 mg, 35%), R_f = 0.40 (hexane). **^{1}H NMR** (500 MHz, $CDCl_3$): δ 8.03 (s, 1H), 7.73 (s, 1H), 7.44 (m, 3H), 7.30 (m, 2H) ppm. **^{13}C NMR** (126 MHz, $CDCl_3$): δ 146.1, 139.3, 139.0, 138.8, 131.0 (q, J_{C-F} = 33.5 Hz), 130.3 (dq, J_{C-F} = 25.6 Hz, J_{C-F} = 6.8 Hz), 128.3 (m), 125.9 (q, J_{C-F} = 32.5 Hz), 125.6, 122.9 (q, J_{C-F} = 276.8 Hz), 122.5 (q, J_{C-F} = 275.3 Hz) (one carbon missing due to overlap) ppm. **^{19}F NMR** (470 MHz, $CDCl_3$): δ -52.41 (q, J_{F-F} = 15.0 Hz, 3F), -59.05 (q, J_{F-F} = 15.5 Hz, 3F) ppm. **HRMS m/z (APCI)**: calcd. for $C_{14}H_7BrF_6$ $[M]^+$: 367.9630; found: 367.9629.

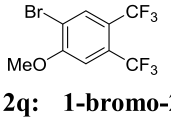

2q: 1-bromo-2-methoxy-4,5-bis(trifluoromethyl)benzene. Prepared according to the general

procedure. Reaction was run using **1q** (162.4 mg, 0.4 mmol), DDQ (181.6 mg, 0.8 mmol), DMSO (8.0 mL), [$CuCF_3$] in DMF solution (4.0 mL, 1.6 mmol). The product was purified by flash column chromatography on silica gel (hexane) and obtained a colorless oil (0.17 mmol, 55.4 mg, 43%), R_f = 0.50 (hexane : CH_2Cl_2 = 8 : 1). **^{1}H NMR** (500 MHz, $CDCl_3$): δ 7.74 (s, 1H), 7.00 (s, 1H), 3.75 (s, 3H) ppm. **^{13}C NMR** (126 MHz, $CDCl_3$): δ 158.5, 133.3 (dq, $J_{C\text{-}F}$ = 26.2 Hz, $J_{C\text{-}F}$ = 6.0 Hz), 129.0 (q, $J_{C\text{-}F}$ = 34.5 Hz), 122.5 (q, $J_{C\text{-}F}$ = 274.4 Hz), 122.3 (q, $J_{C\text{-}F}$ = 273.5 Hz), 121.3 (q, $J_{C\text{-}F}$ = 34.7 Hz), 115.4, 110.9 (m), 56.9 (q, $J_{C\text{-}F}$ = 39.2 Hz) ppm. **^{19}F NMR** (470 MHz, $CDCl_3$): δ -59.41 (m, 3F), -60.42 (m, 3F) ppm. **HRMS m/z (APCI)**: calcd. for $C_9H_5BrF_6O$ [M]$^+$: 321.9423; found: 321.9425.

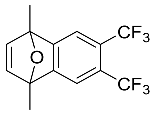

2s: 1,4-dimethyl-6,7-bis(trifluoromethyl)-1,4-dihydro-1,4-epoxynaphthalene. Prepared according to the general procedure. Reaction was run using **1s** (160.4 mg, 0.4 mmol), DDQ (181.6 mg, 0.8 mmol), DMSO (8.0 mL), [$CuCF_3$] in DMF solution (4.0 mL, 1.6 mmol). The product was purified by flash column chromatography on silica gel (hexane) and obtained a colorless oil (0.28 mmol, 86.2 mg, 70%), R_f = 0.50 (hexane : EtOAc = 8 : 1). **^{1}H NMR** (400 MHz, $CDCl_3$): δ 7.53 (s, 2H), 6.82 (s, 2H), 1.94 (s, 6H) ppm. **^{13}C NMR** (101 MHz, $CDCl_3$): δ 158.0, 146.8, 126.0 (m), 123.0 (q, $J_{C\text{-}F}$ = 276.0 Hz), 117.4 (m), 88.9, 15.0 ppm. **^{19}F NMR** (376 MHz, $CDCl_3$): δ -59.46 (s, 6F) ppm. **HRMS m/z (APCI)**: calcd. for $C_{14}H_{10}F_6O$ [M]$^+$: 309.0709; found: 309.0708.

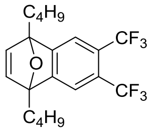

2t: 1,4-dibutyl-6,7-bis(trifluoromethyl)-1,4-dihydro-1,4-epoxynaphthalene. Prepared according to the general procedure. Reaction was run using **1t** (190.4 mg, 0.4 mmol), DDQ (181.6 mg, 0.8 mmol), DMSO (12.0 mL), [$CuCF_3$] in DMF solution (4.0 mL, 1.6 mmol). The product was purified by flash column chromatography on silica gel (hexane) and obtained a colorless oil (0.25 mmol, 97.2 mg, 62%), R_f = 0.60 (hexane : CH_2Cl_2 = 6 : 1). **^{1}H NMR** (500 MHz, $CDCl_3$): δ 7.52 (s, 2H), 6.82 (s, 2H), 2.40-2.36 (m, 2H), 2.28-2.21 (m, 2H), 1.67-1.55 (m, 4H), 1.53-1.46 (m, 4H), 1.00 (d, J = 7.0 Hz, 6H) ppm. **^{13}C NMR** (126 MHz, $CDCl_3$): δ 158.1, 146.0, 125.7 (m), 123.1 (q, $J_{C\text{-}F}$ = 275.6 Hz), 117.7, 92.2, 28.8, 26.9, 23.2, 14.1 ppm. **^{19}F NMR** (470 MHz, $CDCl_3$): δ -59.43 (s, 6F) ppm. **HRMS m/z (ESI)**: calcd. for $C_{20}H_{22}F_6O$ [M+H]$^+$: 393.1648; found: 393.1647.

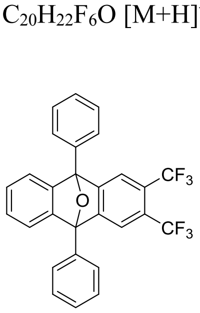

2u: 9,10-diphenyl-2,3-bis(trifluoromethyl)-9,10-dihydro-9,10-epoxyanthracene. Prepared according to the general procedure. Reaction was run using **1u** (226.4 mg, 0.4 mmol), DDQ (181.6 mg, 0.8 mmol), DMSO (8.0 mL), [$CuCF_3$] in DMF solution (4.0 mL, 1.6 mmol). The product was purified by flash

column chromatography on silica gel (hexane) and obtained a colorless oil (0.25 mmol, 121.4 mg, 63%), R_f = 0.60 (hexane : CH_2Cl_2 = 3 : 1). **^{1}H NMR** (400 MHz, $CDCl_3$): δ 8.01 (d, J = 7.6 Hz, 4H), 7.88 (s, 2H), 7.72 (d, J = 7.6 Hz, 4H), 7.61 (d, J = 7.2 Hz, 2H), 7.55-7.51 (m, 2H), 7.19-7.17 (m, 2H) ppm. **^{13}C NMR** (101 MHz, $CDCl_3$): δ 155.6, 148.9, 133.6, 129.3, 129.1, 127.9 (m), 126.8, 126.7, 122.9 (q, J_{C-F} = 276.3 Hz), 121.4, 119.6 (m), 90.7 ppm. **^{19}F NMR** (376 MHz, $CDCl_3$): δ -59.73 (s, 6F) ppm. **HRMS m/z (ESI)**: calcd. for $C_{28}H_{16}F_6O$ $[M+H]^+$: 483.1178; found: 483.1179.

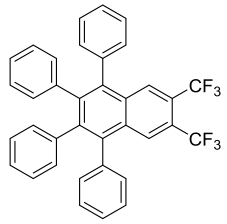

2v: 1,2,3,4-tetraphenyl-6,7-bis(trifluoromethyl)naphthalene. Prepared according to the general procedure. Reaction was run using **1v** (260.8 mg, 0.4 mmol), DDQ (181.6 mg, 0.8 mmol), DMSO (8.0 mL), $[CuCF_3]$ in DMF solution (4.0 mL, 1.6 mmol). The product was purified by flash column chromatography on silica gel (hexane) and obtained a colorless oil (0.19 mmol, 109.0 mg, 48%), R_f = 0.50 (hexane : CH_2Cl_2 = 5 : 1). **^{1}H NMR** (500 MHz, $CDCl_3$): δ 8.28 (s, 2H), 7.38-7.29 (m, 6H), 7.27 (d, J = 7.0 Hz, 4H), 6.99-6.94 (m, 6H), 6.92 (d, J = 6.0 Hz, 4H) ppm. **^{13}C NMR** (126 MHz, $CDCl_3$): δ 142.8, 139.7, 139.5, 137.8, 132.1, 131.1, 131.0, 128.5, 128.1, 127.5, 127.0, 126.1, 123.7 (m), 123.3 (q, J_{C-F} = 275.2 Hz) ppm. **^{19}F NMR** (470 MHz, $CDCl_3$): δ -60.43 (s, 6F) ppm. **HRMS m/z (ESI)**: calcd. for $C_{36}H_{22}F_6$ $[M+H]^+$: 569.1699; found: 569.1697.

Spectra

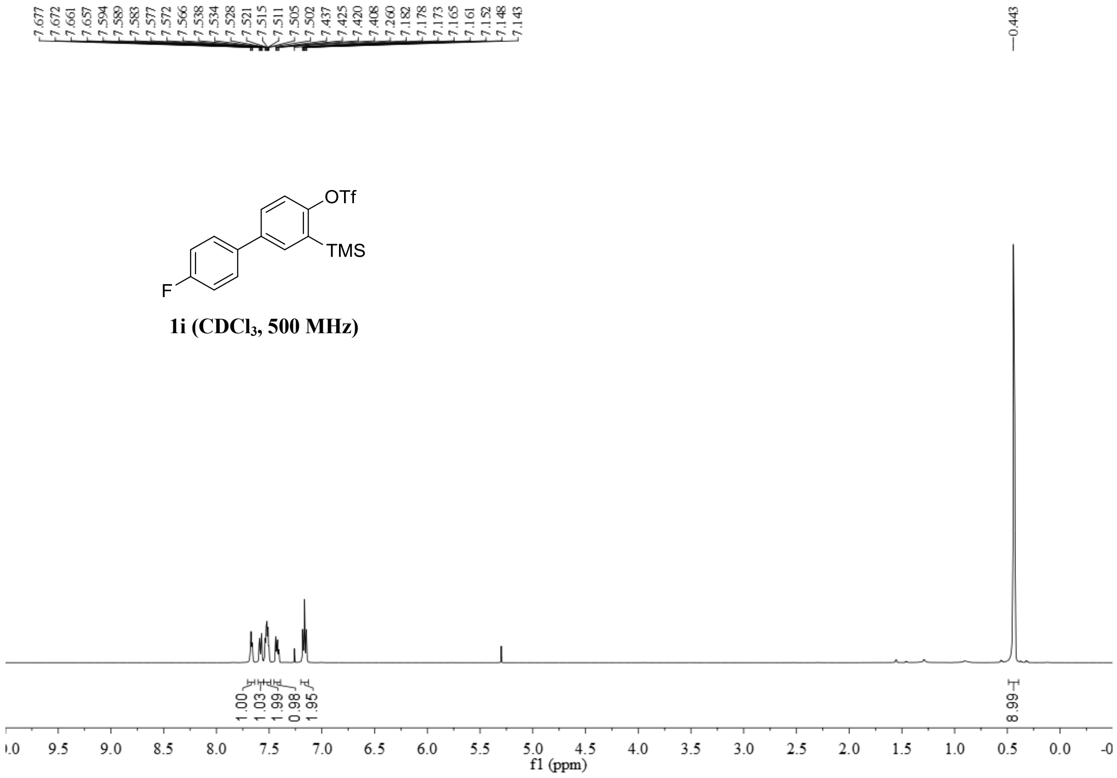
OTf
TMS
F
1i (CDCl3, 500 MHz)
f1 (ppm)

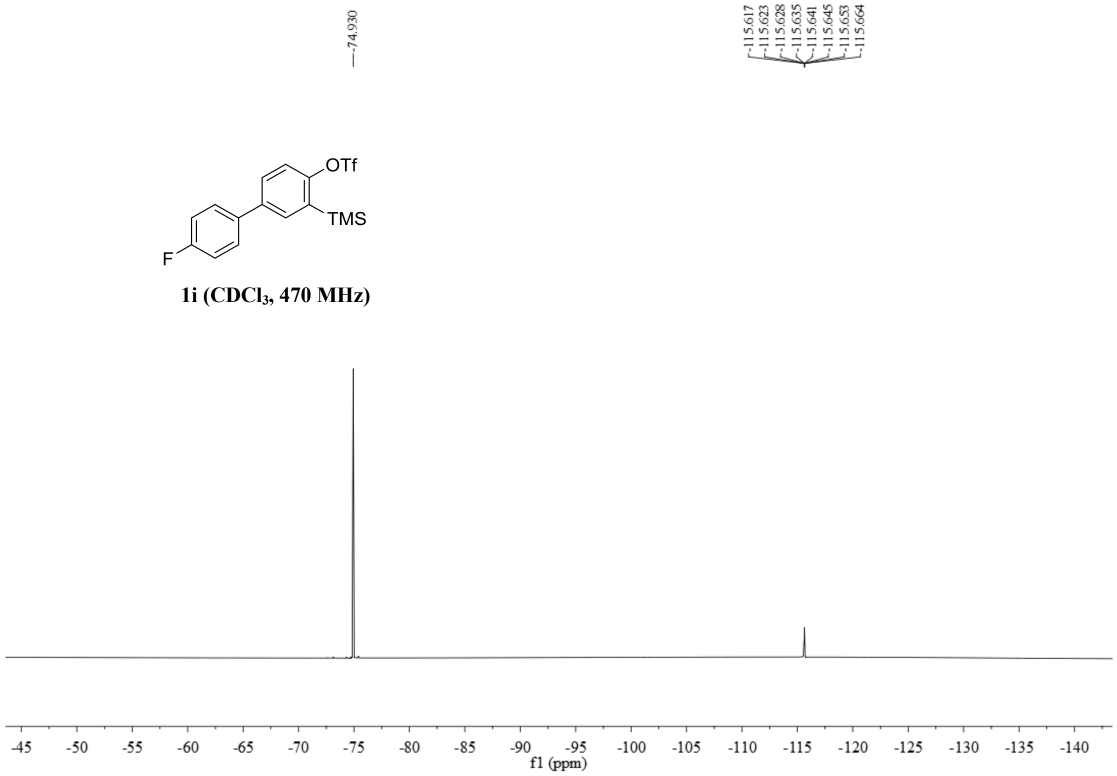
OTf
TMS
F
1i (CDCl3, 470 MHz)
f1 (ppm)

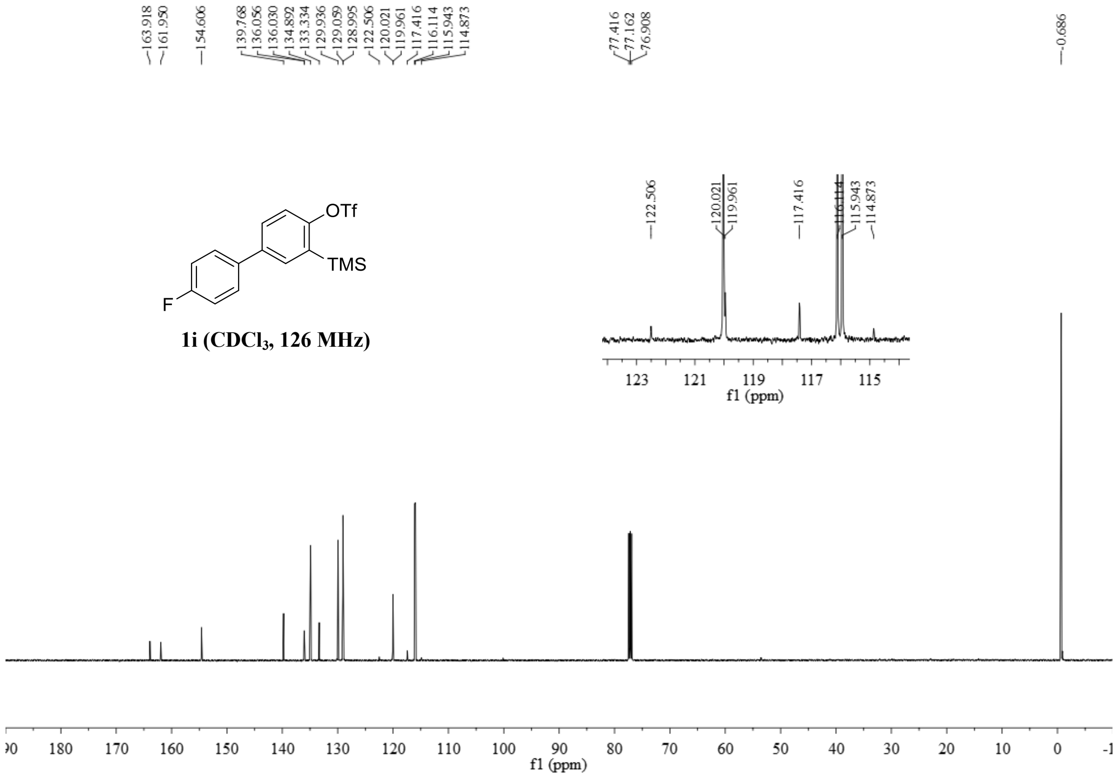

1i ($CDCl_3$, 126 MHz)

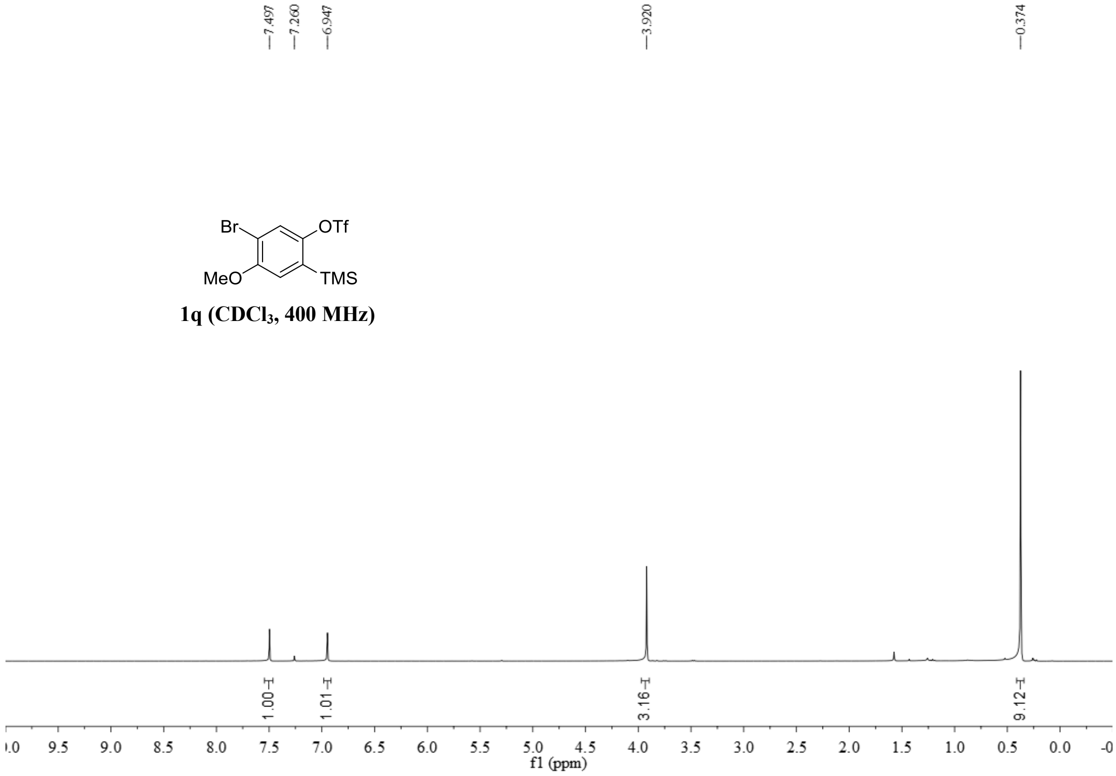

1q ($CDCl_3$, 400 MHz)

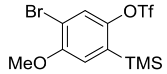

1q (CDCl$_3$, 376 MHz)

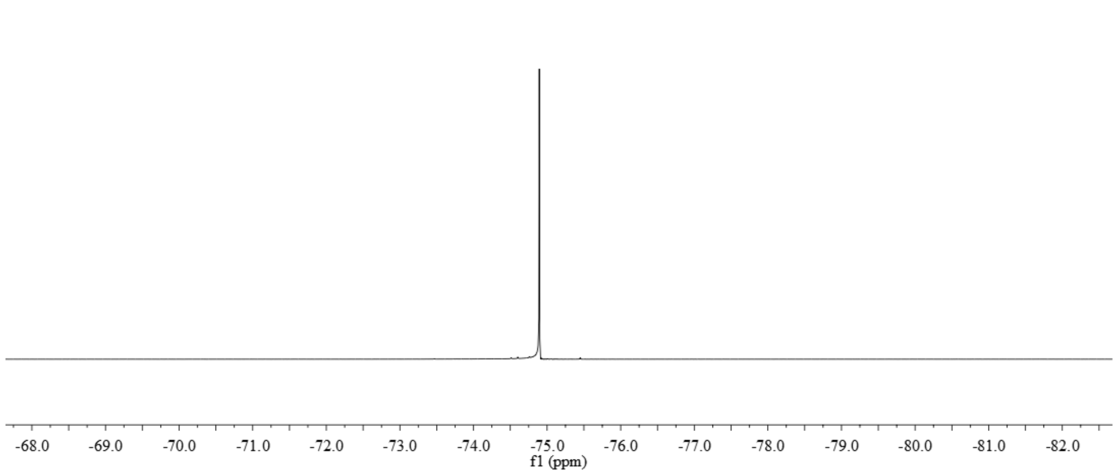

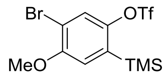

1q (CDCl$_3$, 101 MHz)

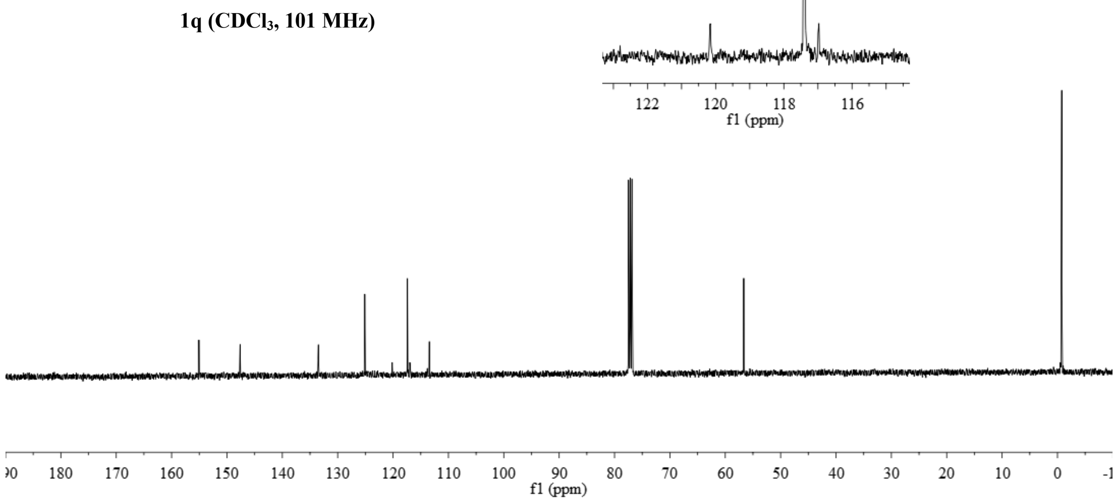

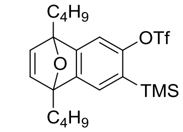

1t ($CDCl_3$, 500 MHz)

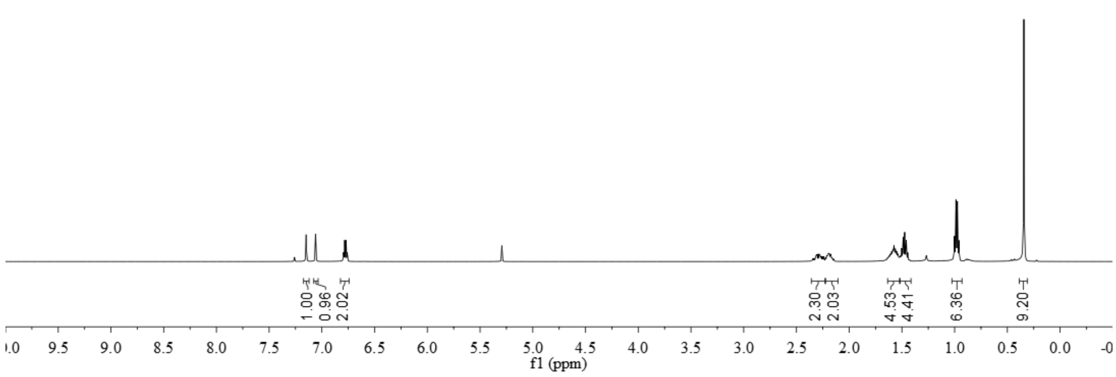

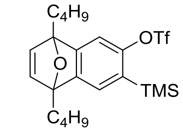

1t ($CDCl_3$, 470 MHz)

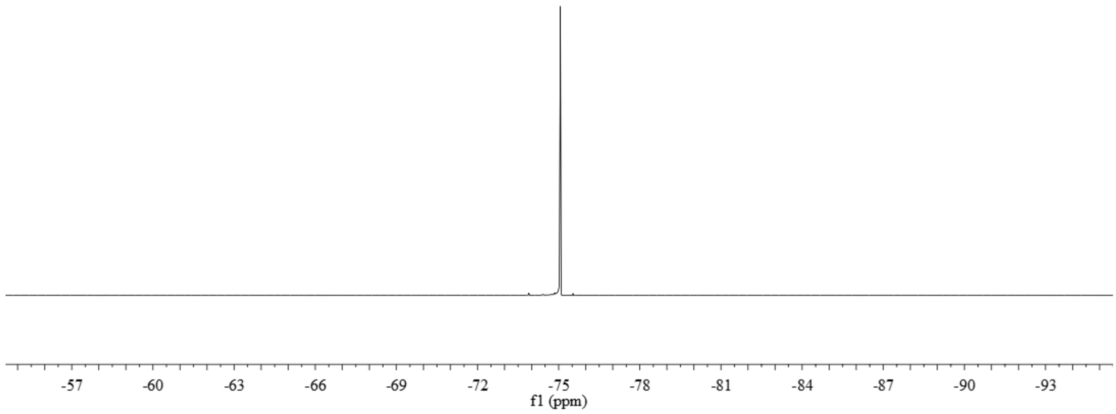

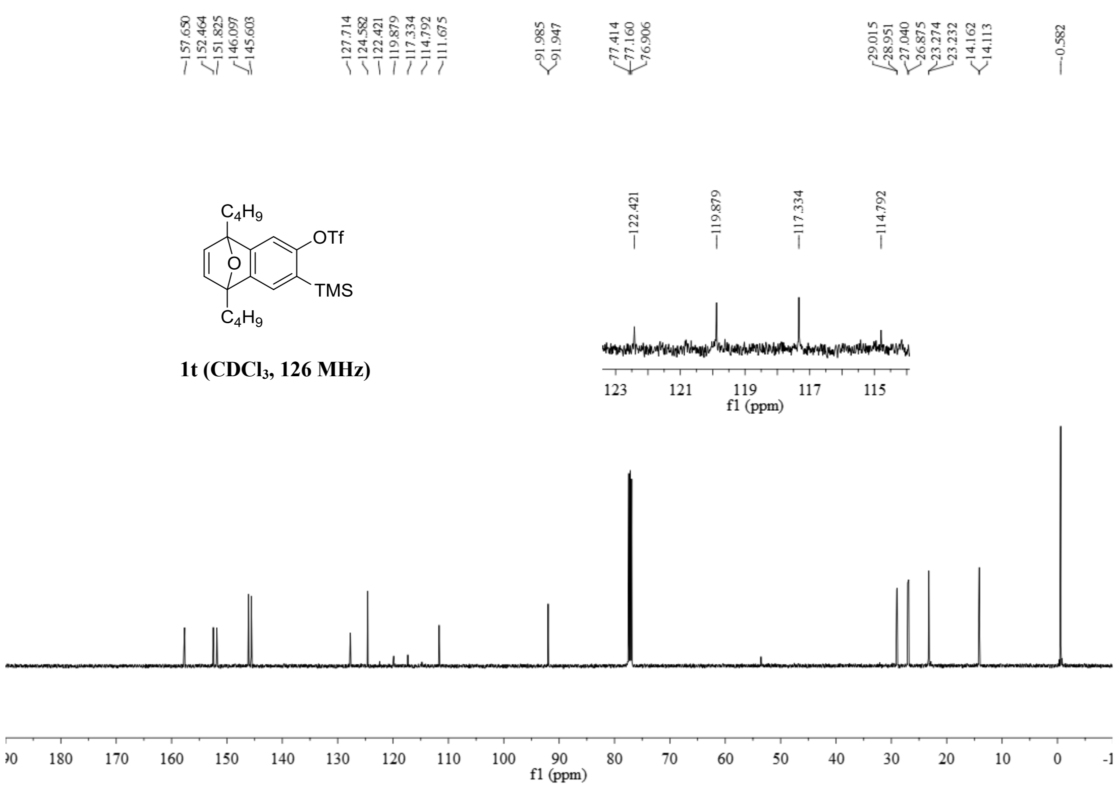

1t ($CDCl_3$, 126 MHz)

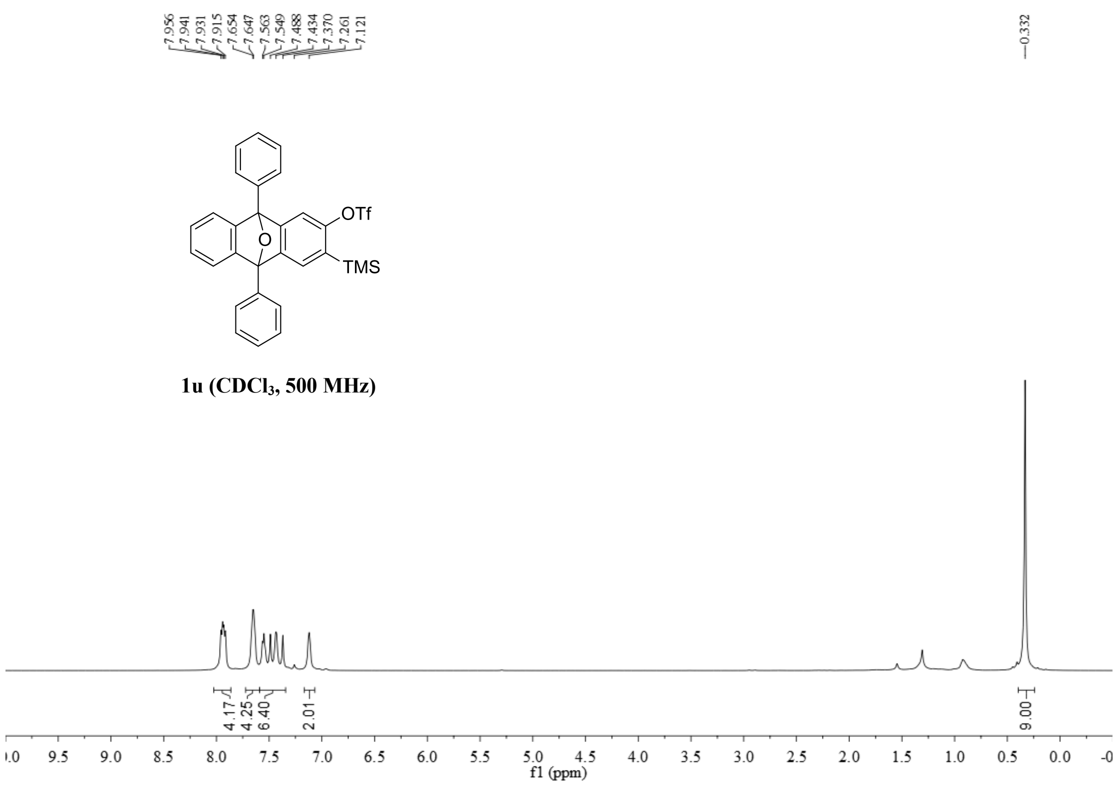

1u ($CDCl_3$, 500 MHz)

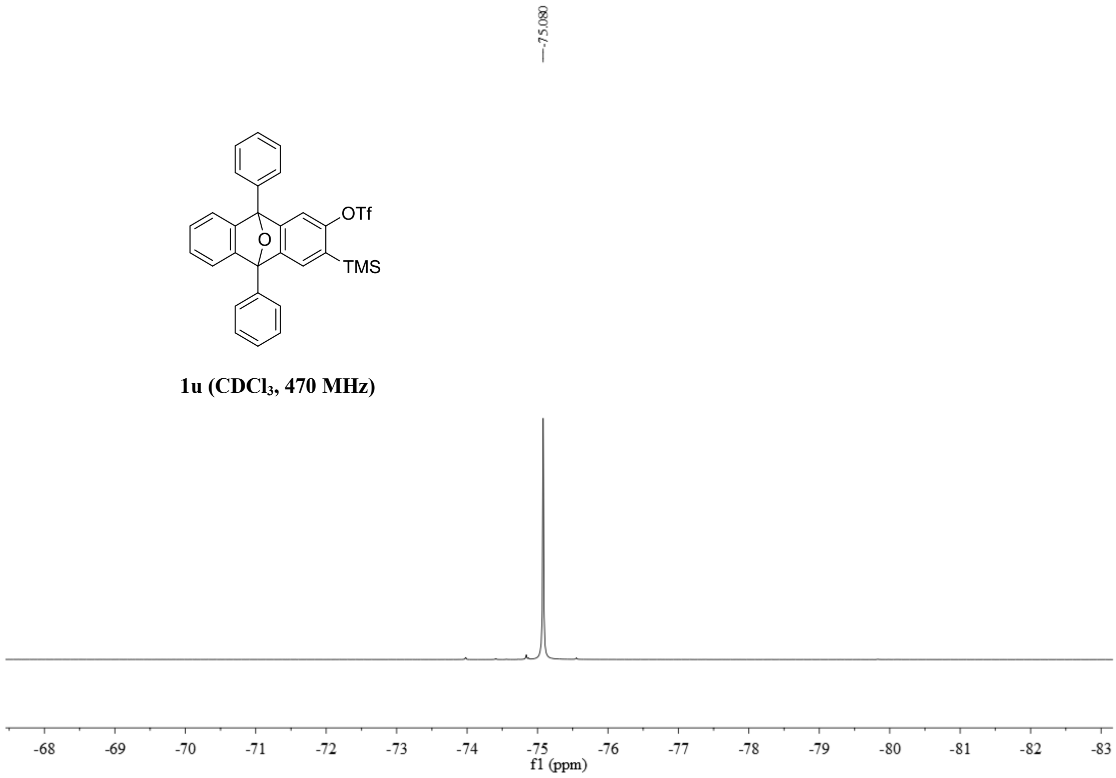

1u (CDCl$_3$, 470 MHz)

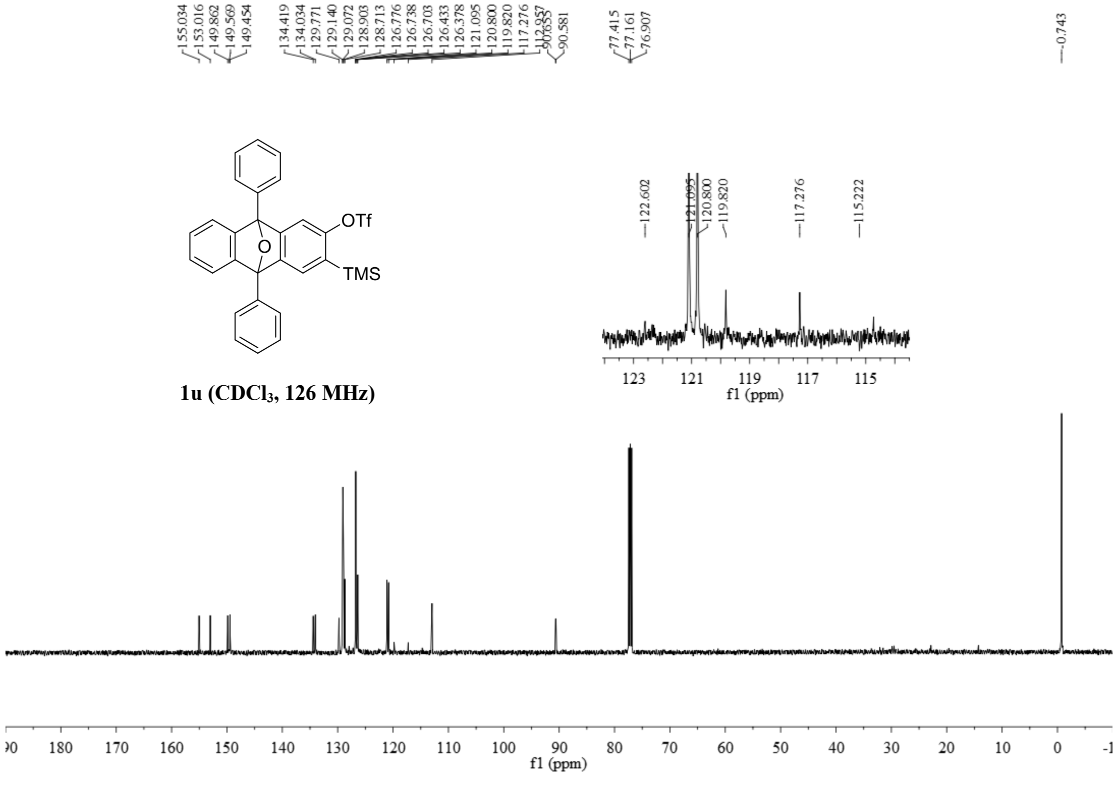

1u (CDCl$_3$, 126 MHz)

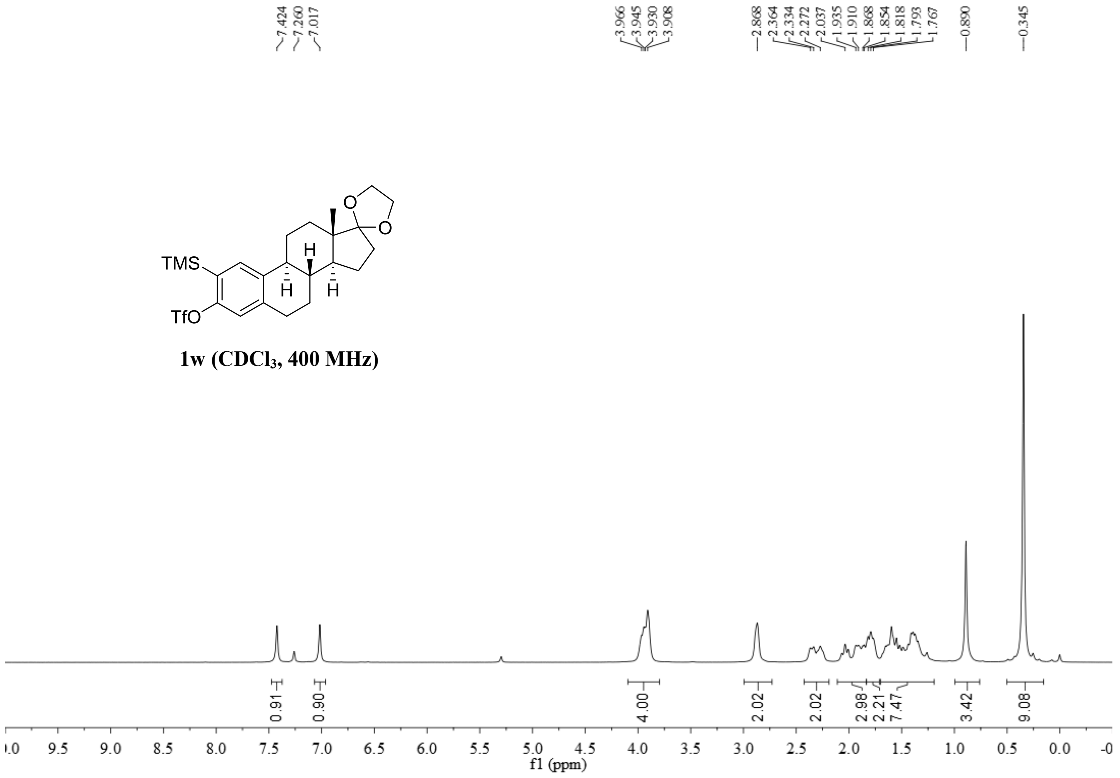

1w ($CDCl_3$, 400 MHz)

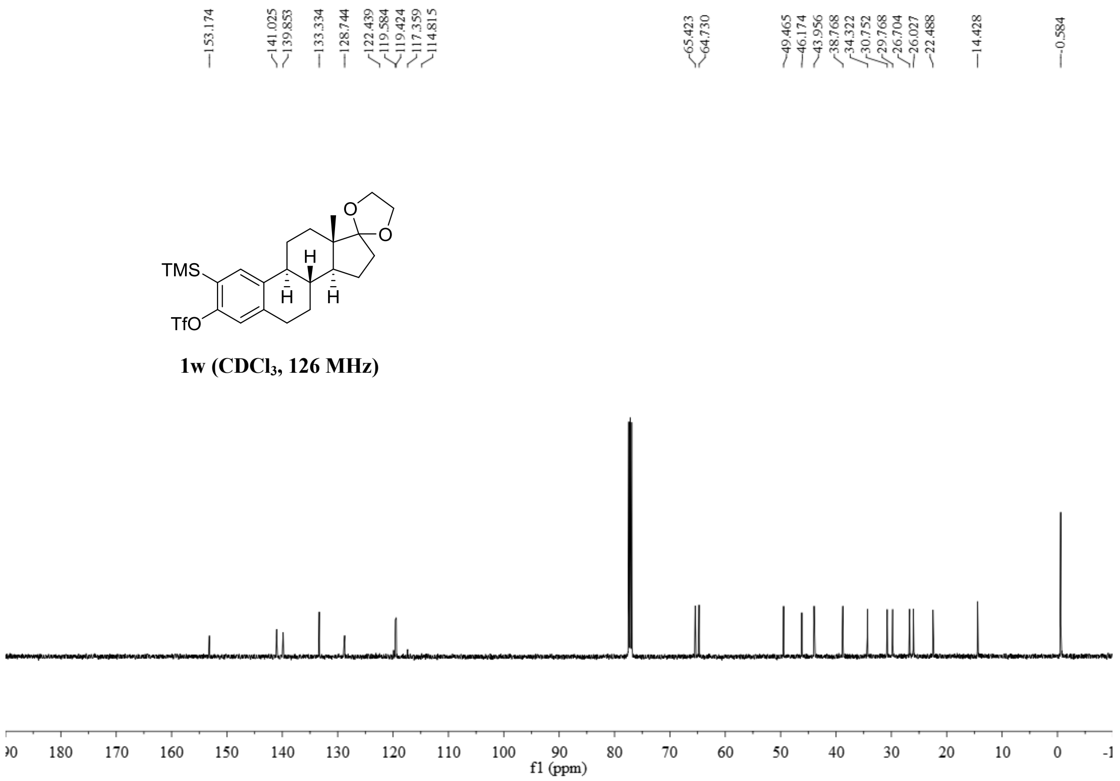

1w ($CDCl_3$, 126 MHz)

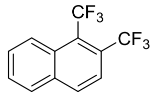

2a (CDCl$_3$, 400 MHz)

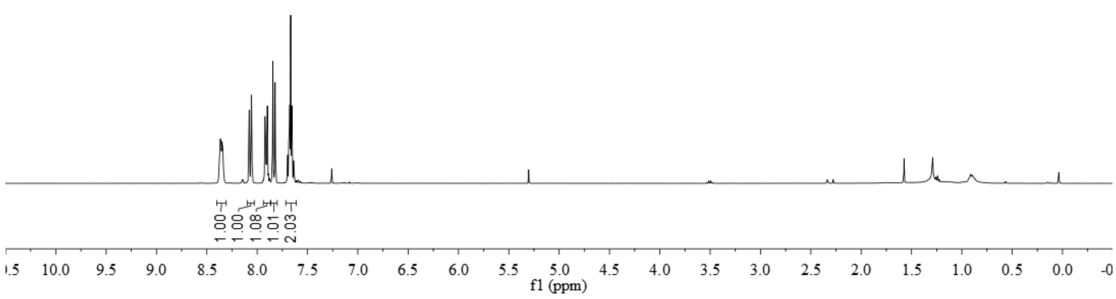

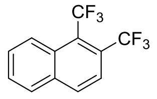

2a (CDCl$_3$, 470 MHz)

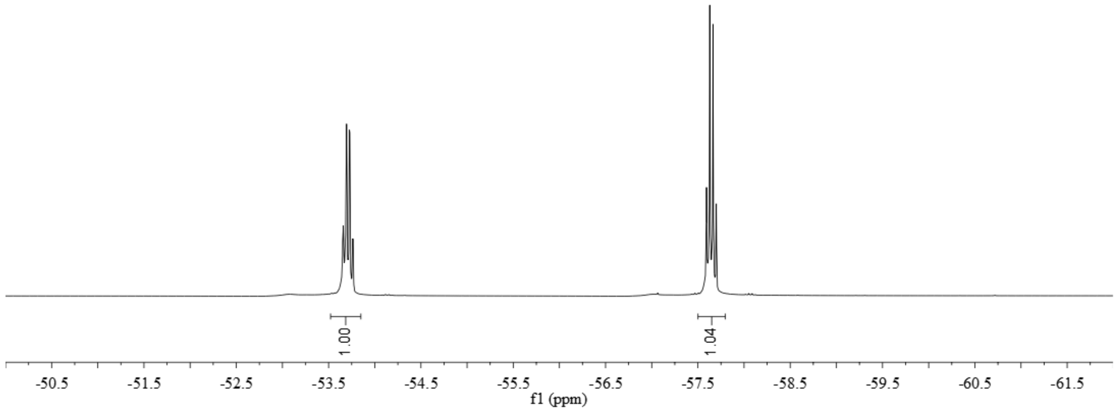

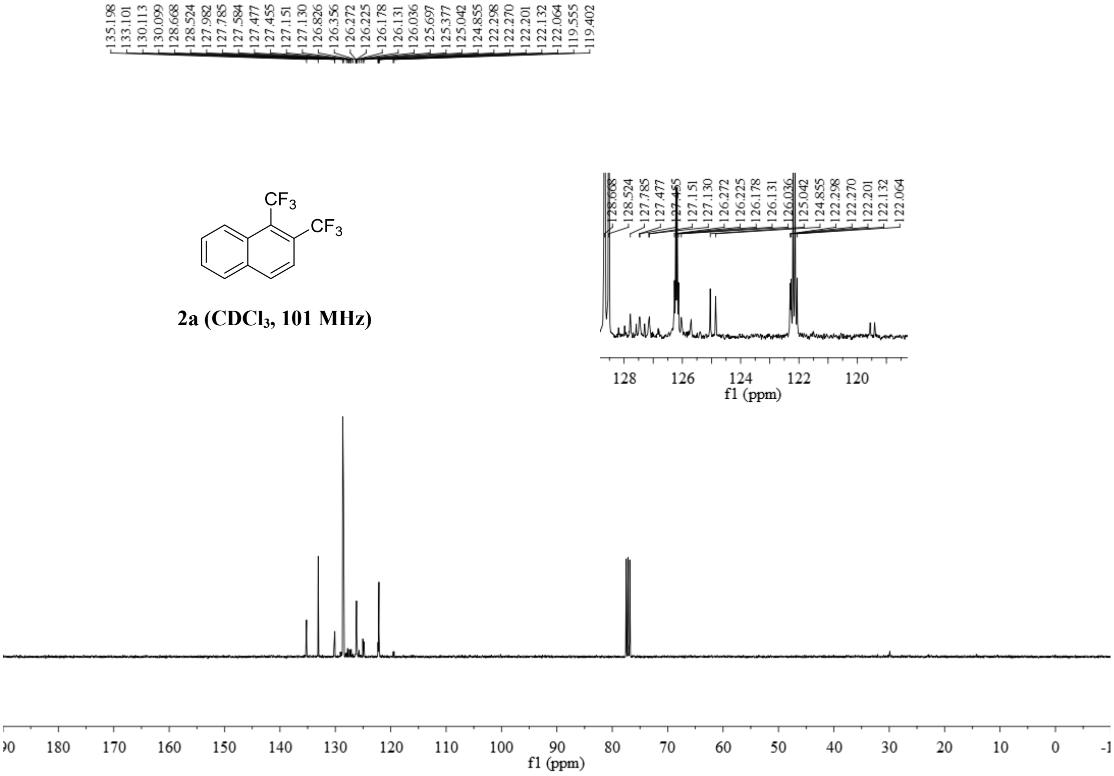

2a (CDCl$_3$, 101 MHz)

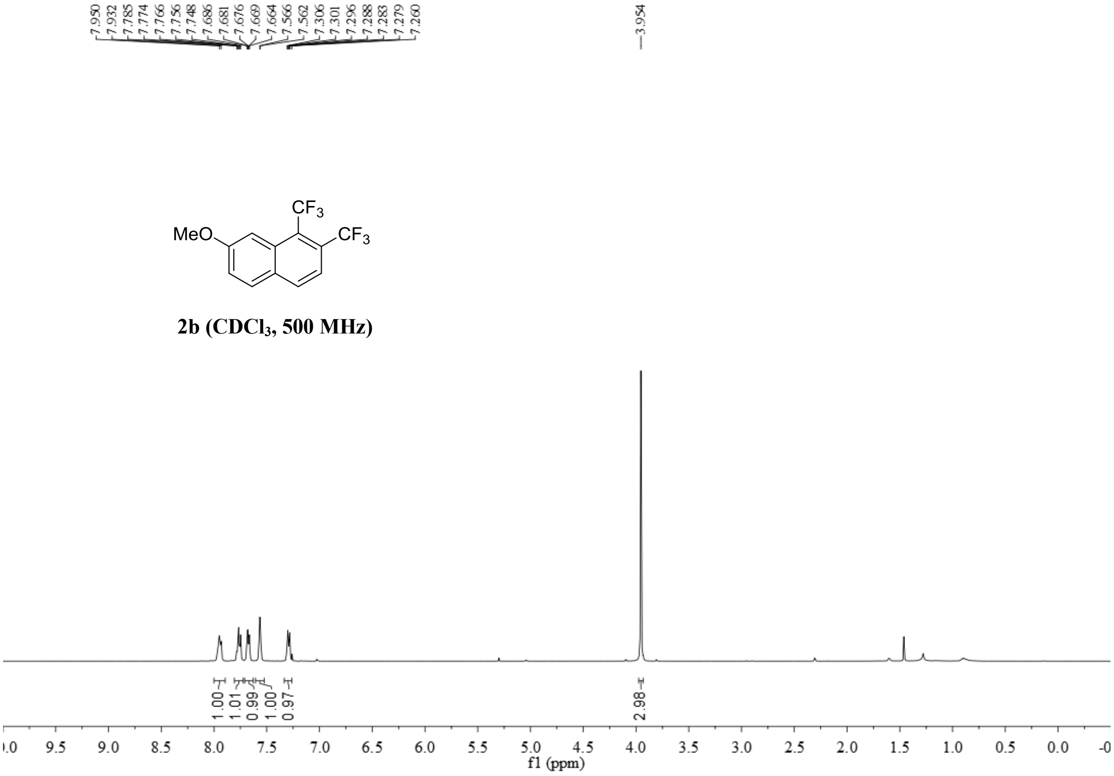

2b (CDCl$_3$, 500 MHz)

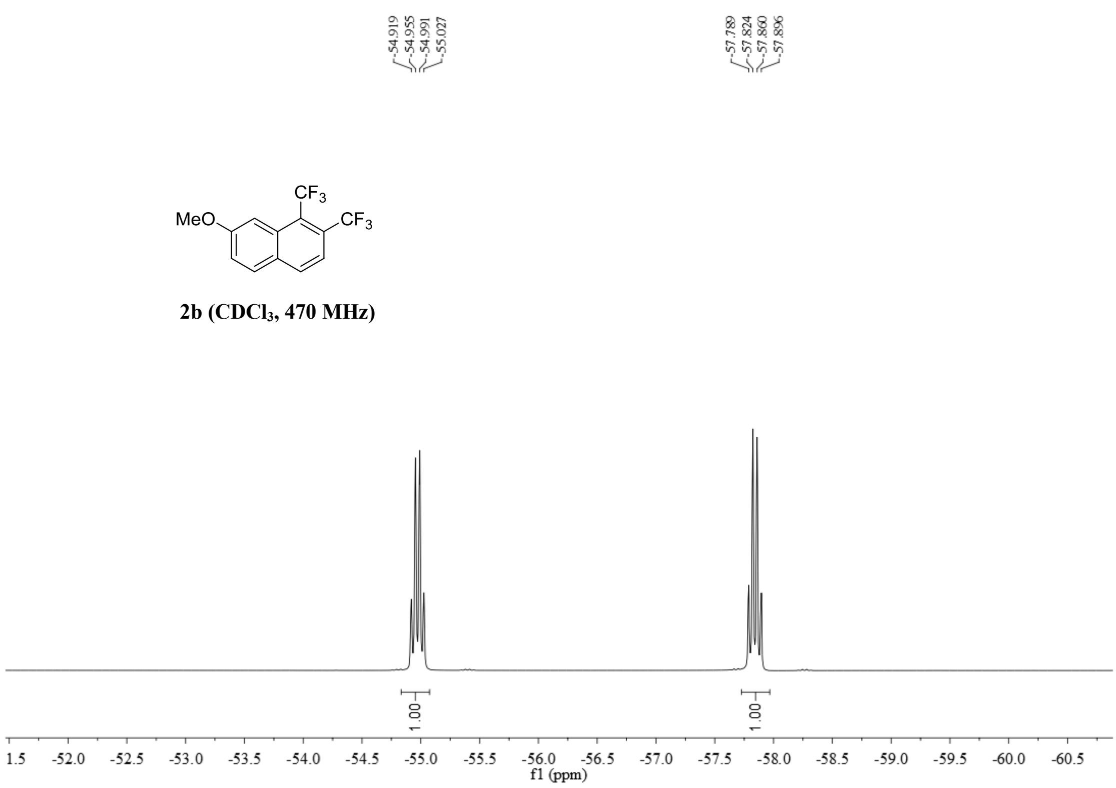

2b ($CDCl_3$, 470 MHz)

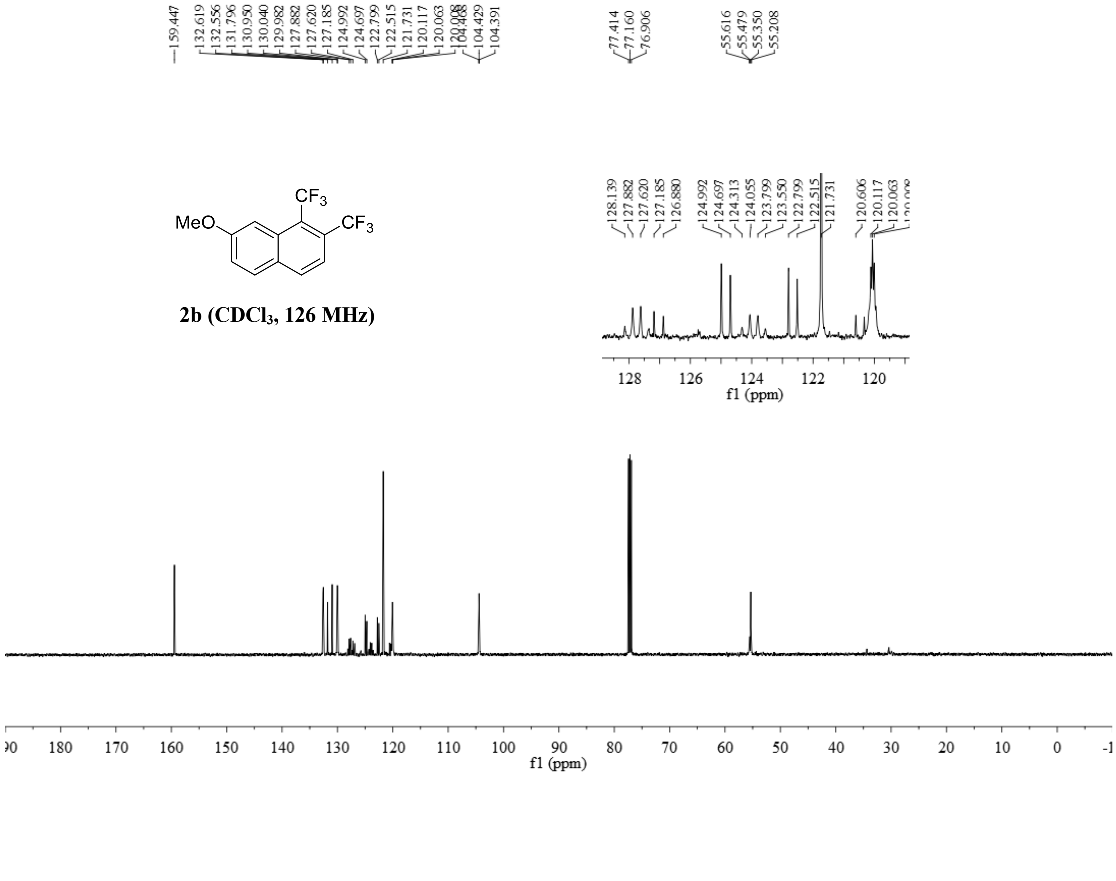

2b ($CDCl_3$, 126 MHz)

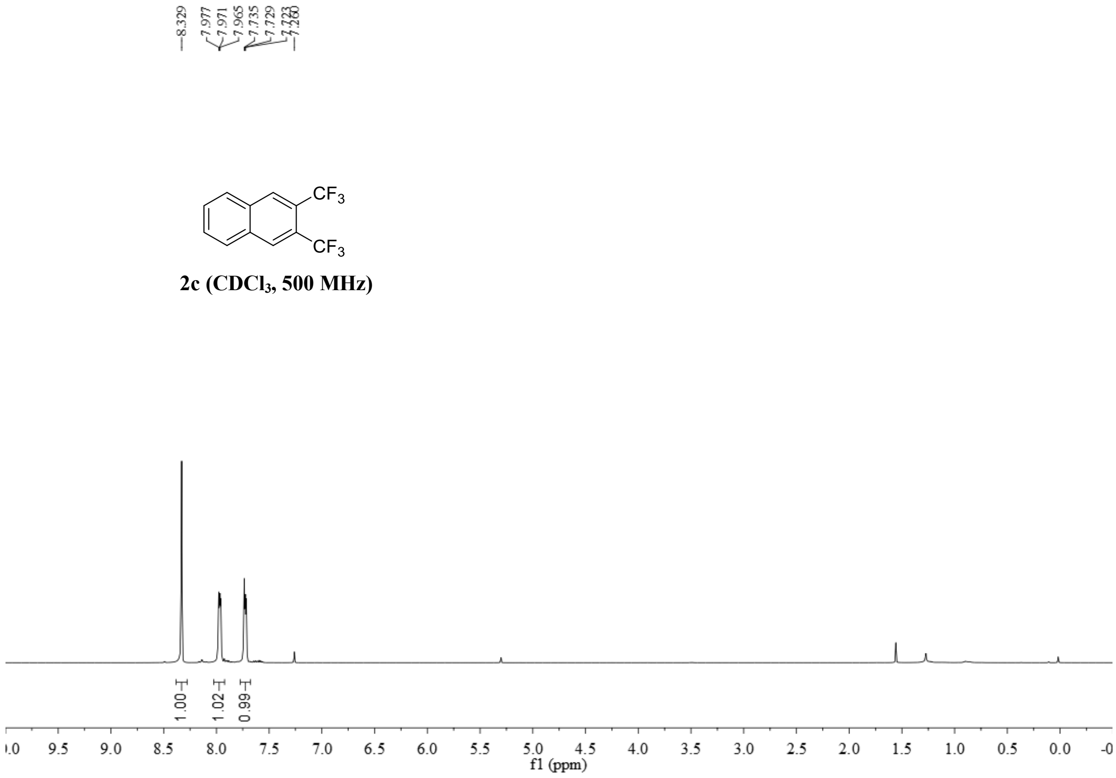

2c ($CDCl_3$, 500 MHz)

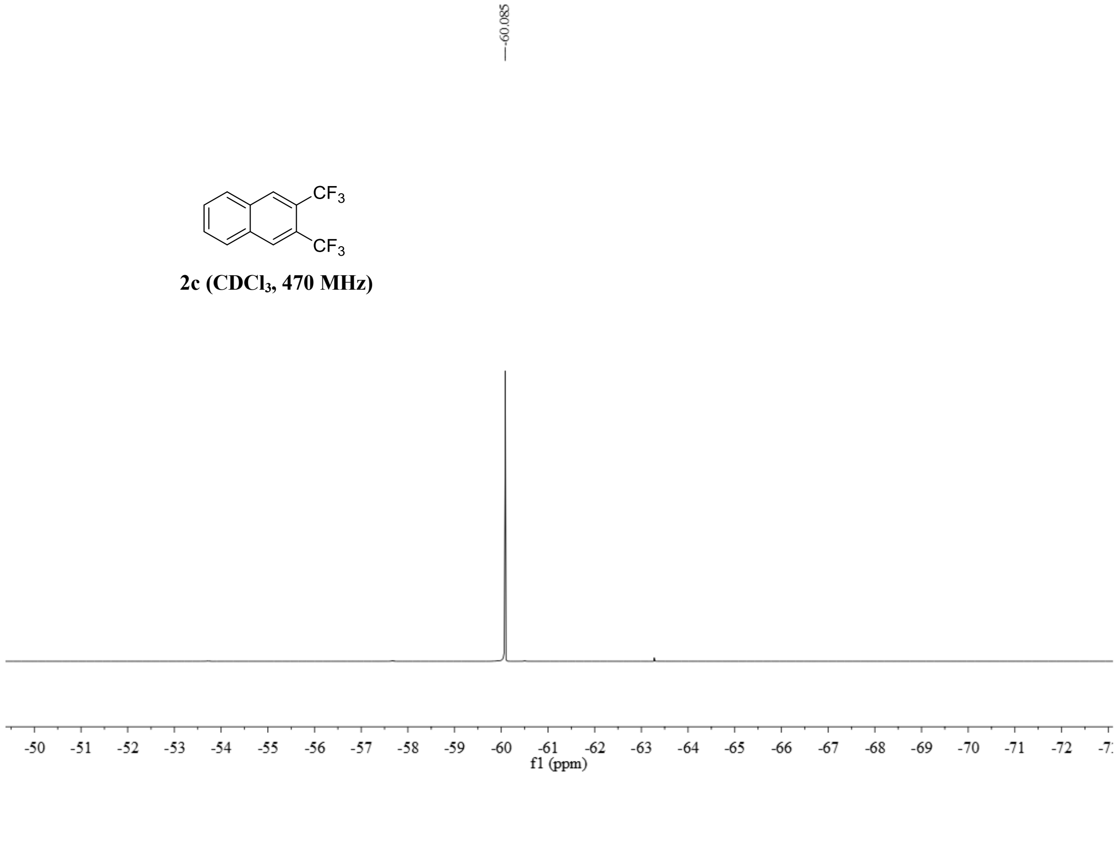

2c ($CDCl_3$, 470 MHz)

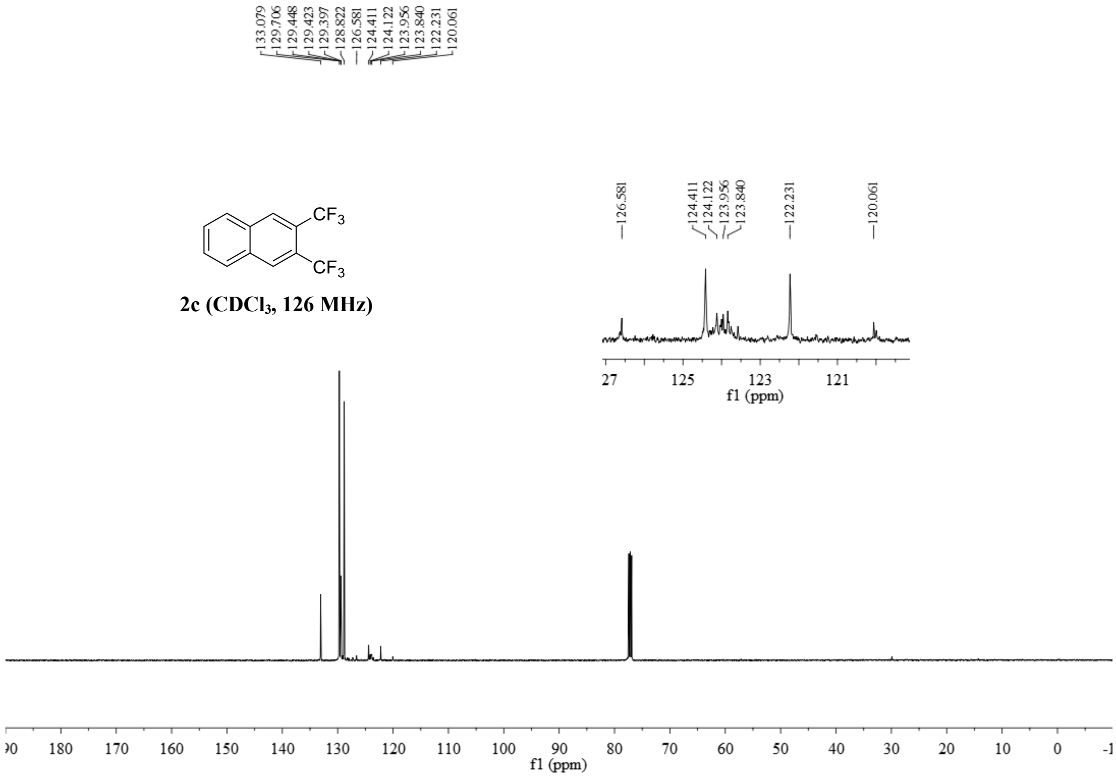

2c (CDCl$_3$, 126 MHz)

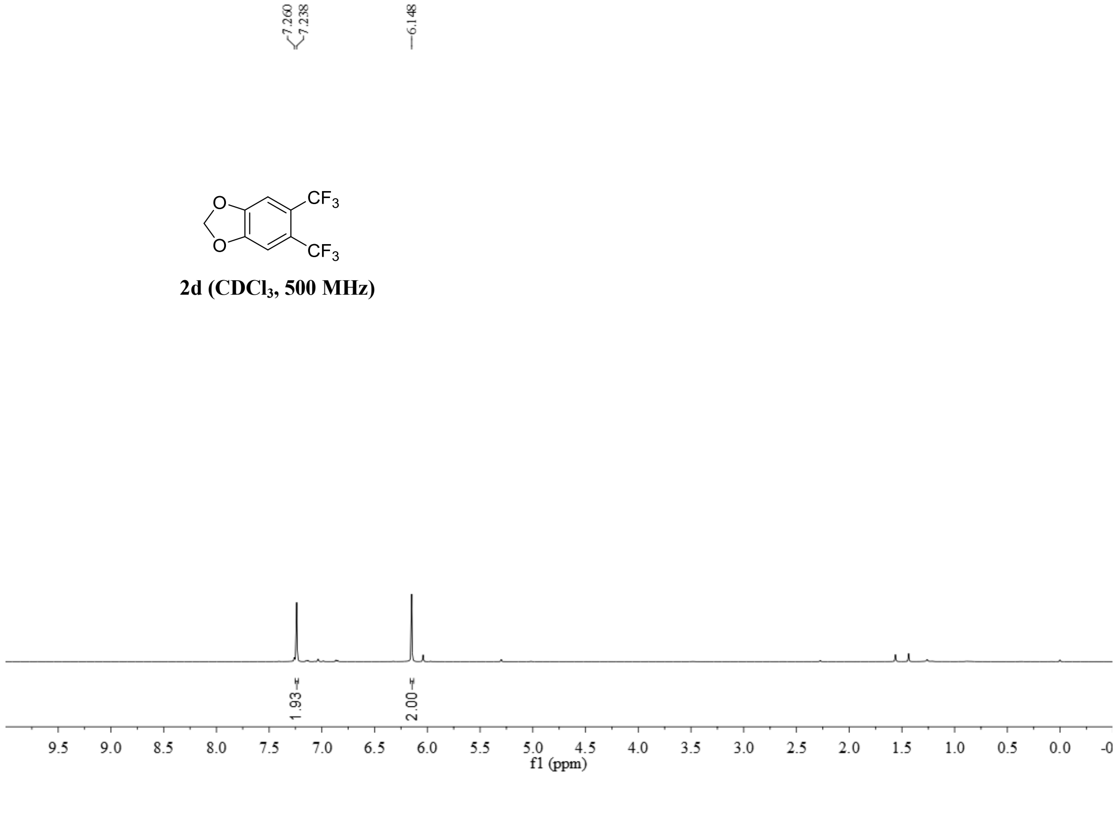

2d (CDCl$_3$, 500 MHz)

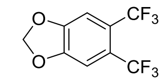

2d (CDCl$_3$, 470 MHz)

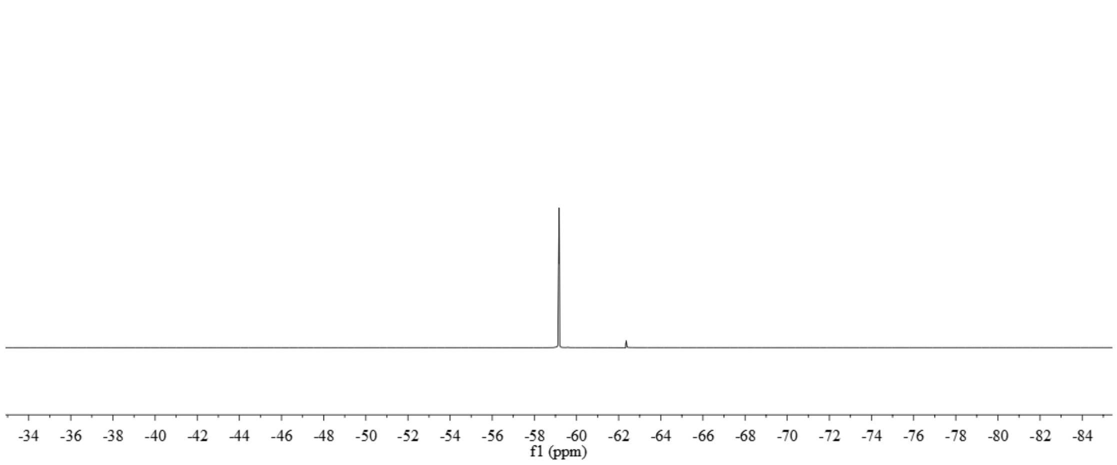

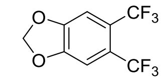

2d (CDCl$_3$, 126 MHz)

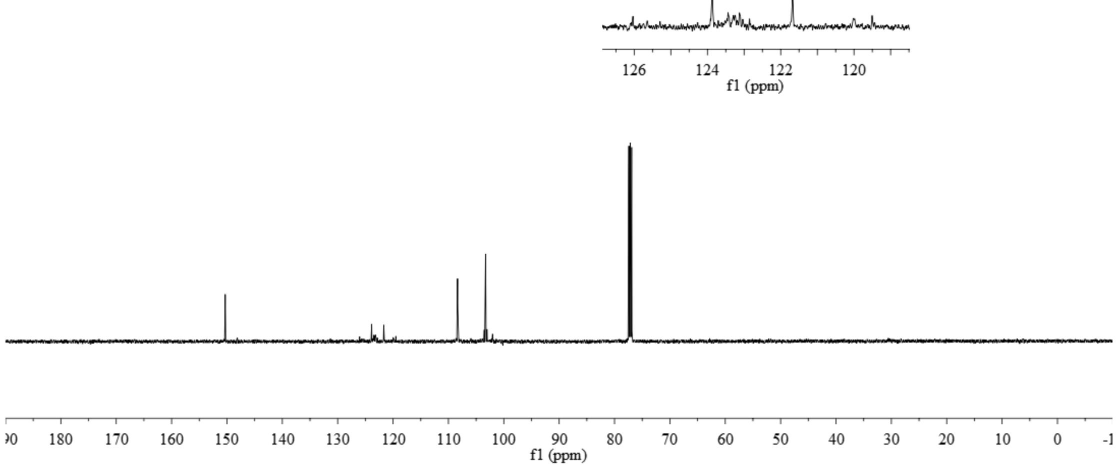

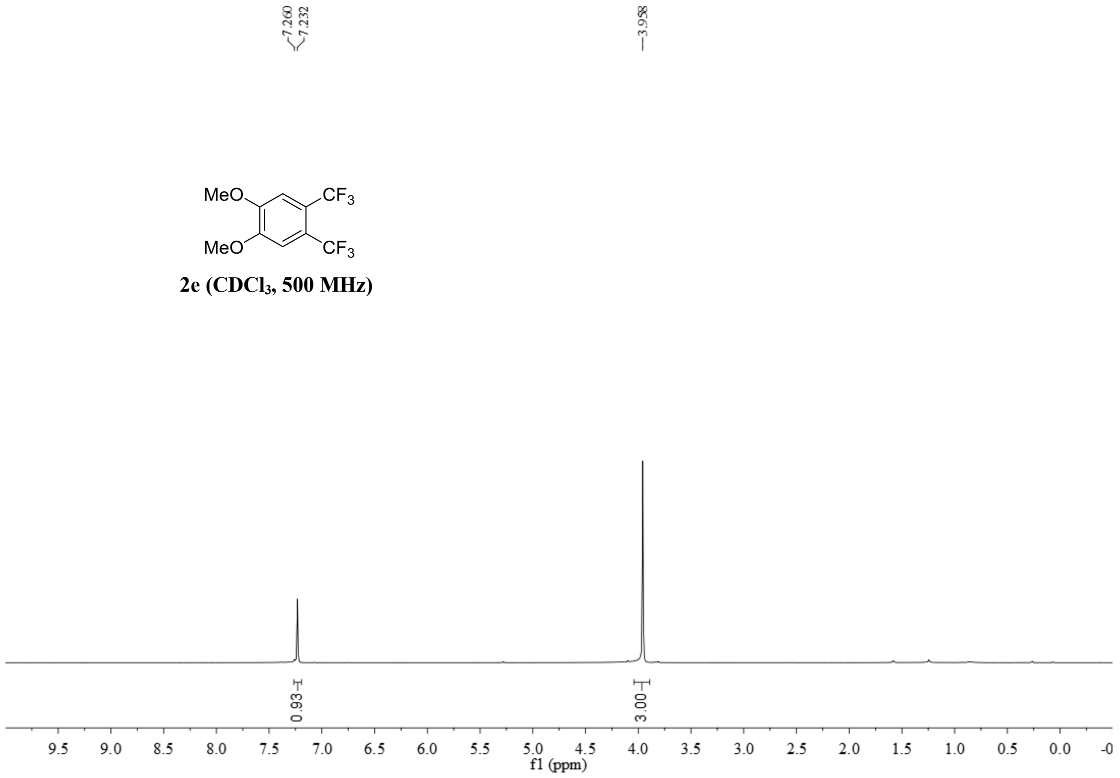

2e ($CDCl_3$, 500 MHz)

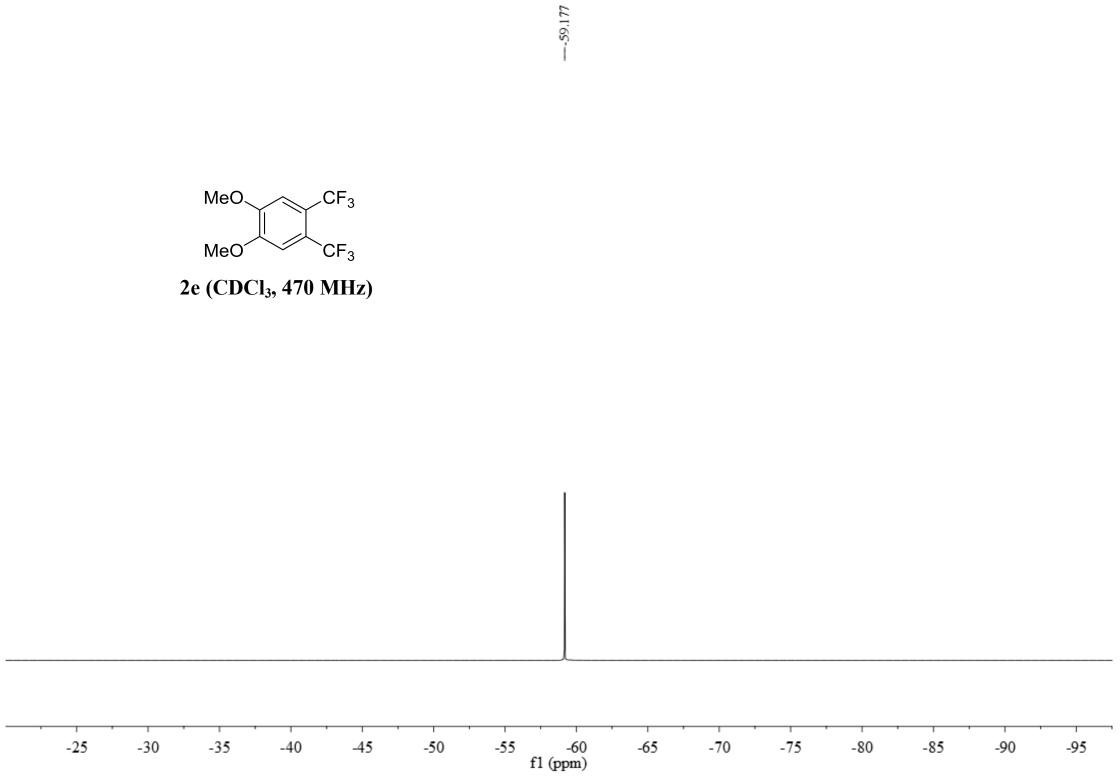

2e ($CDCl_3$, 470 MHz)

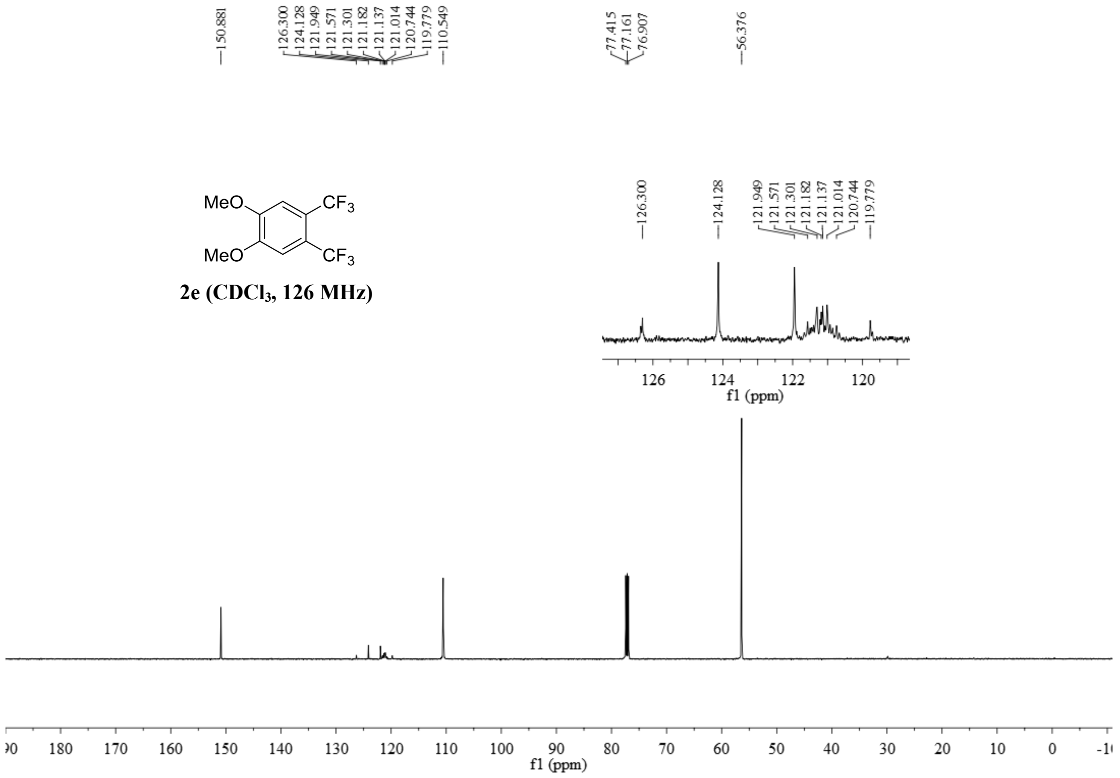

2e (CDCl$_3$, 126 MHz)

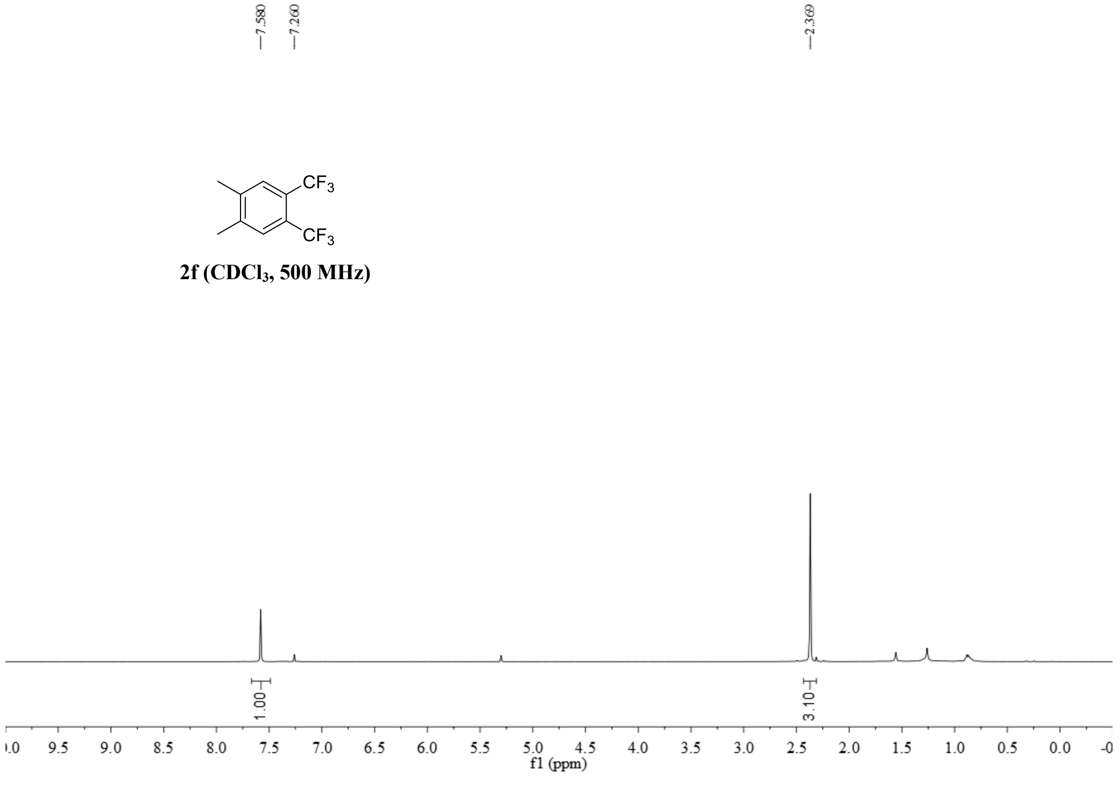

2f (CDCl$_3$, 500 MHz)

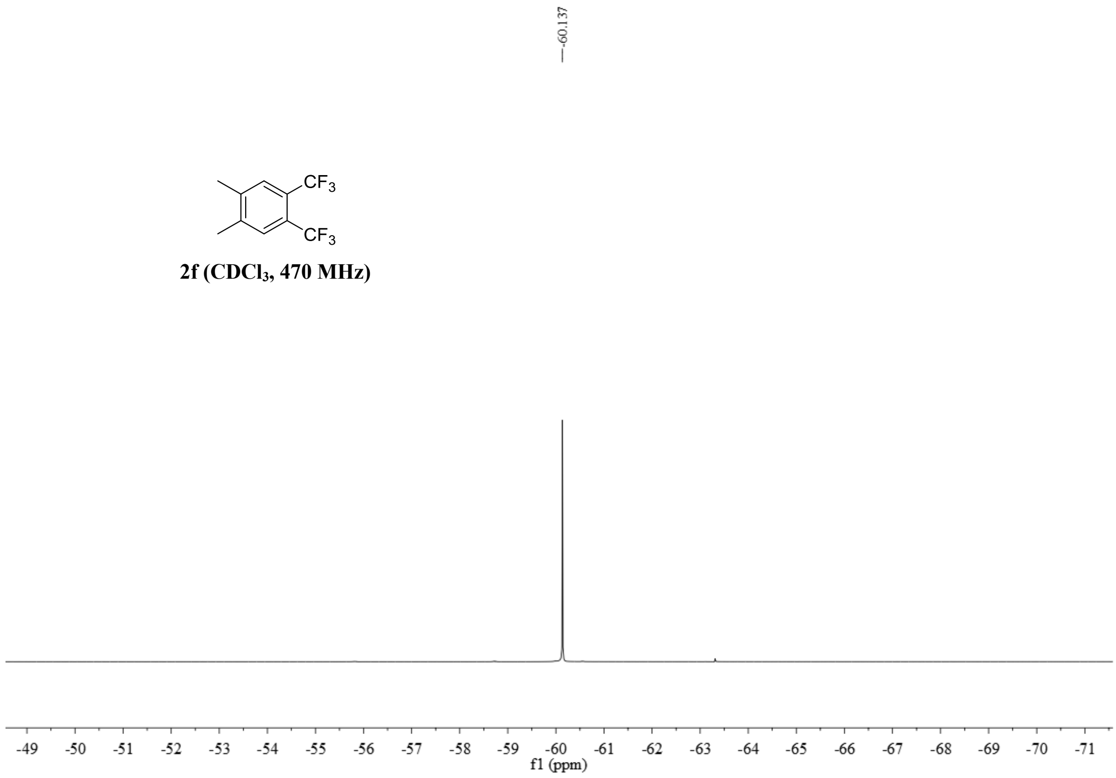
-60.137
CF_3
CF_3
2f (CDCl$_3$, 470 MHz)
-49 -50 -51 -52 -53 -54 -55 -56 -57 -58 -59 -60 -61 -62 -63 -64 -65 -66 -67 -68 -69 -70 -71
f1 (ppm)

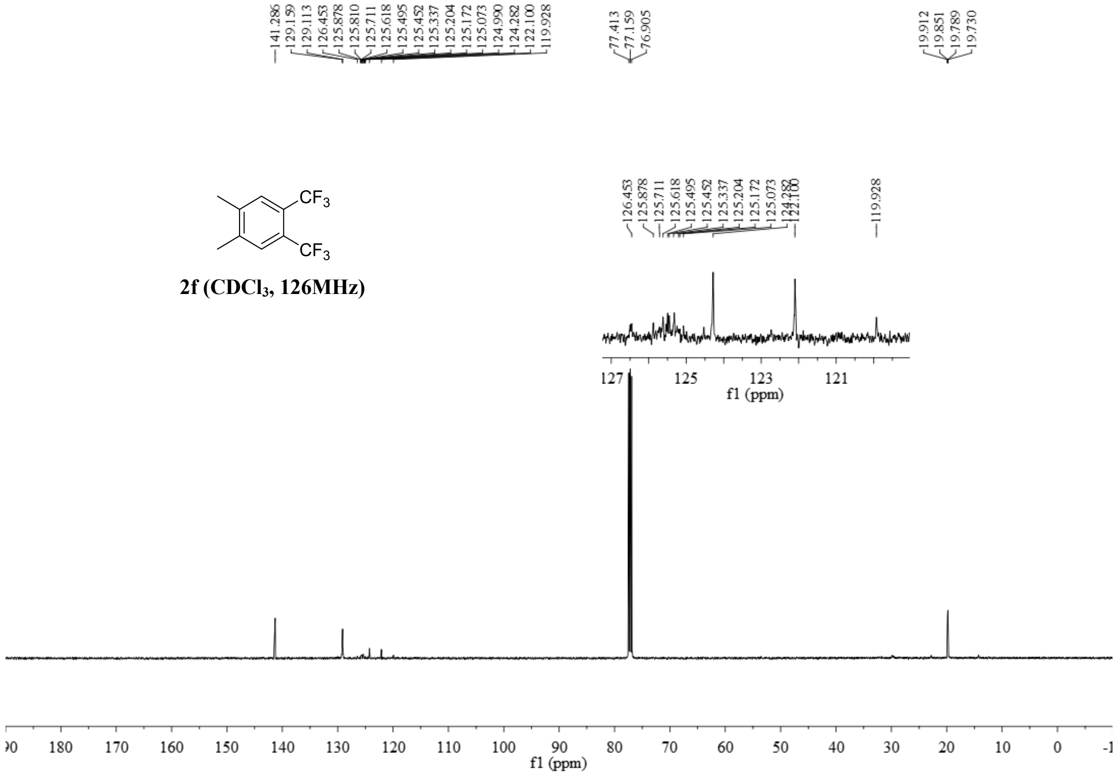
141.286
129.159
129.113
126.453
125.878
125.810
125.711
125.618
125.495
125.452
125.337
125.204
125.172
125.073
124.990
124.282
122.100
119.928
77.413
77.159
76.905
19.912
19.851
19.789
19.730
CF_3
CF_3
2f (CDCl$_3$, 126MHz)
127 125 123 121
f1 (ppm)
180 170 160 150 140 130 120 110 100 90 80 70 60 50 40 30 20 10 0
f1 (ppm)

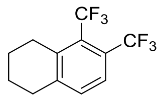

2g (CDCl$_3$, 500 MHz)

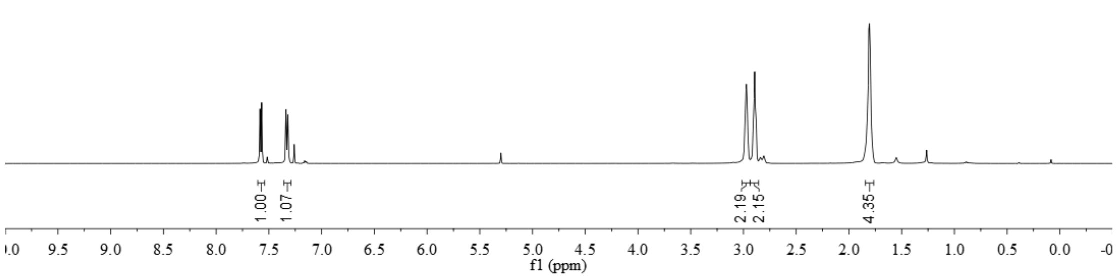

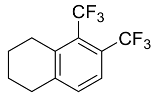

2g (CDCl$_3$, 470 MHz)

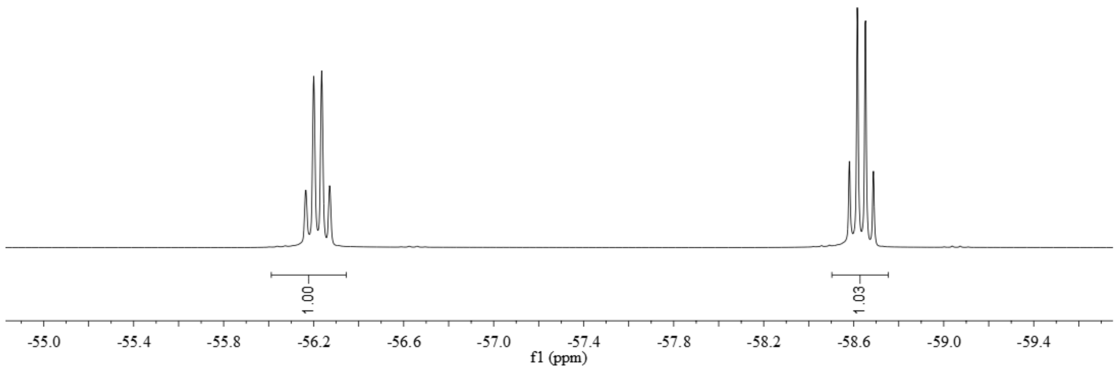

2g (CDCl$_3$, 126 MHz)

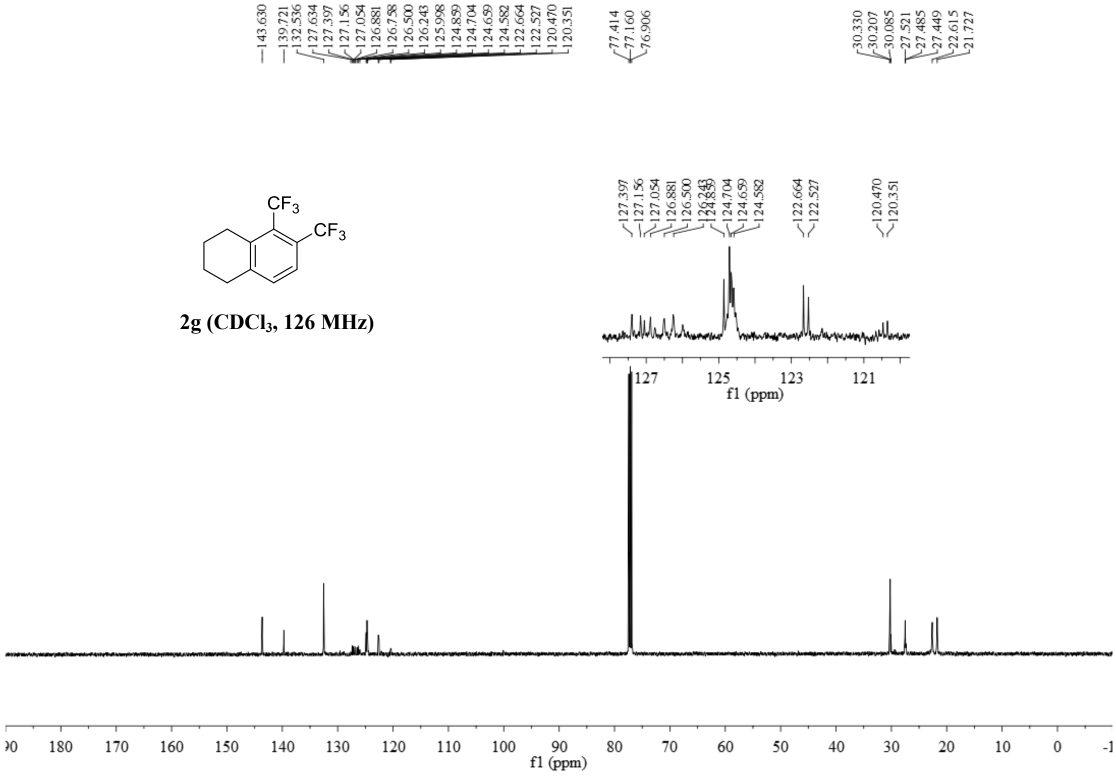

2h (CDCl$_3$, 500 MHz)

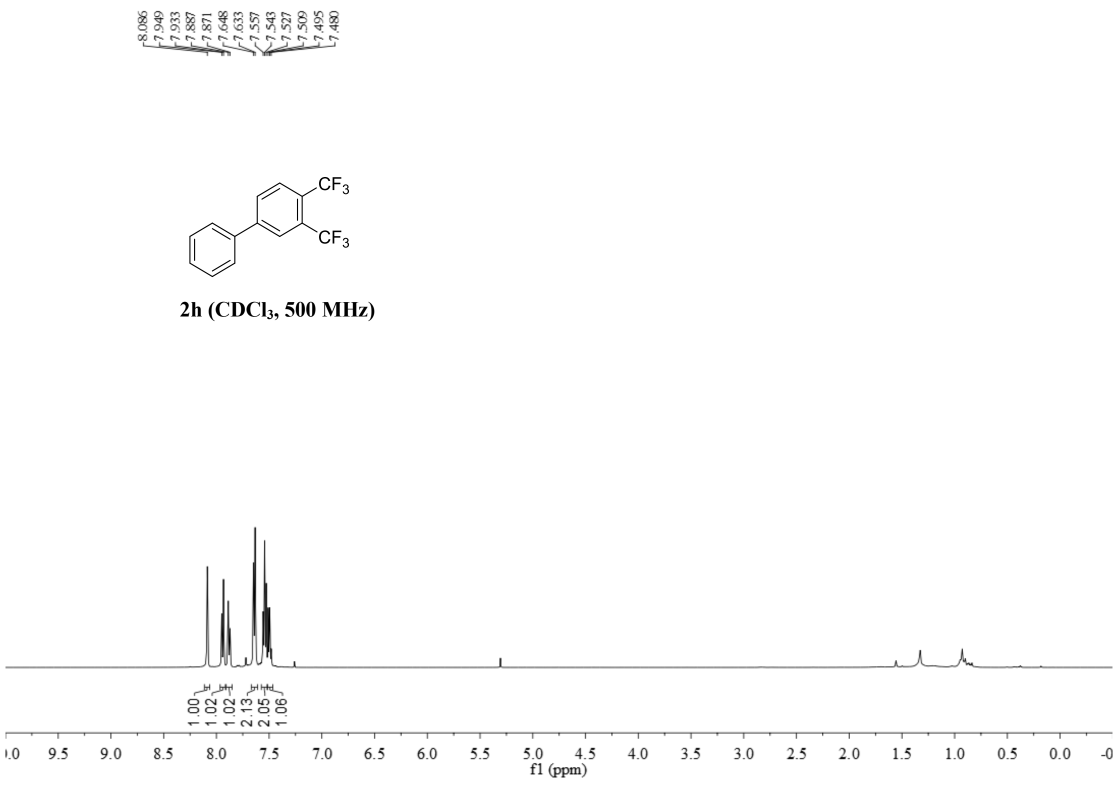

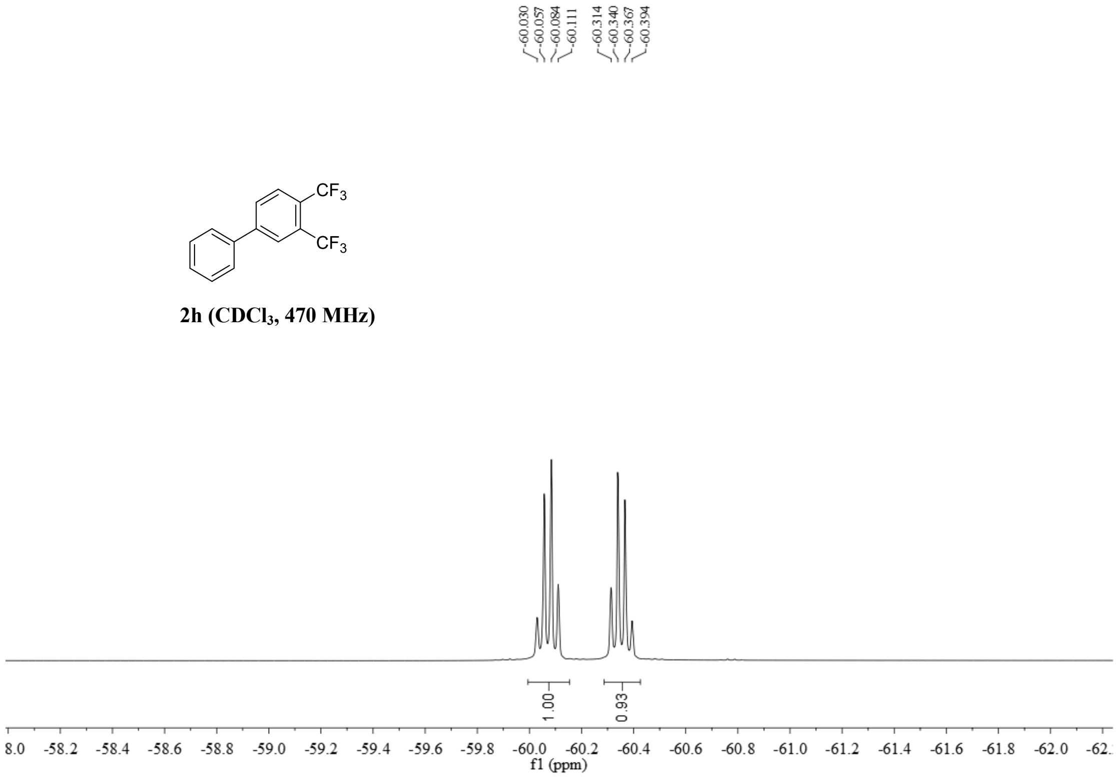

2h ($CDCl_3$, 470 MHz)

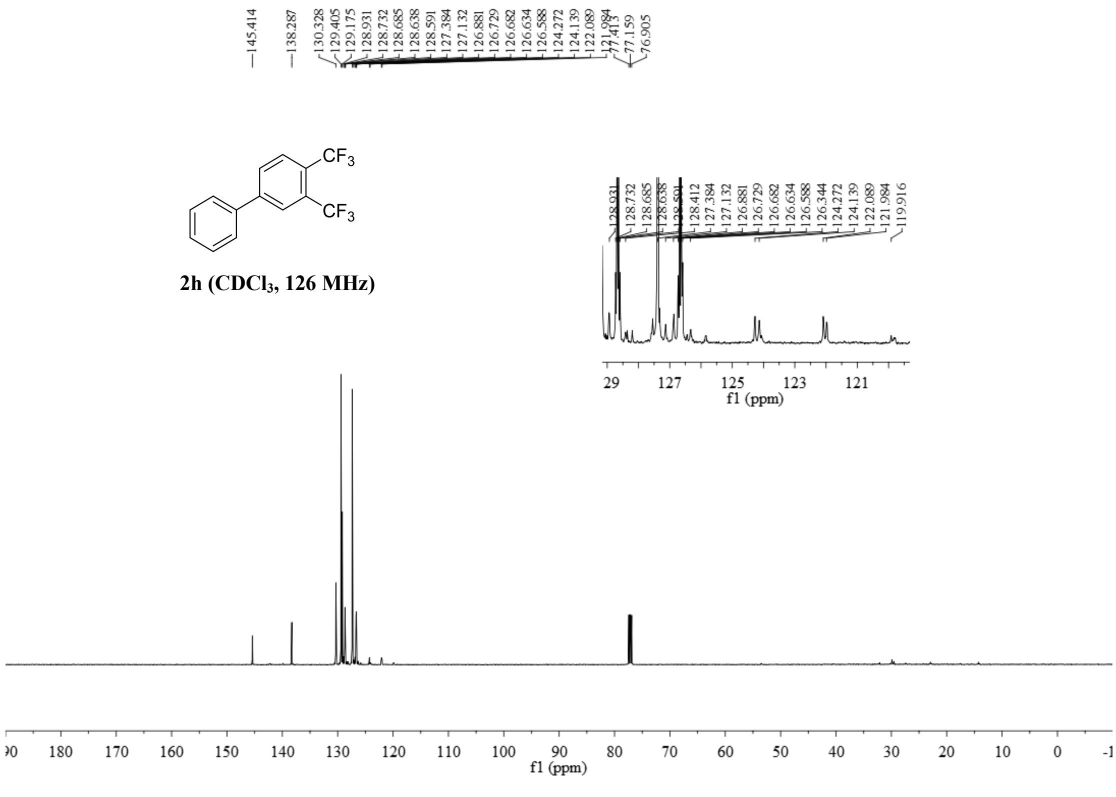

2h ($CDCl_3$, 126 MHz)

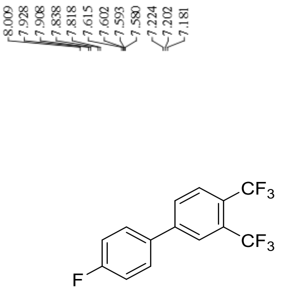

2i ($CDCl_3$, 400 MHz)

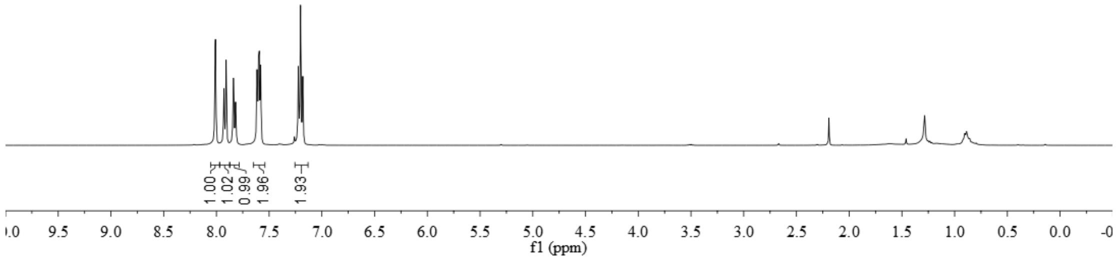

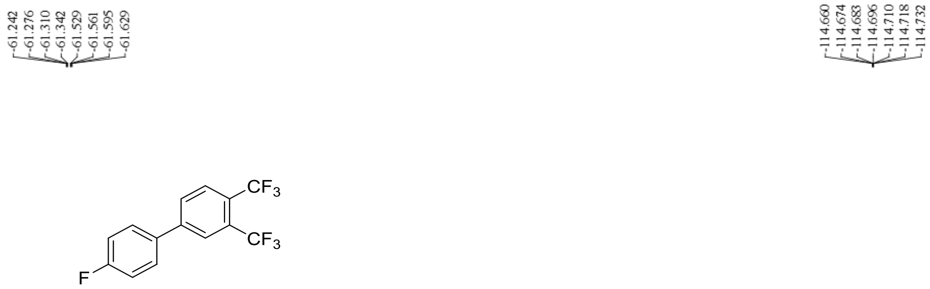

2i ($CDCl_3$, 376 MHz)

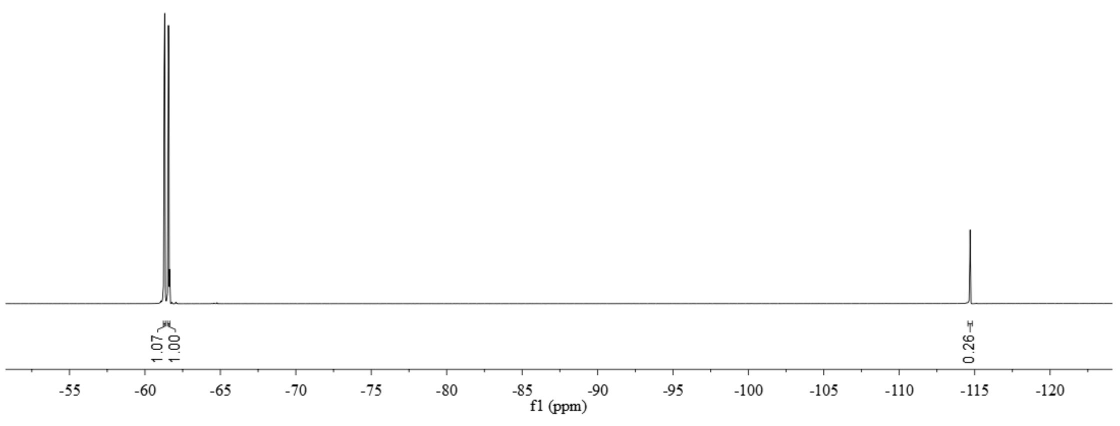

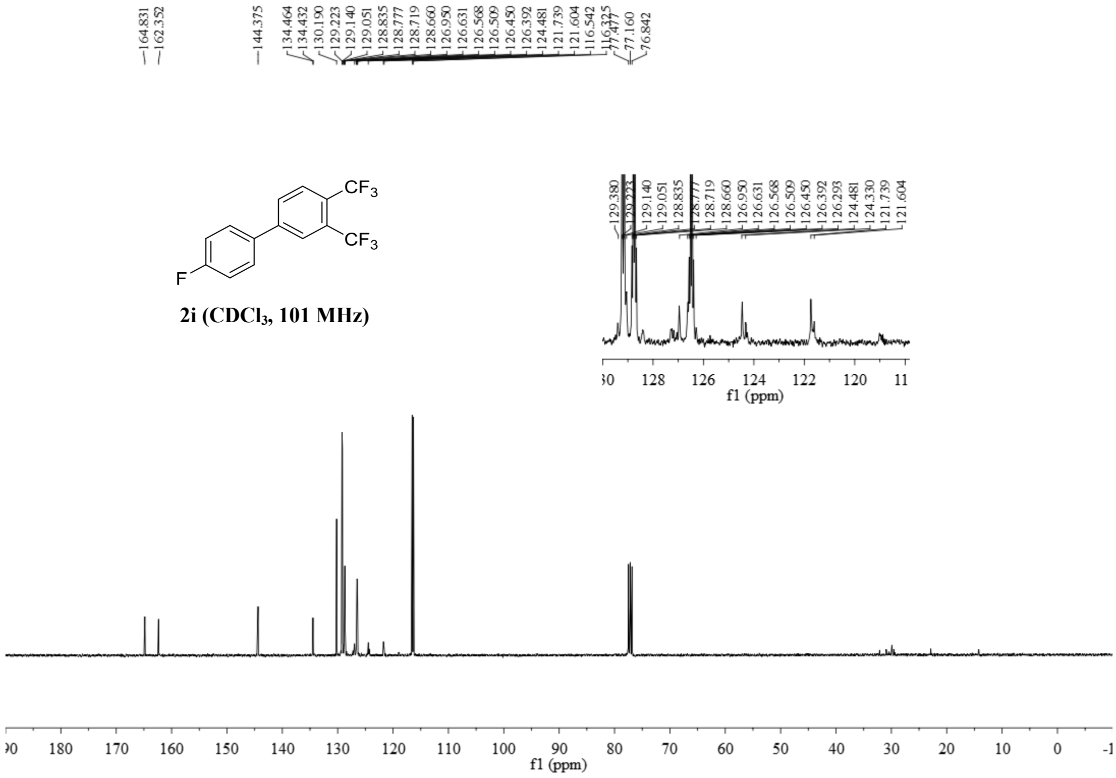

2i (CDCl$_3$, 101 MHz)

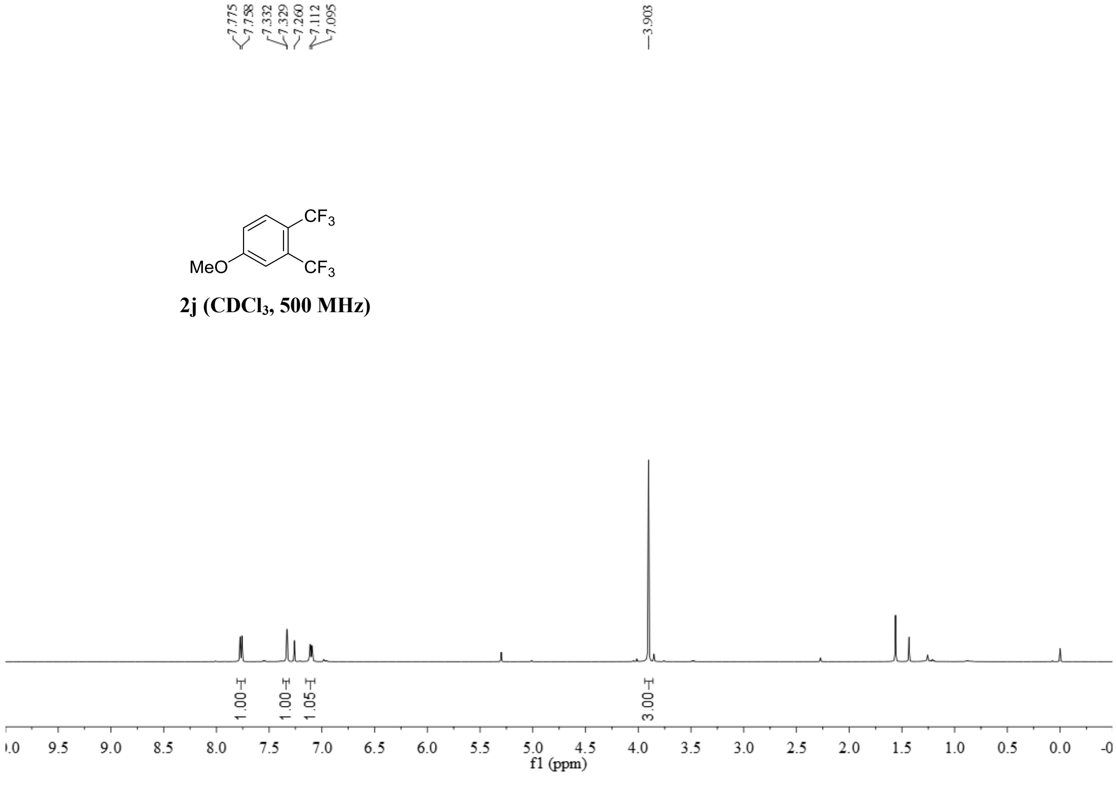

2j (CDCl$_3$, 500 MHz)

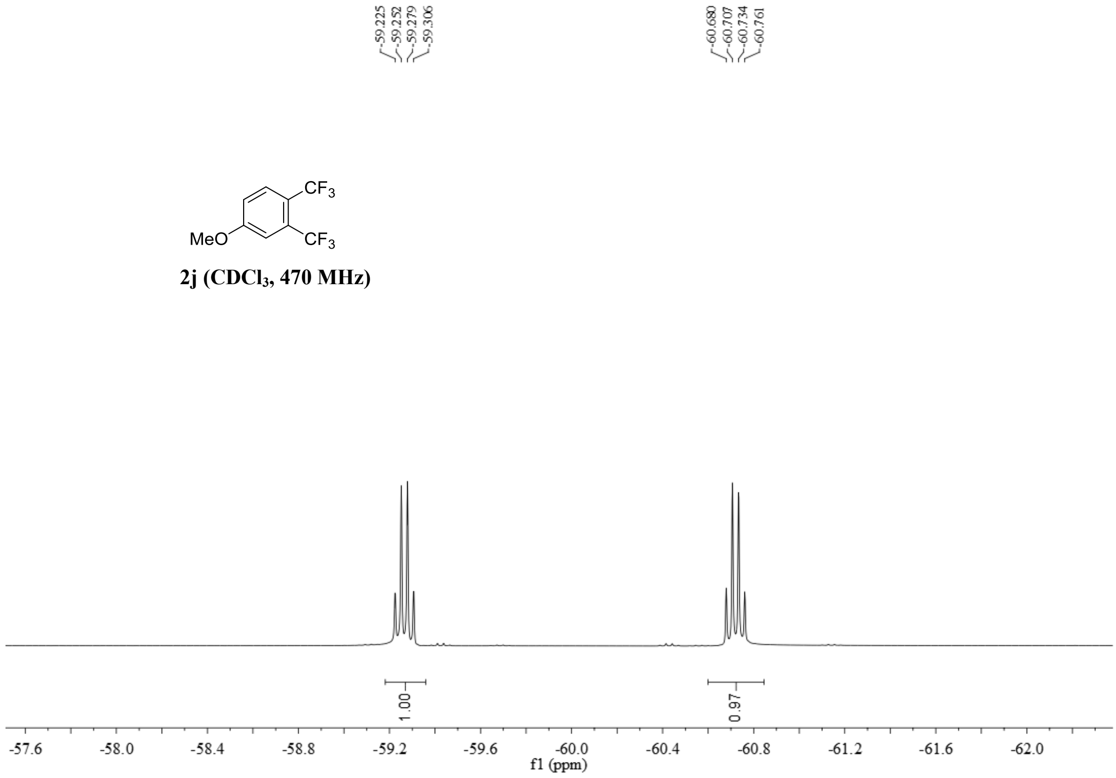

2j (CDCl$_3$, 470 MHz)

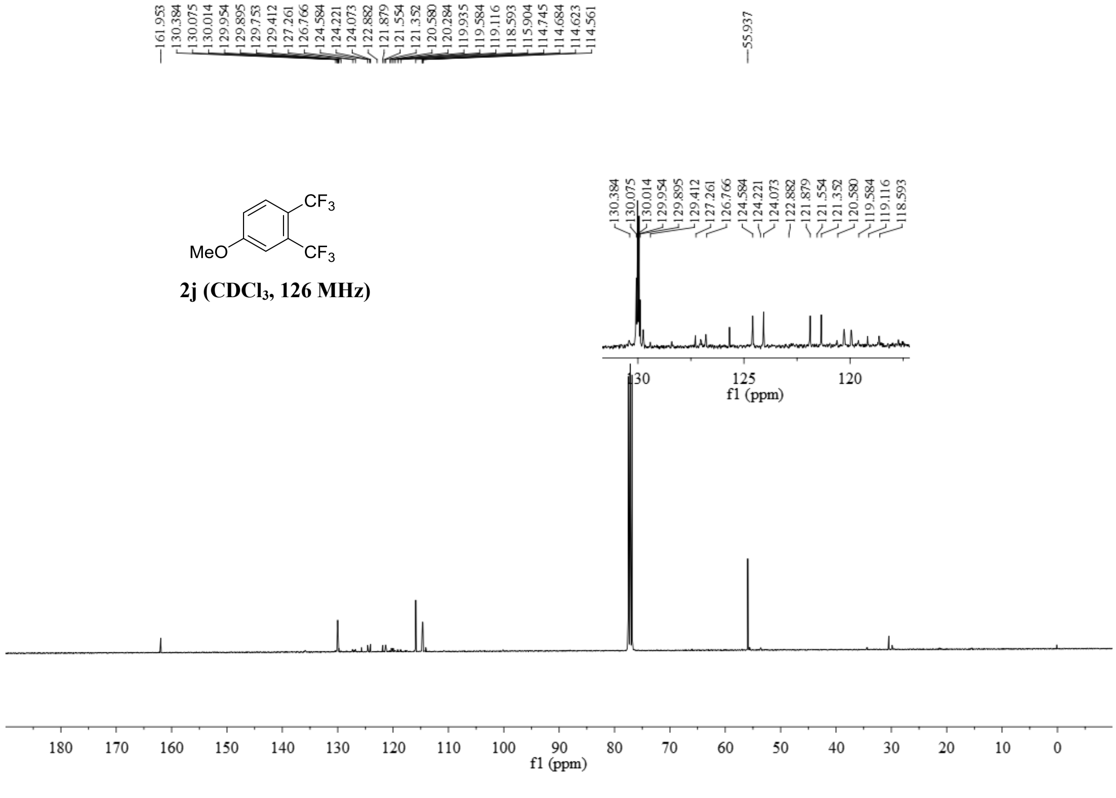

2j (CDCl$_3$, 126 MHz)

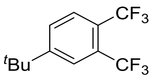

2k ($CDCl_3$, 500 MHz)

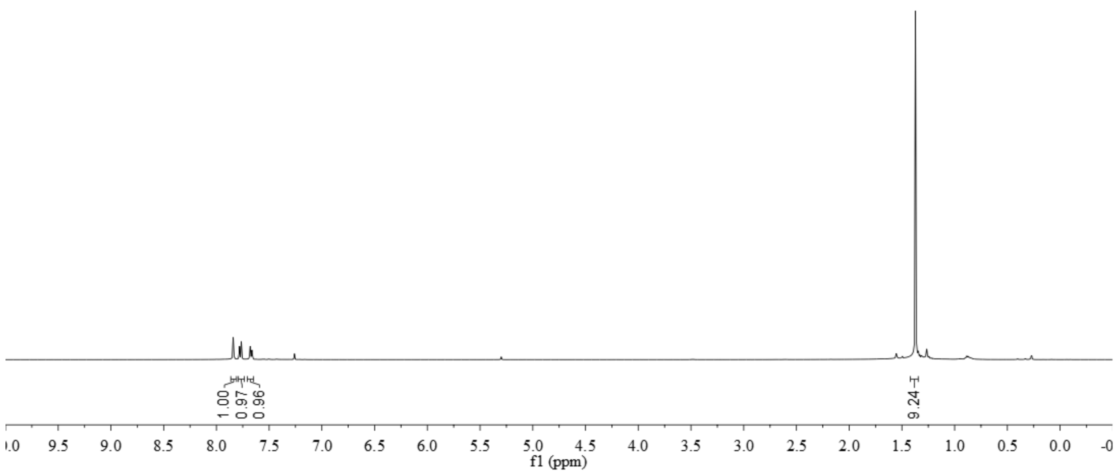

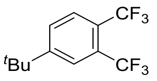

2k ($CDCl_3$, 470 MHz)

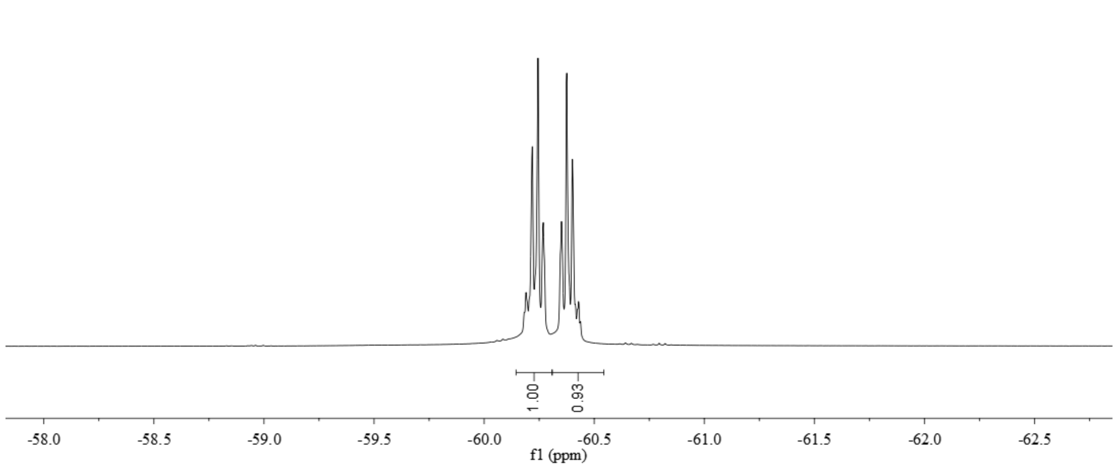

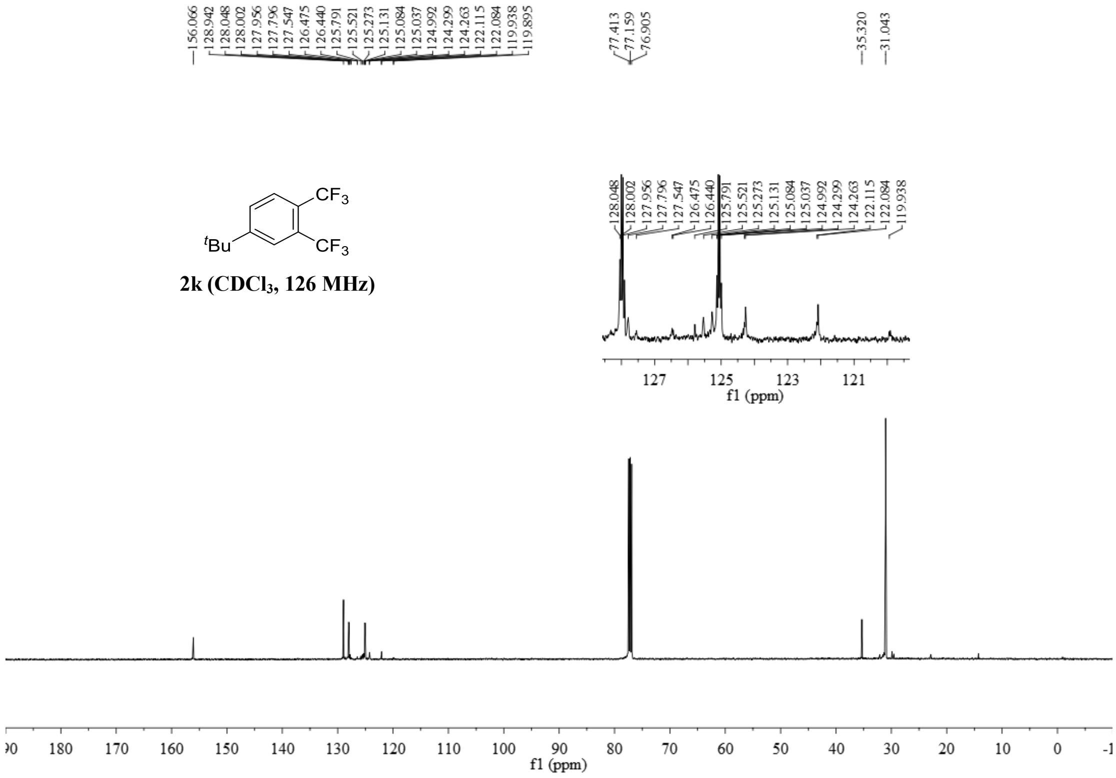

2k (CDCl$_3$, 126 MHz)

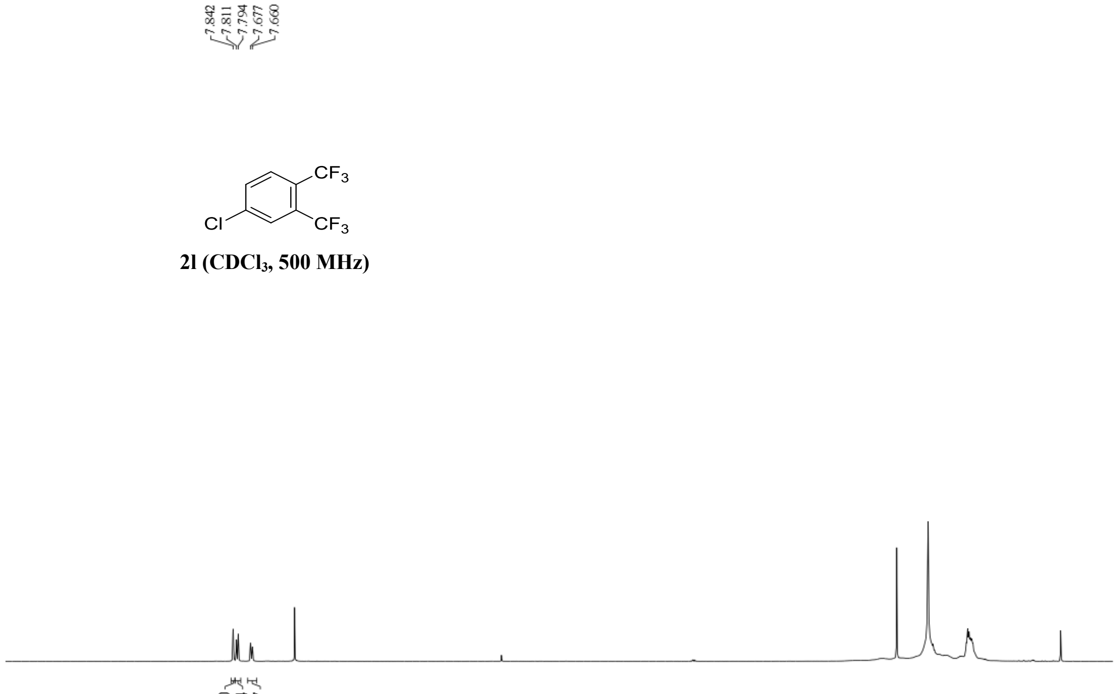

2l (CDCl$_3$, 500 MHz)

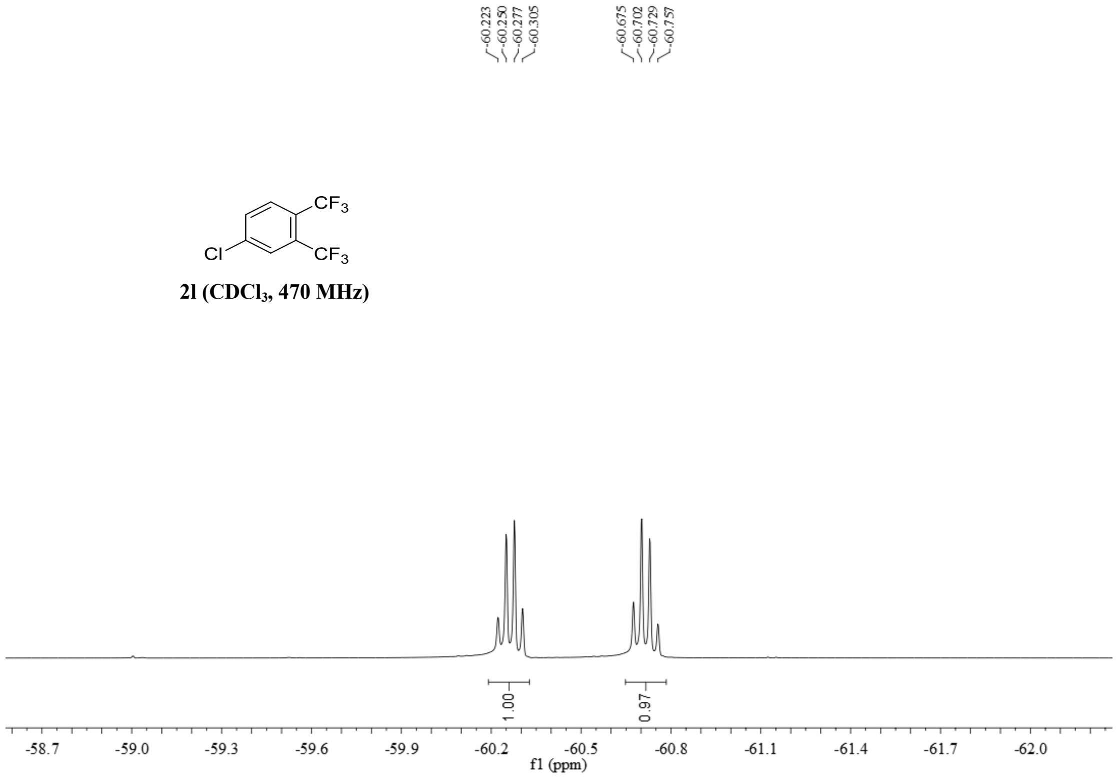

-60.223
-60.250
-60.277
-60.305
-60.675
-60.702
-60.729
-60.757
Cl
CF3
CF3
2l (CDCl3, 470 MHz)
1.00
0.97
-58.7 -59.0 -59.3 -59.6 -59.9 -60.2 -60.5 -60.8 -61.1 -61.4 -61.7 -62.0
f1 (ppm)

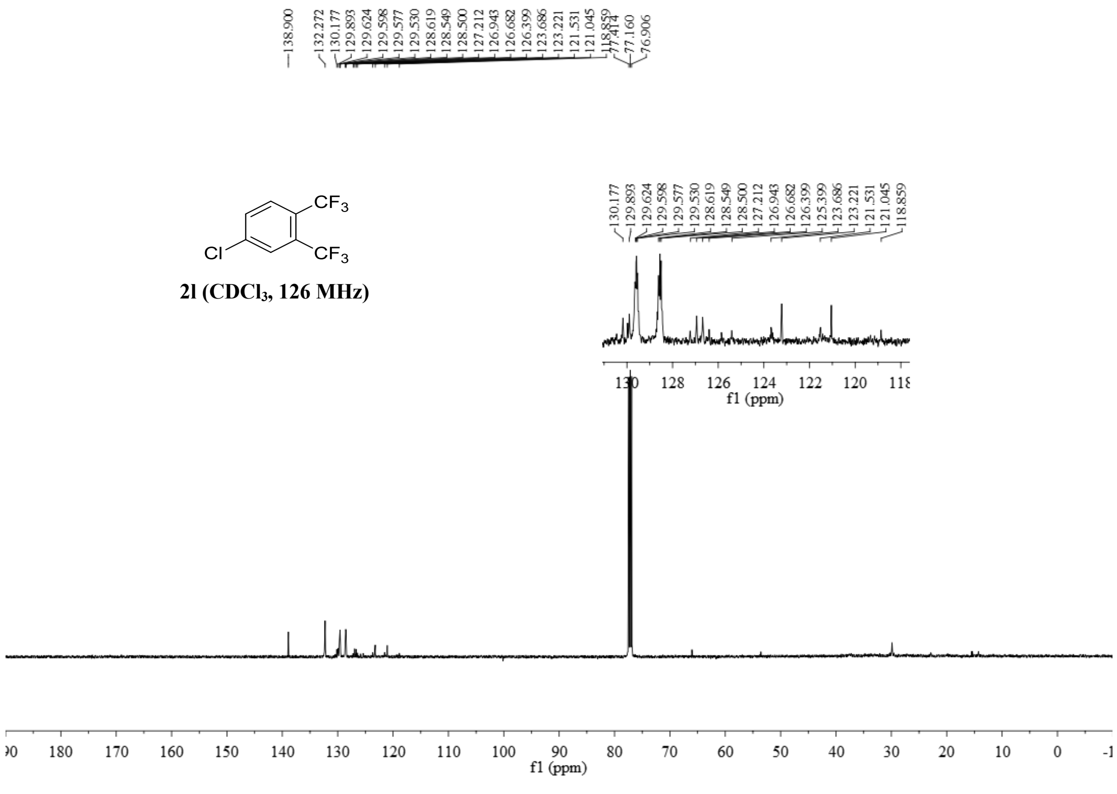

138.900
132.272
130.177
129.893
129.624
129.598
129.577
129.530
128.619
128.549
128.500
127.212
126.943
126.682
126.399
123.686
123.221
121.531
121.045
118.859
77.414
77.160
76.906
Cl
CF3
CF3
2l (CDCl3, 126 MHz)
130 128 126 124 122 120
f1 (ppm)
180 170 160 150 140 130 120 110 100 90 80 70 60 50 40 30 20 10 0
f1 (ppm)

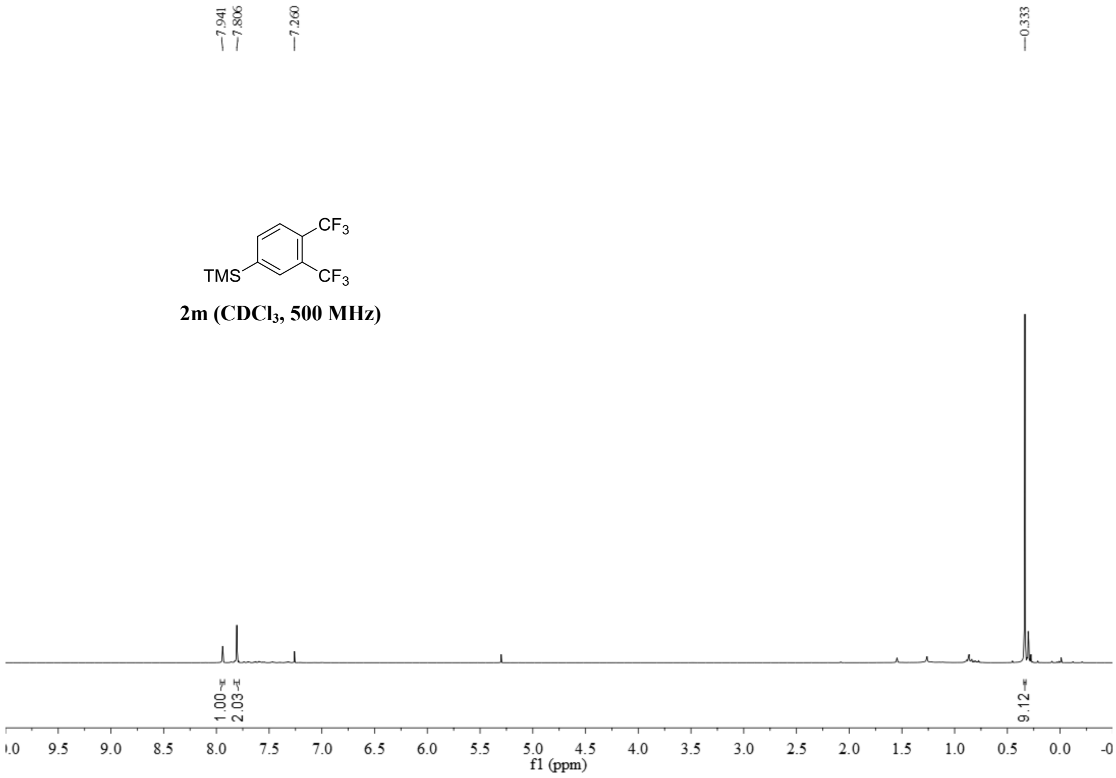

2m ($CDCl_3$, 500 MHz)

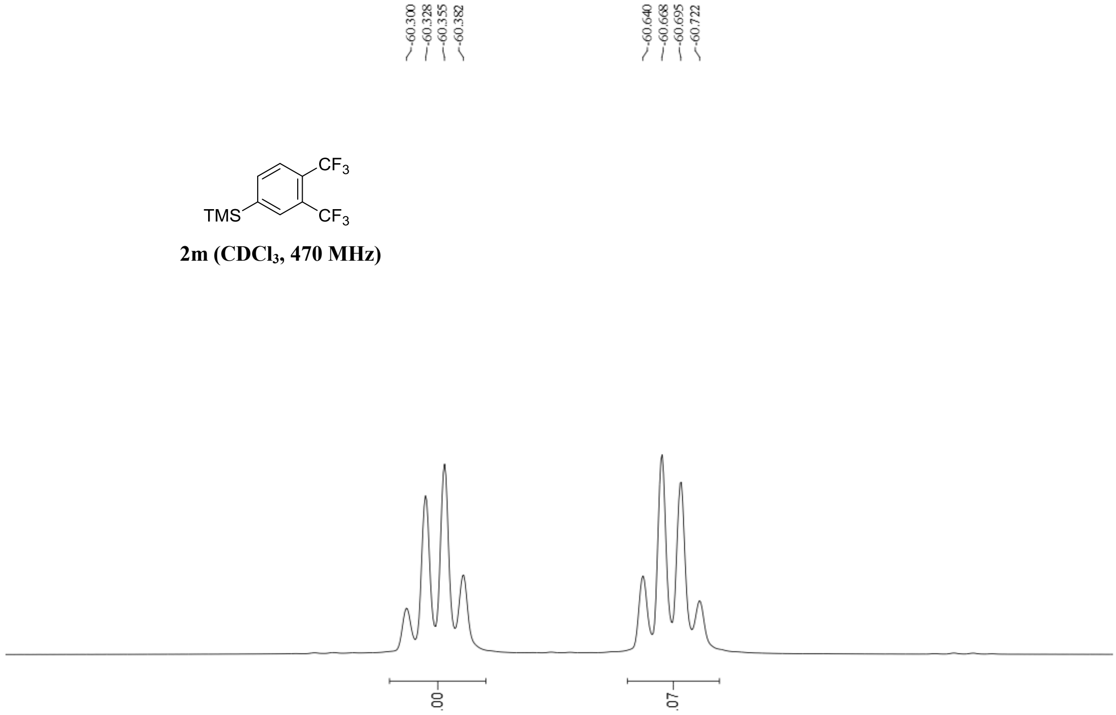

2m ($CDCl_3$, 470 MHz)

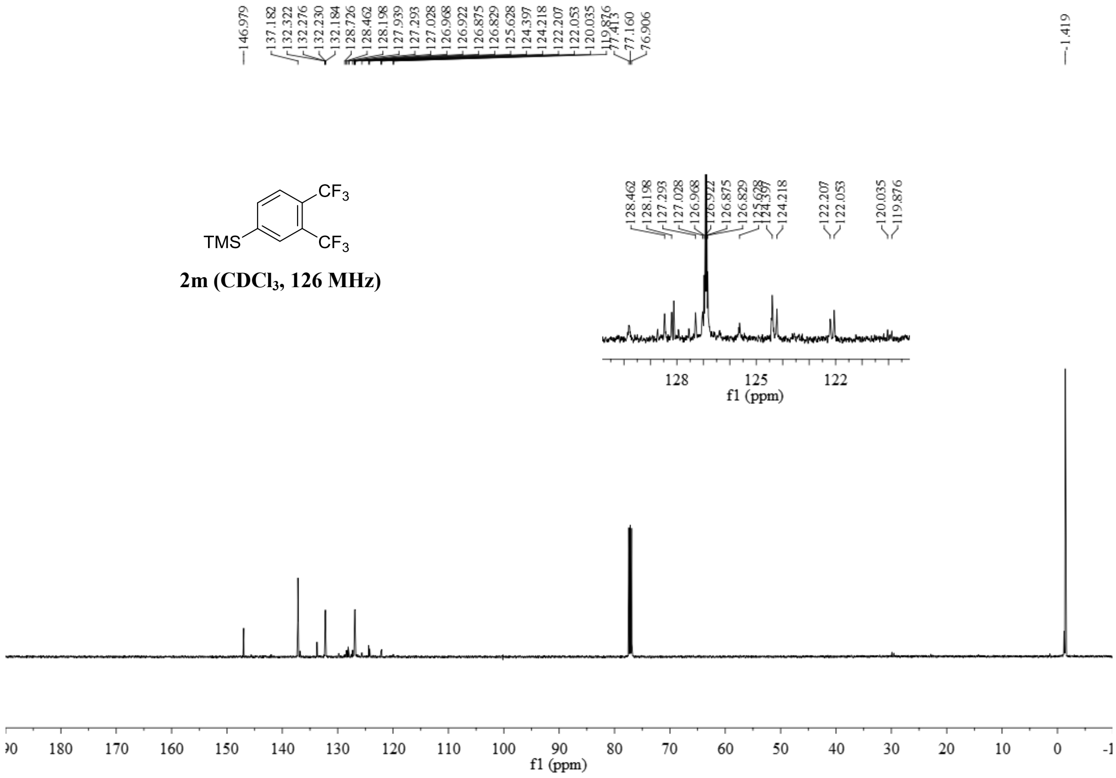

2m (CDCl$_3$, 126 MHz)

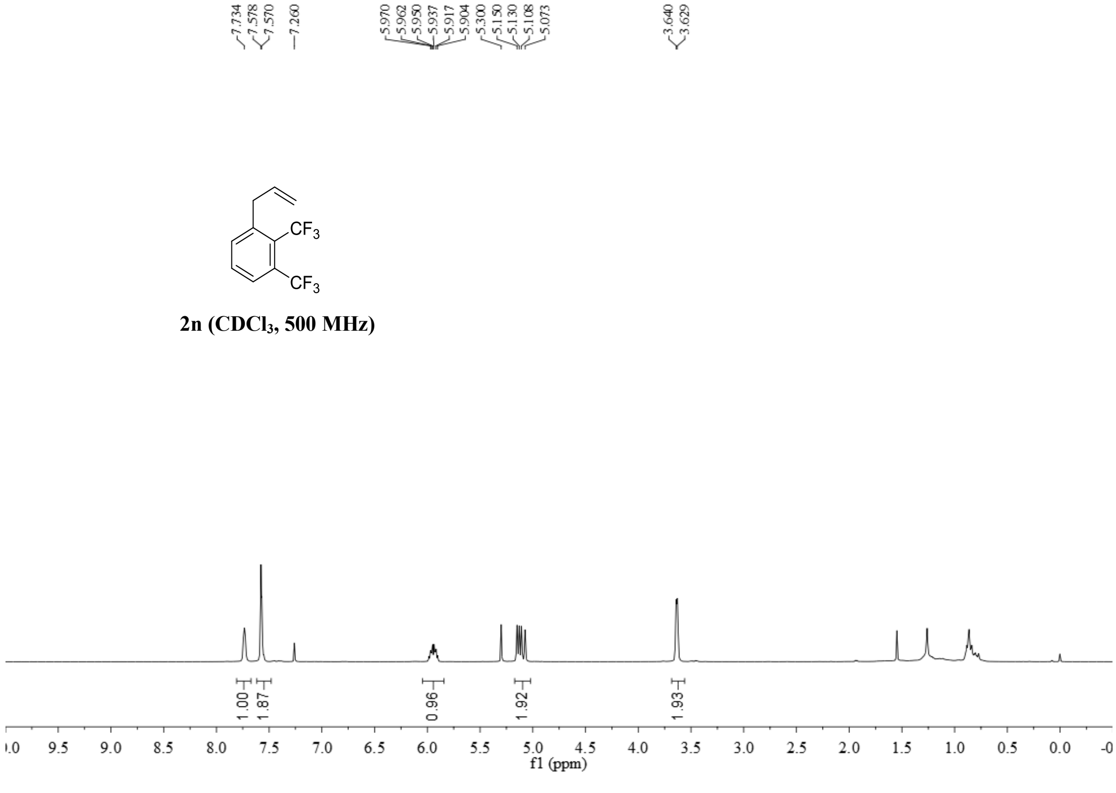

2n (CDCl$_3$, 500 MHz)

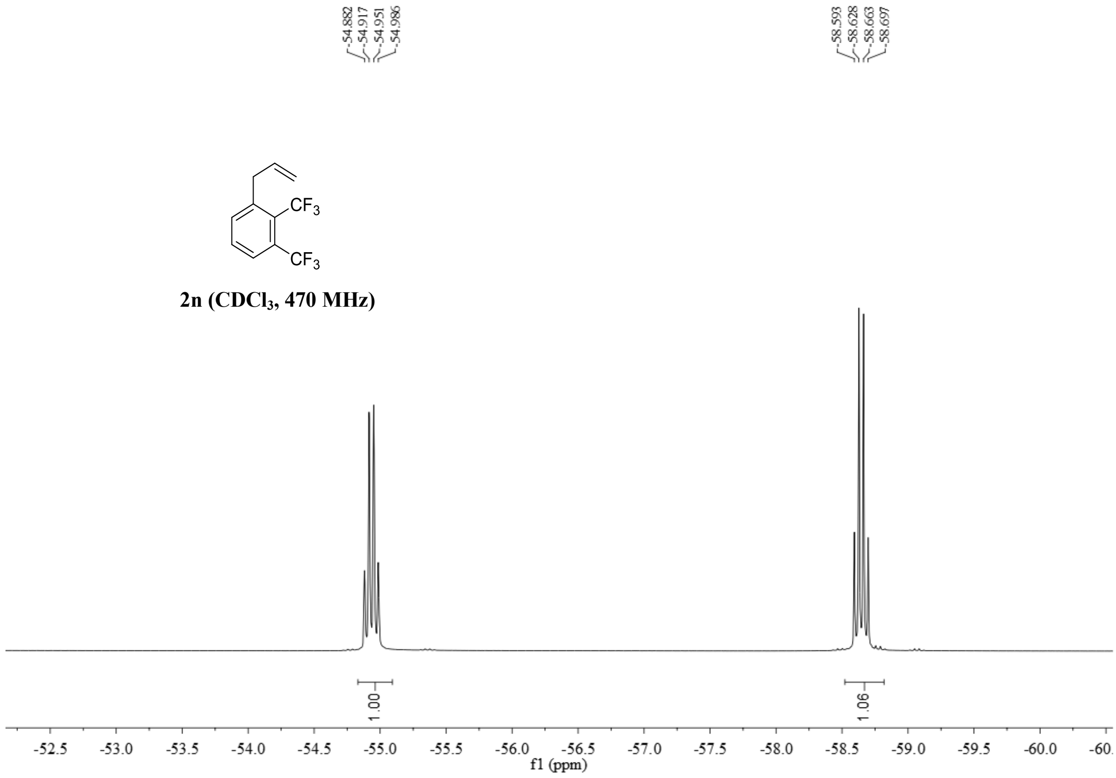

2n ($CDCl_3$, 470 MHz)

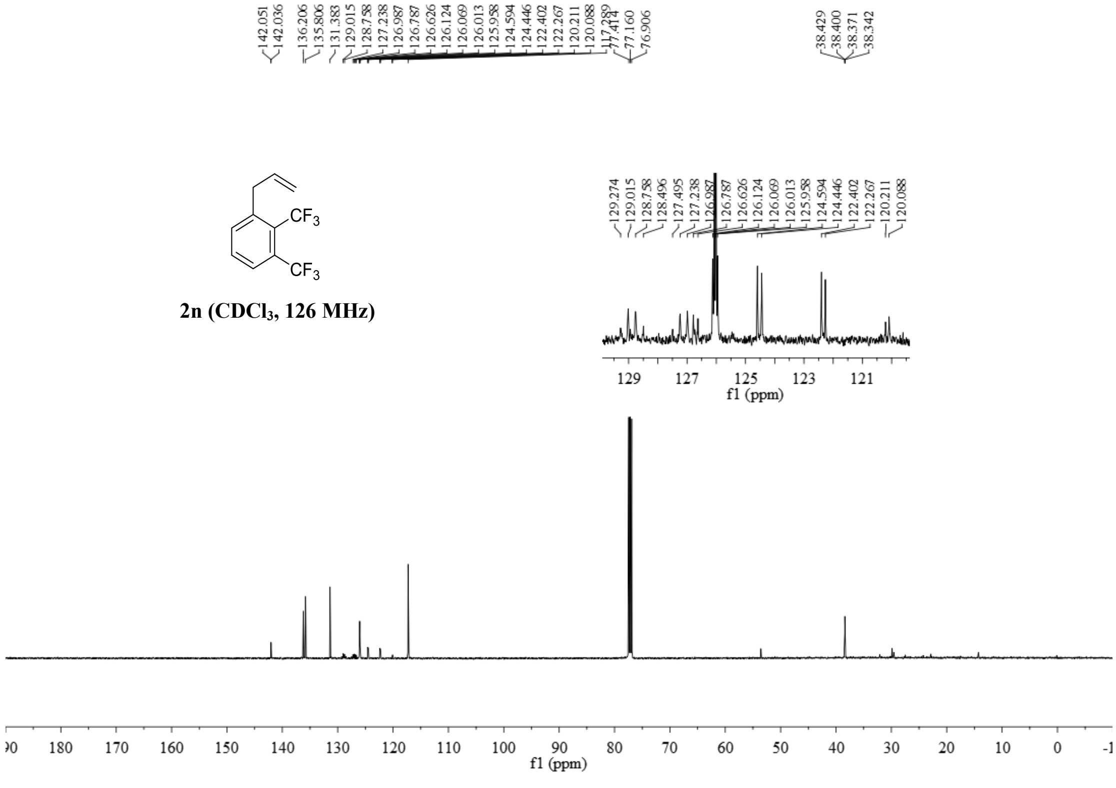

2n ($CDCl_3$, 126 MHz)

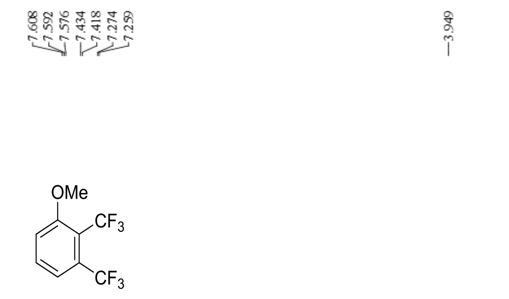

2o (CDCl$_3$, 500 MHz)

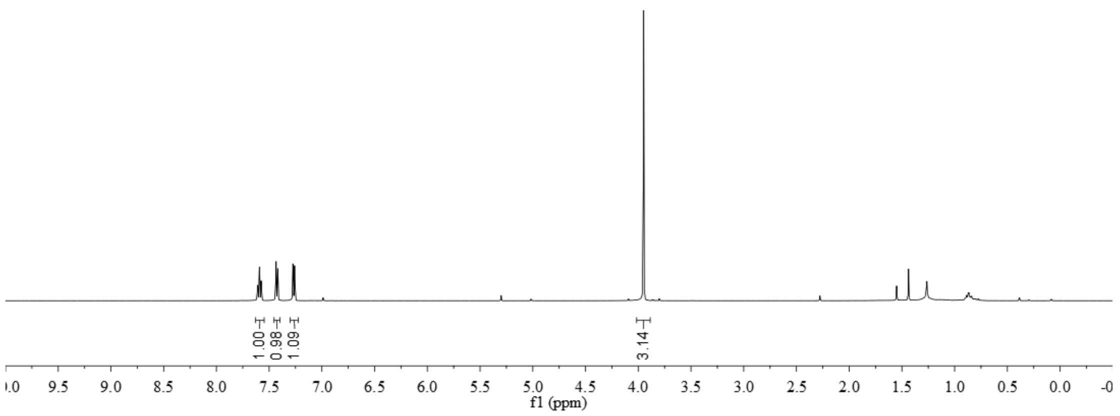

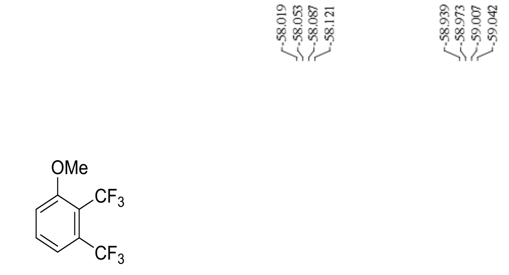

2o (CDCl$_3$, 470 MHz)

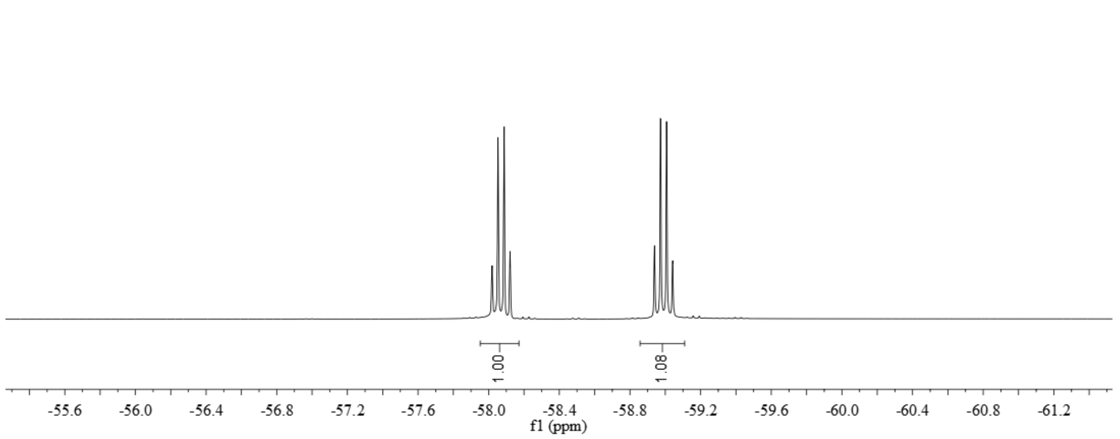

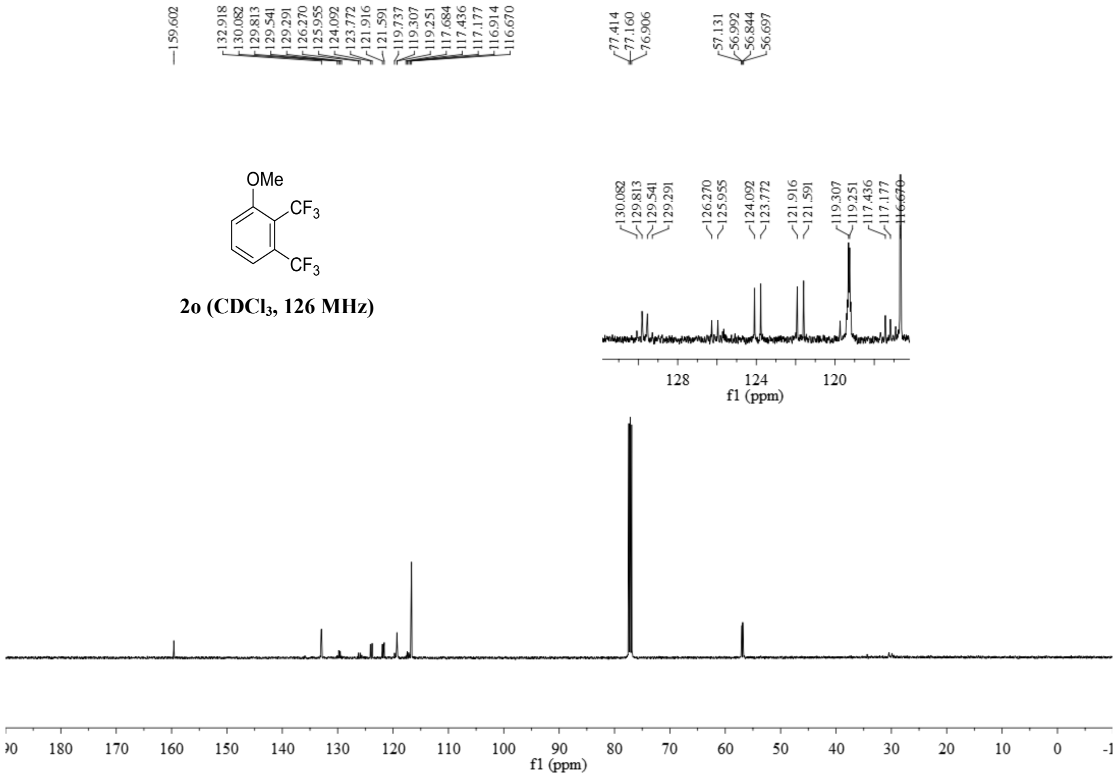

2o ($CDCl_3$, 126 MHz)

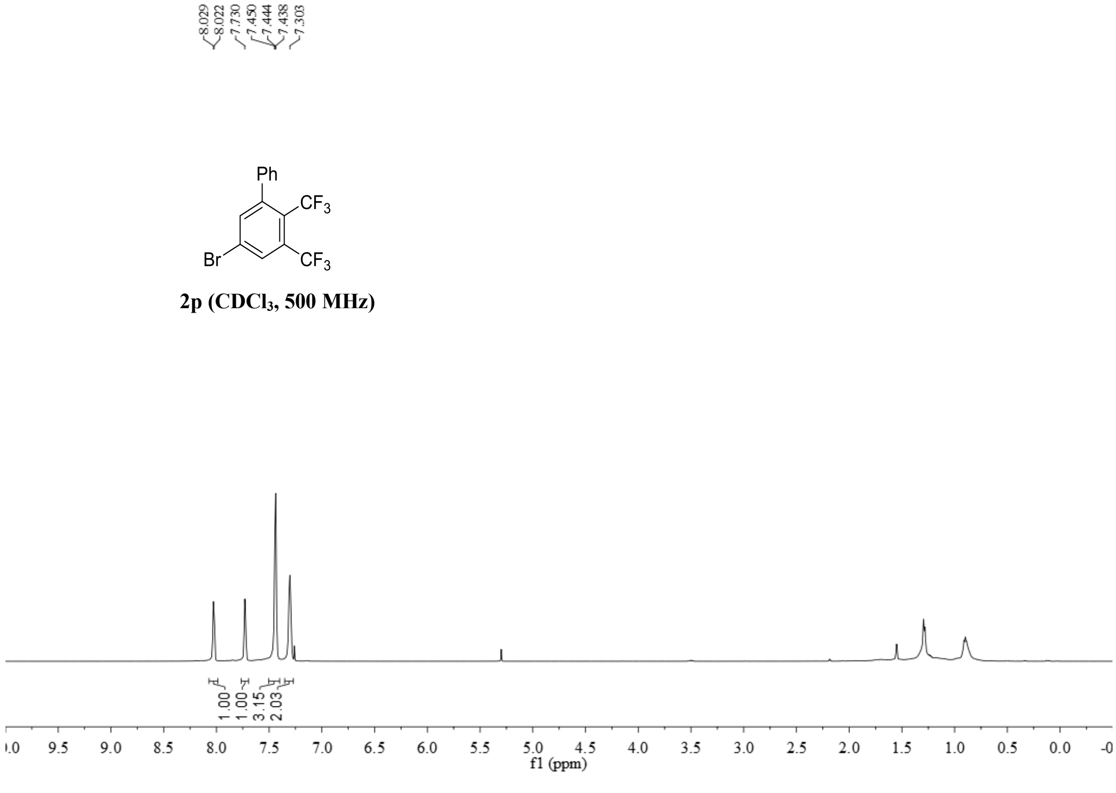

2p ($CDCl_3$, 500 MHz)

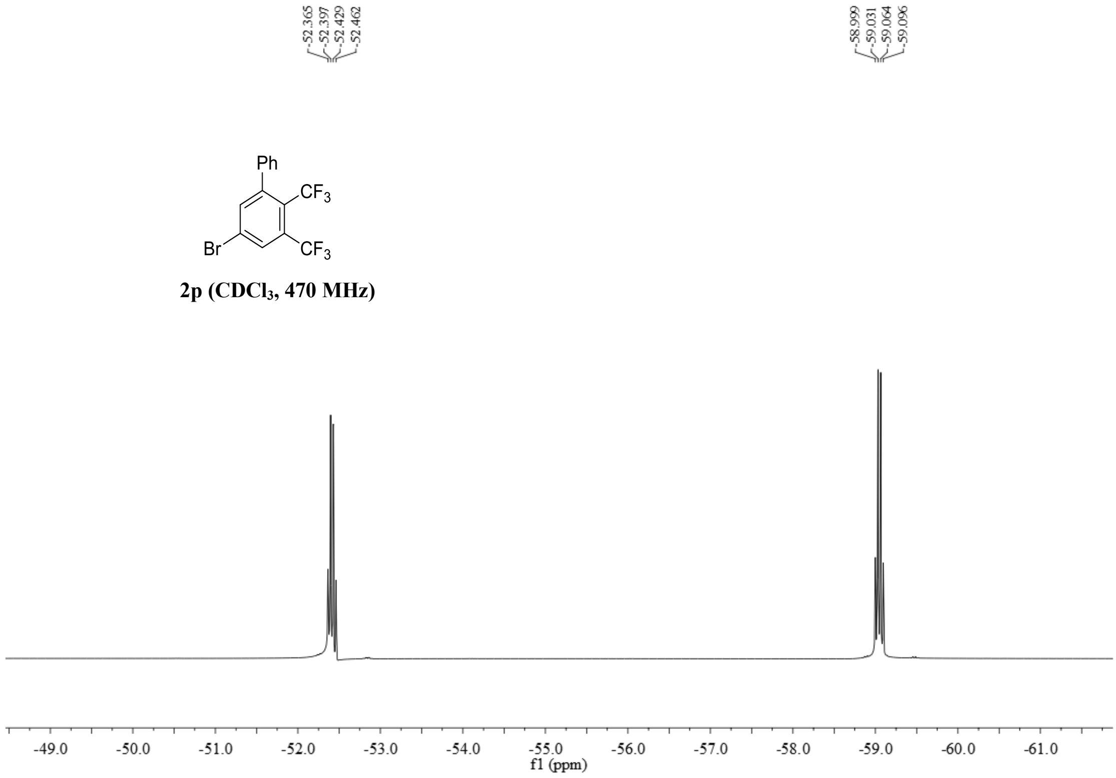

2p ($CDCl_3$, 470 MHz)

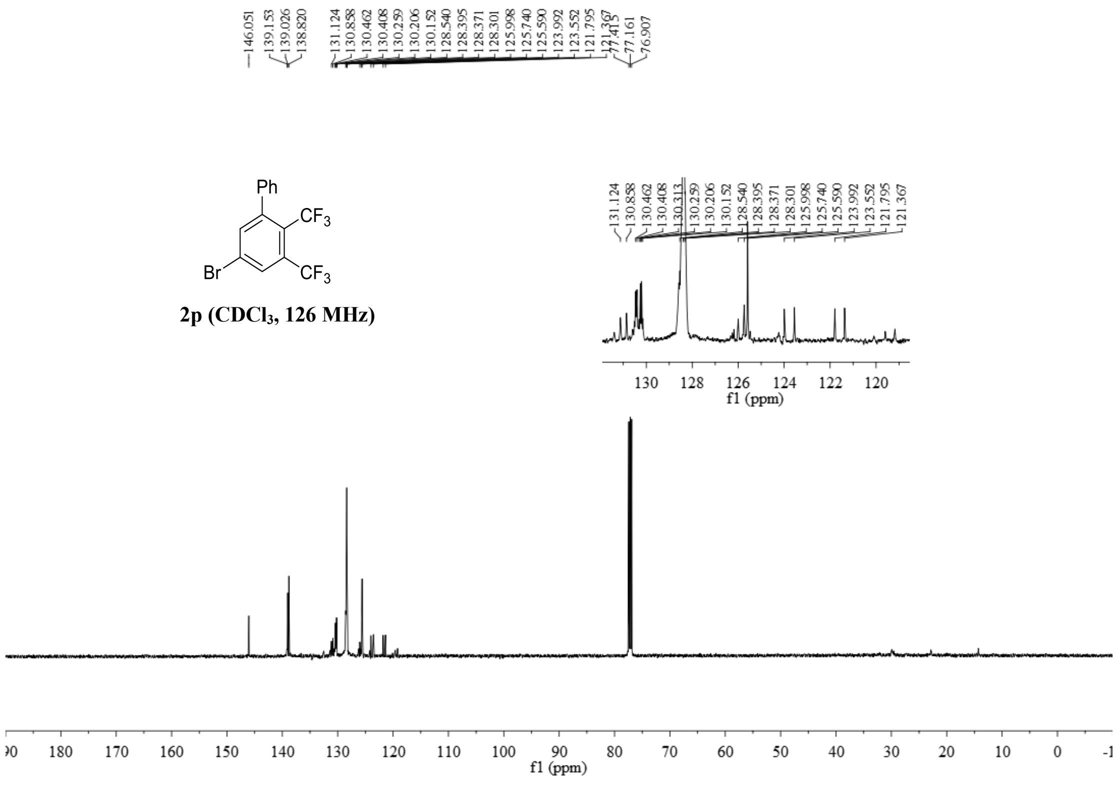

2p ($CDCl_3$, 126 MHz)

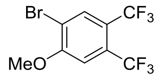

2q ($CDCl_3$, 500 MHz)

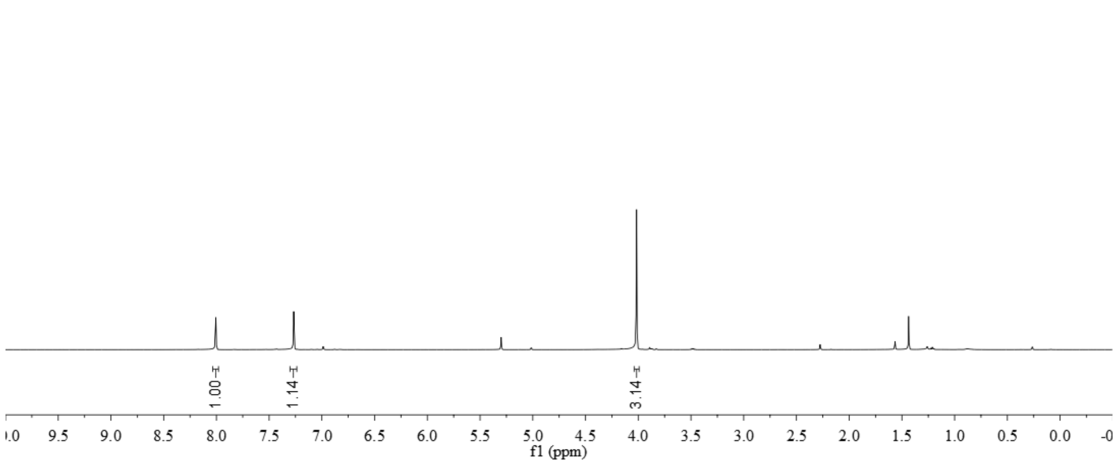

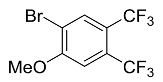

2q ($CDCl_3$, 470 MHz)

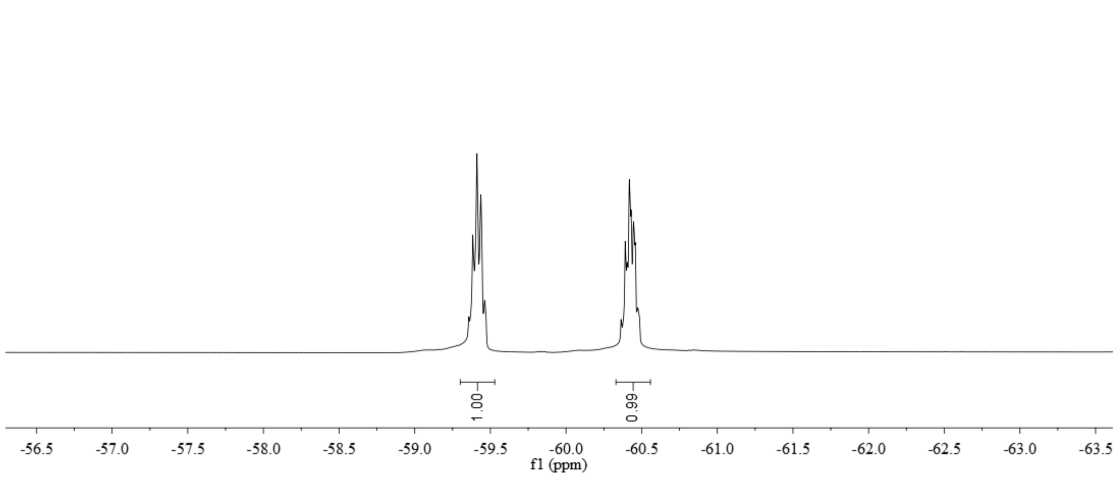

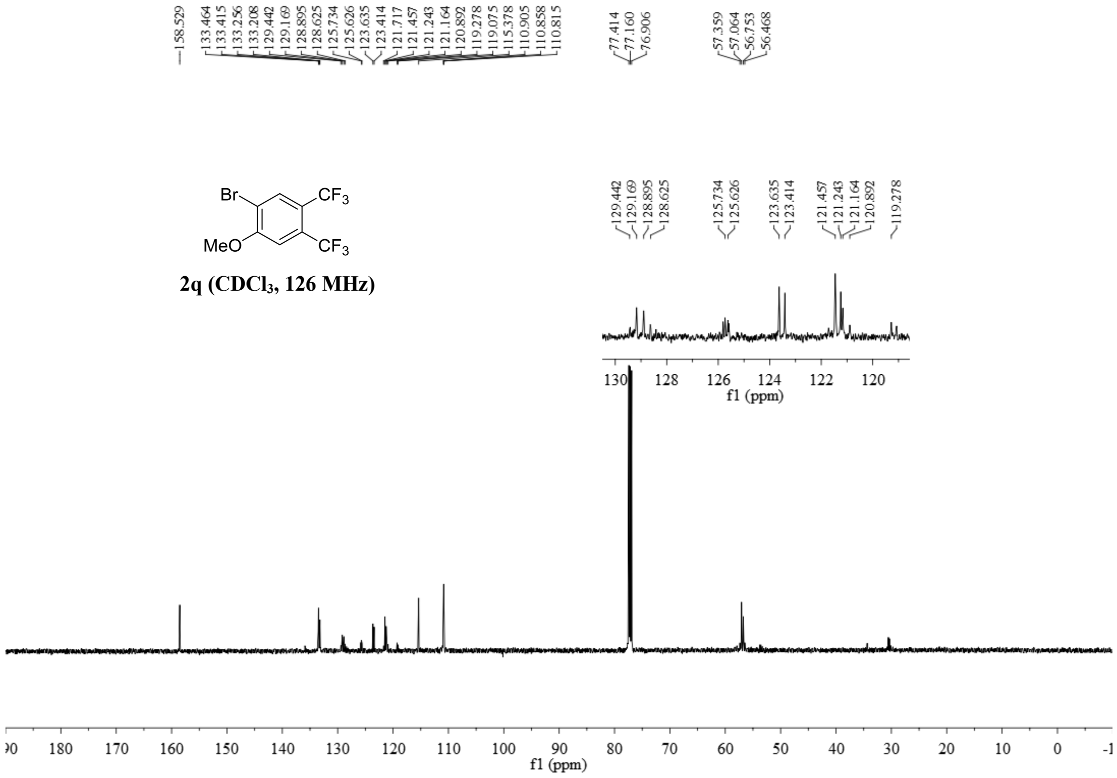

2q ($CDCl_3$, 126 MHz)

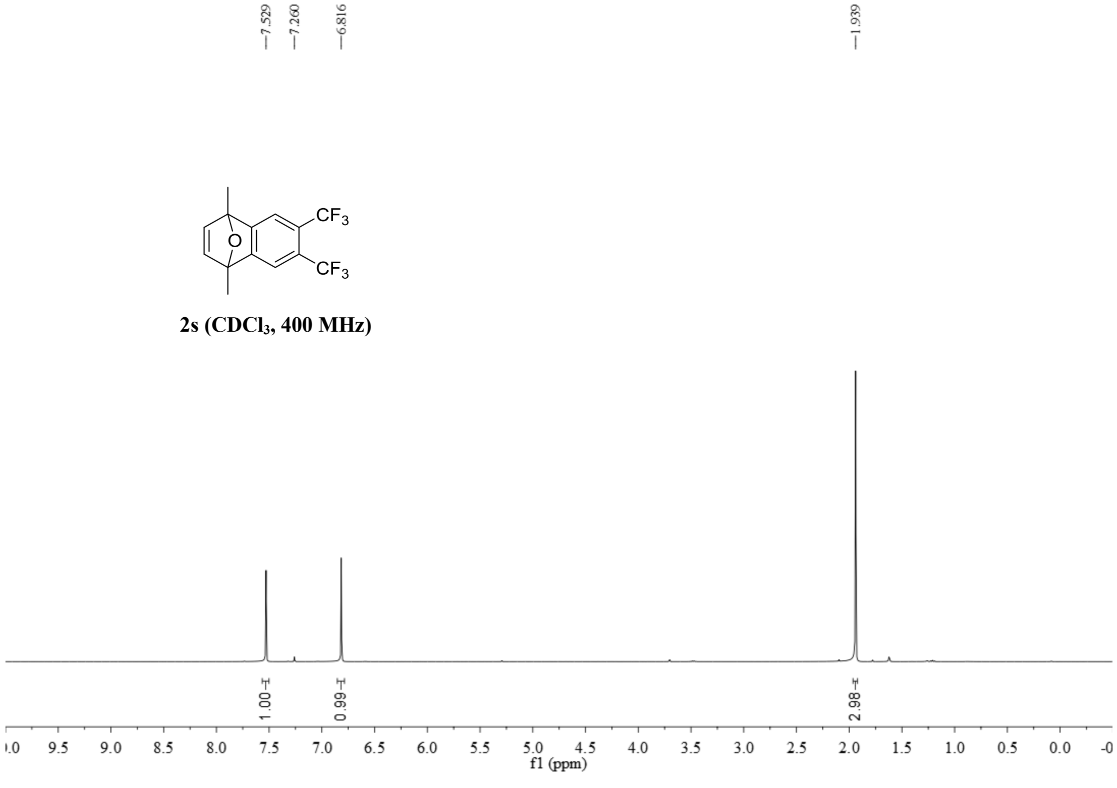

2s ($CDCl_3$, 400 MHz)

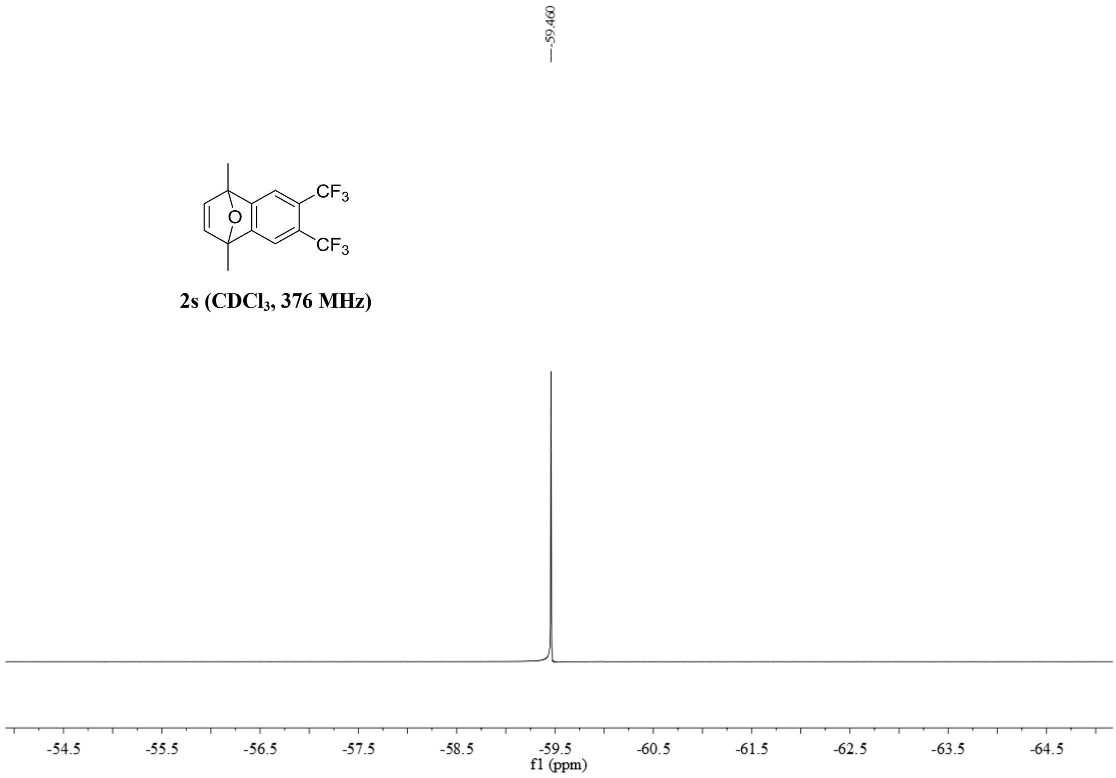

2s ($CDCl_3$, 376 MHz)

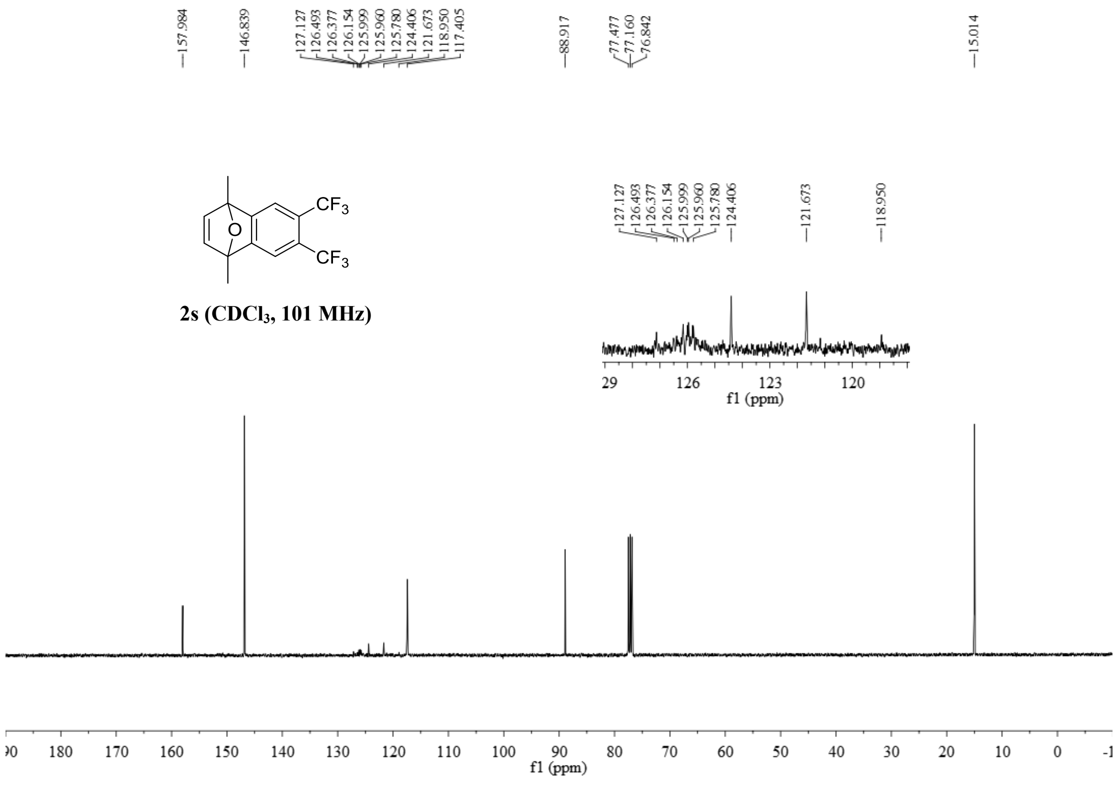

2s ($CDCl_3$, 101 MHz)

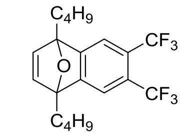

2t (CDCl$_3$, 500 MHz)

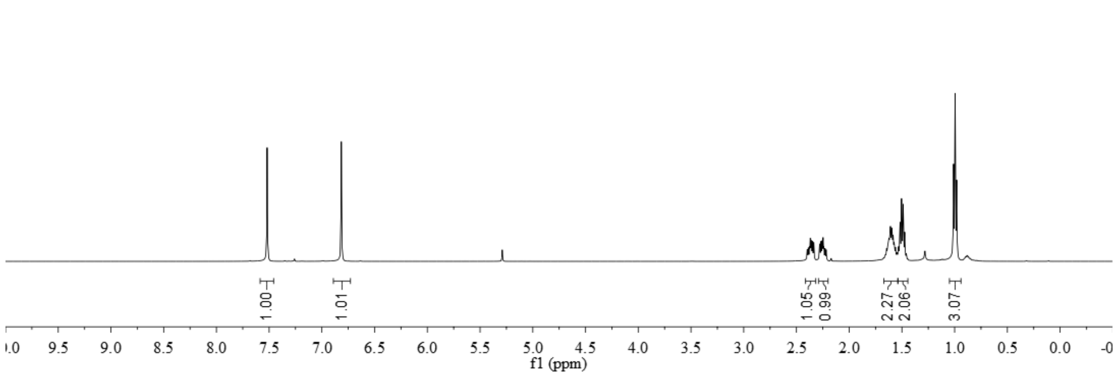

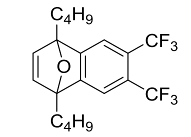

2t (CDCl$_3$, 470 MHz)

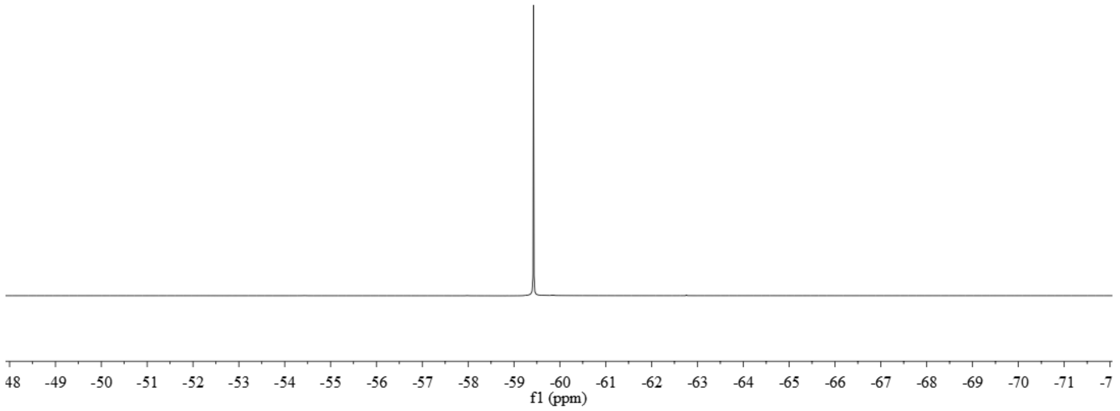

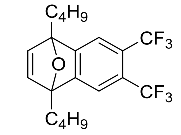

2t (CDCl$_3$, 126 MHz)

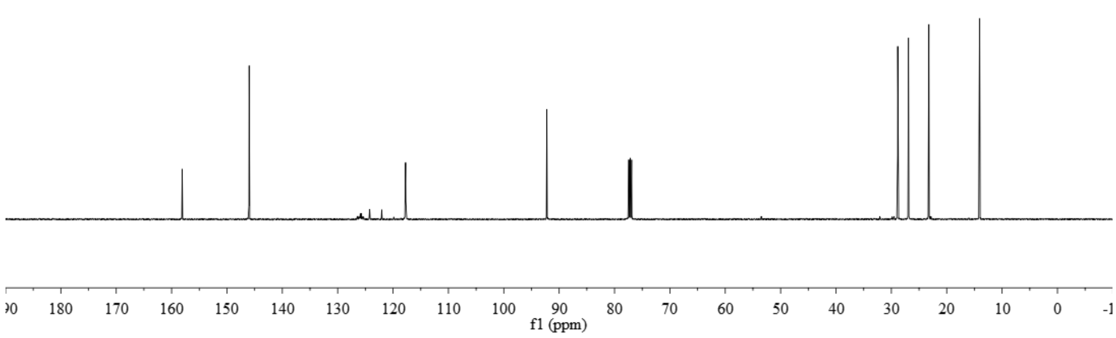

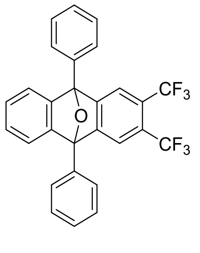

2u (CDCl$_3$, 400 MHz)

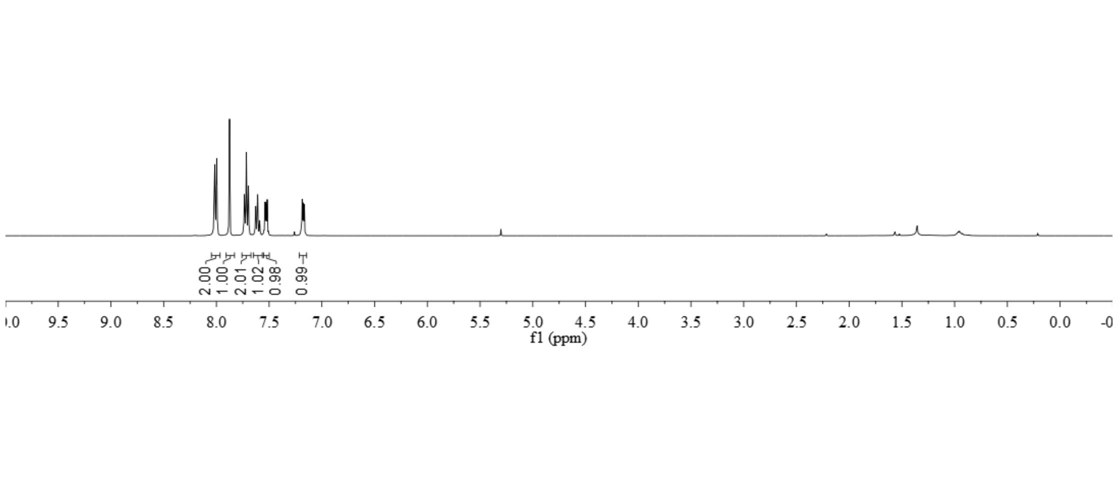

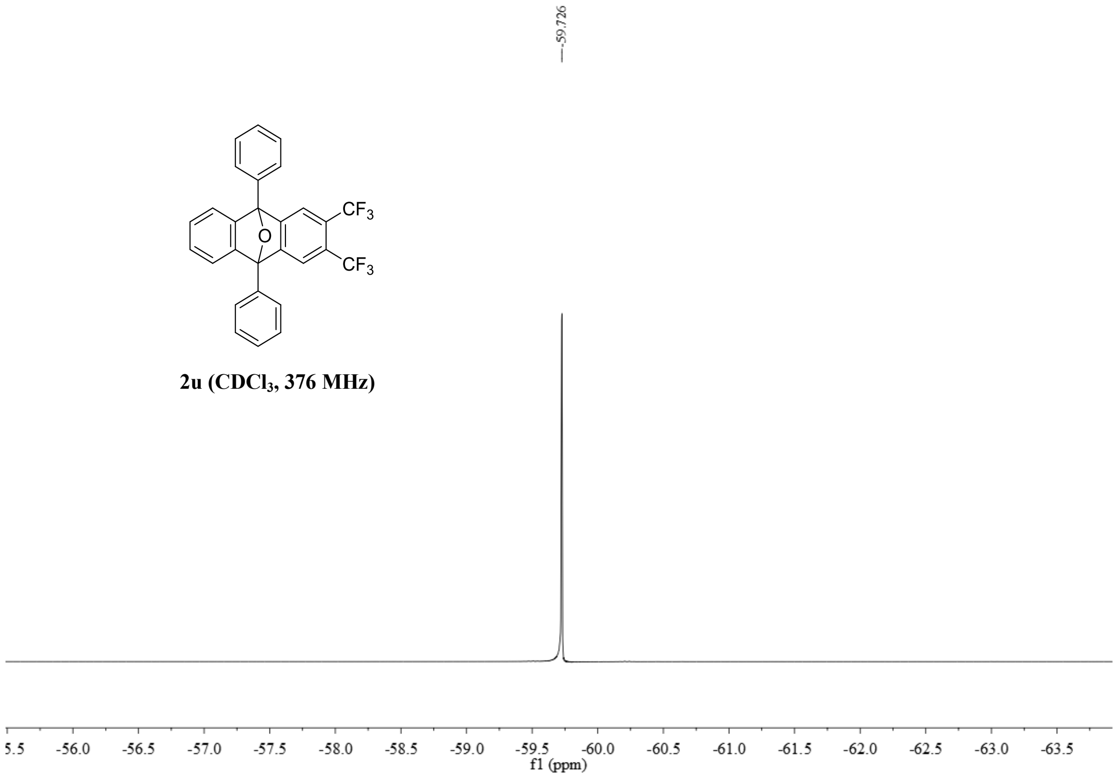

2u ($CDCl_3$, 376 MHz)

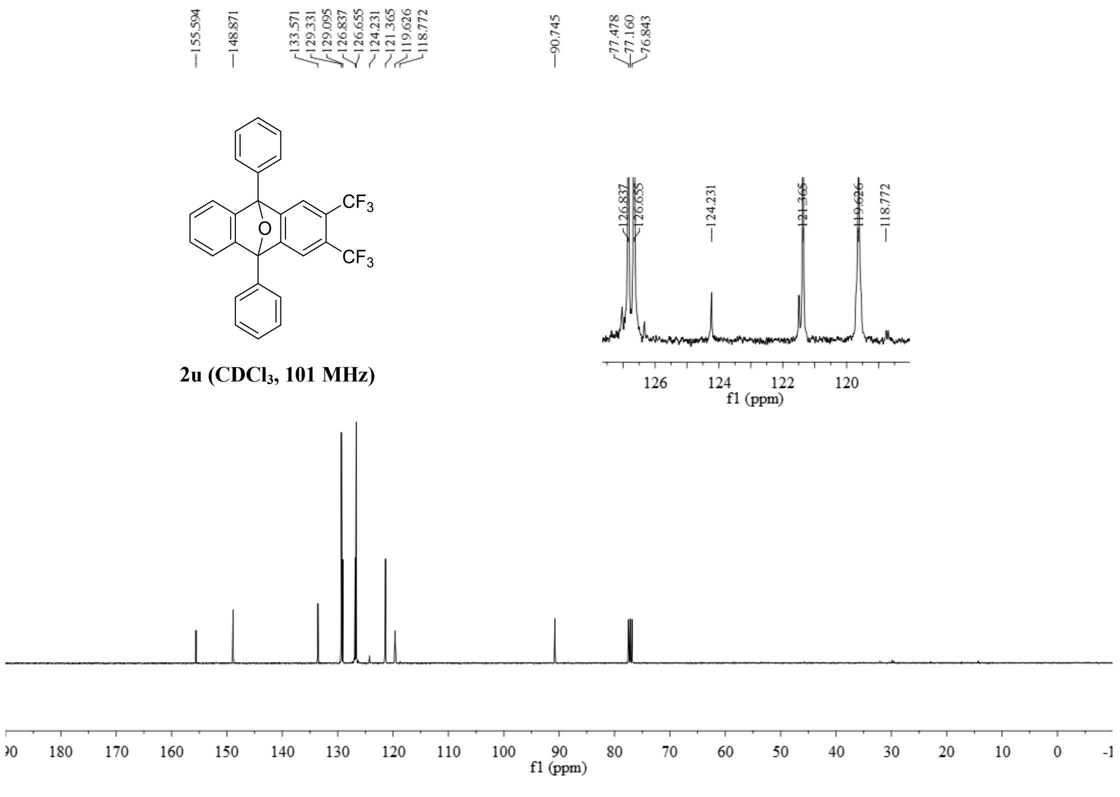

2u ($CDCl_3$, 101 MHz)

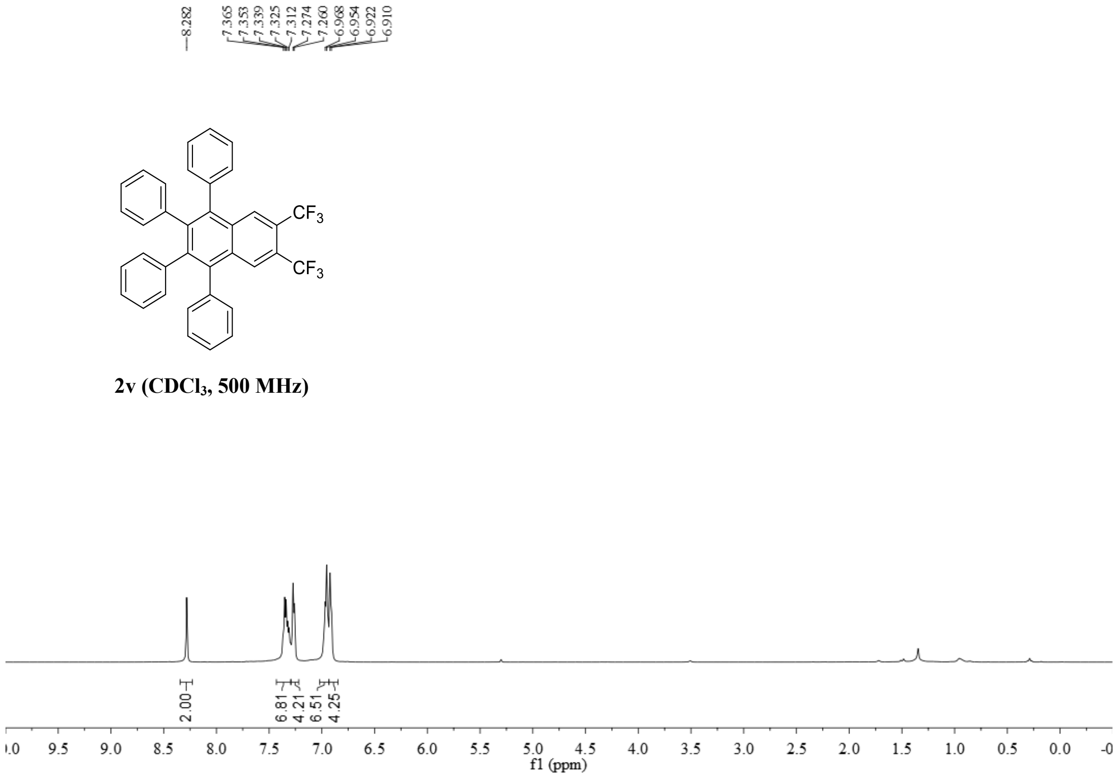

2v ($CDCl_3$, 500 MHz)

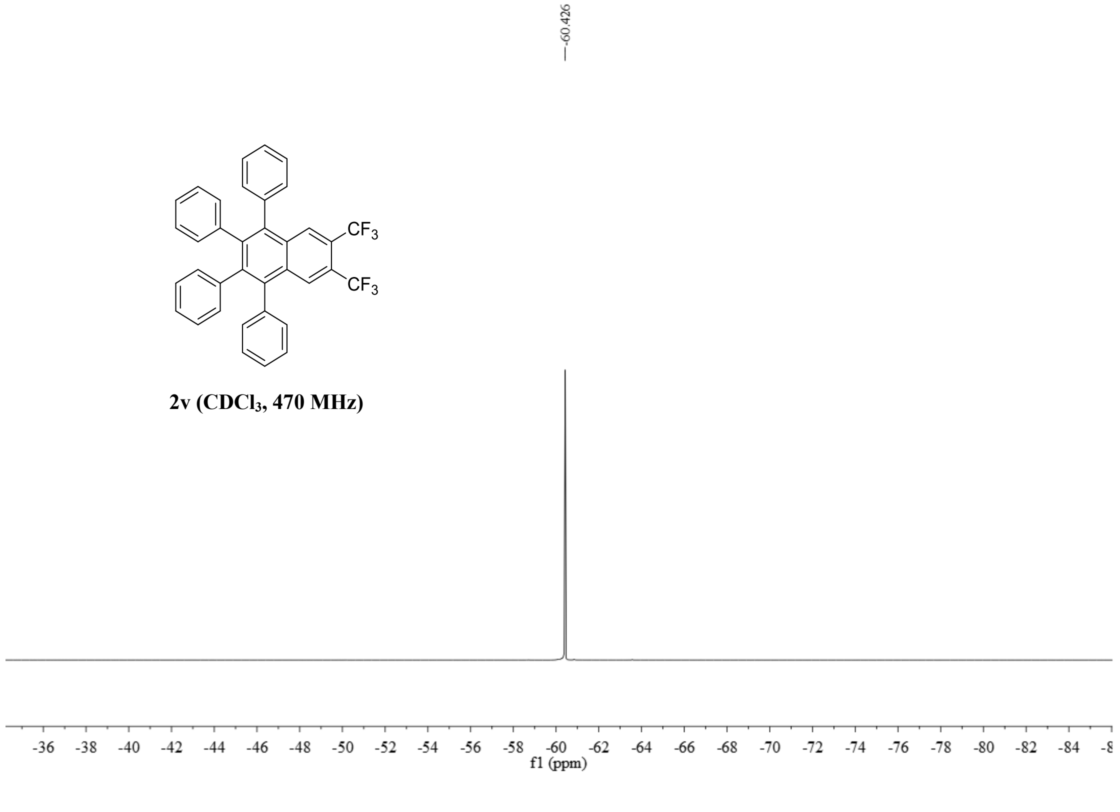

2v ($CDCl_3$, 470 MHz)

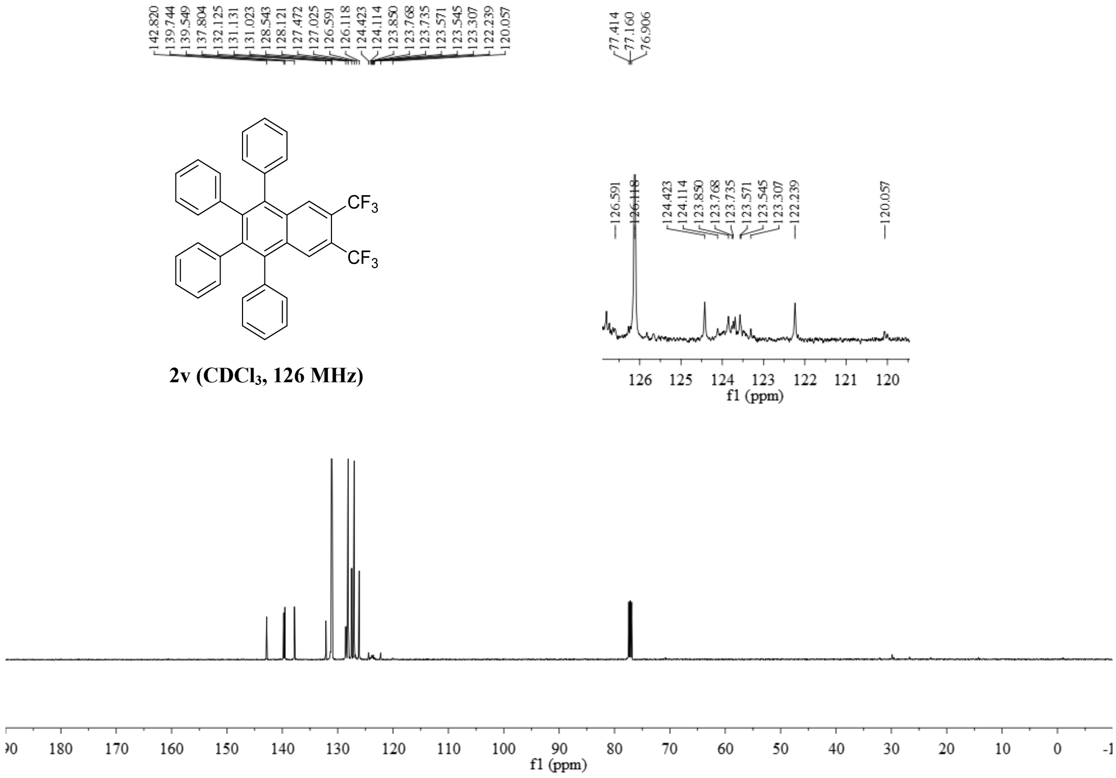

2v ($CDCl_3$, 126 MHz)

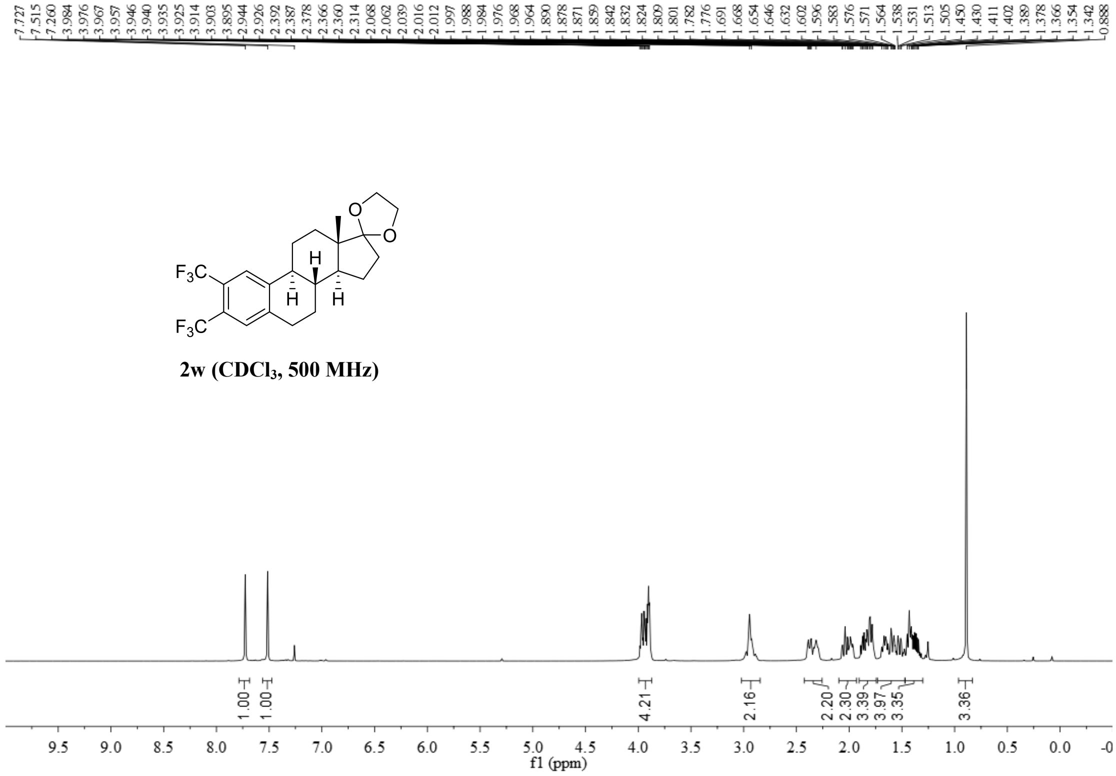

2w ($CDCl_3$, 500 MHz)

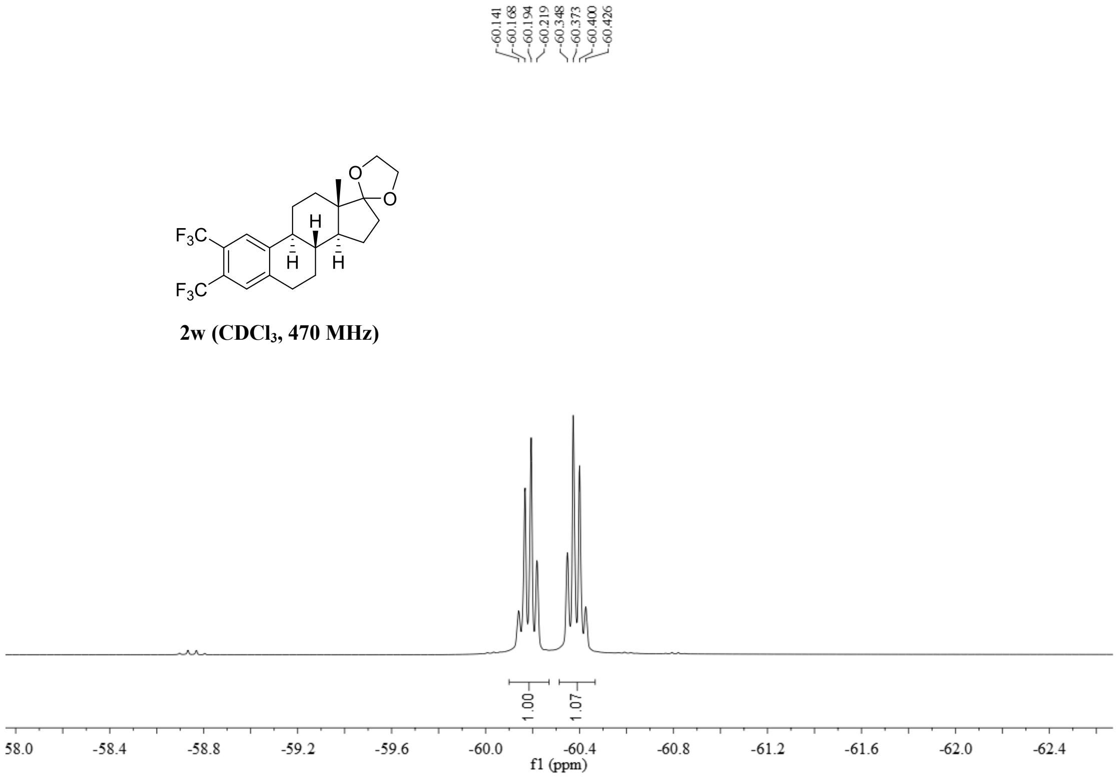

2w (CDCl$_3$, 470 MHz)

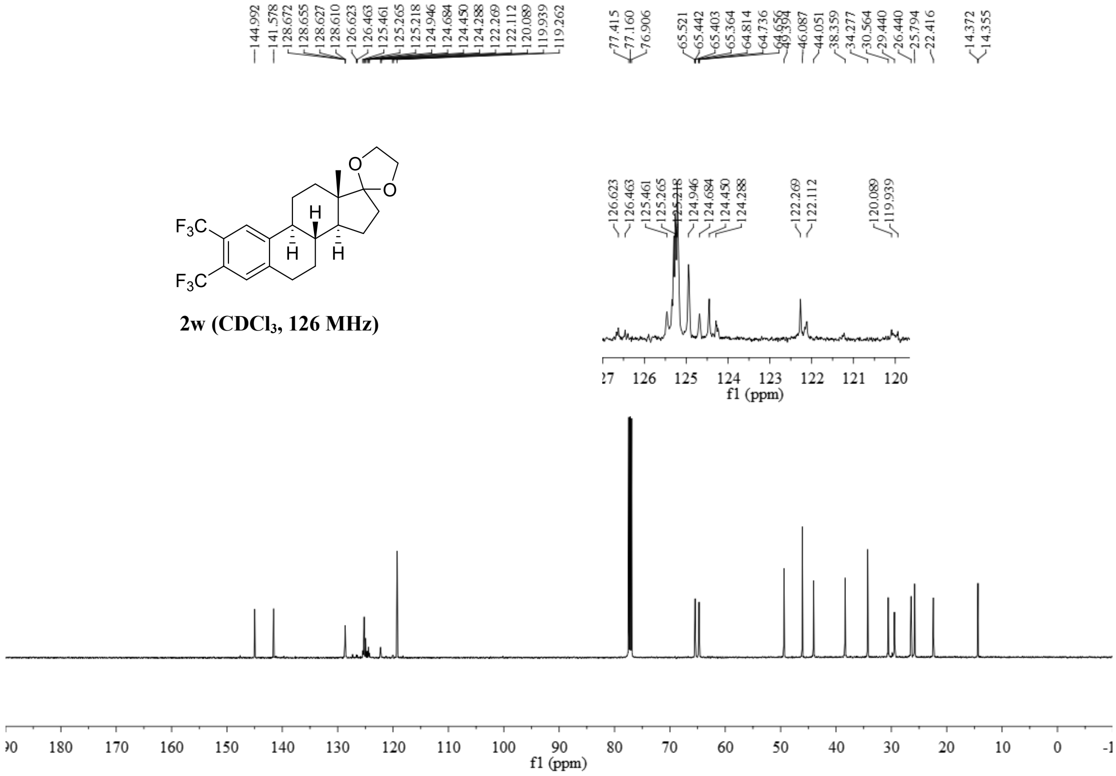

2w (CDCl$_3$, 126 MHz)

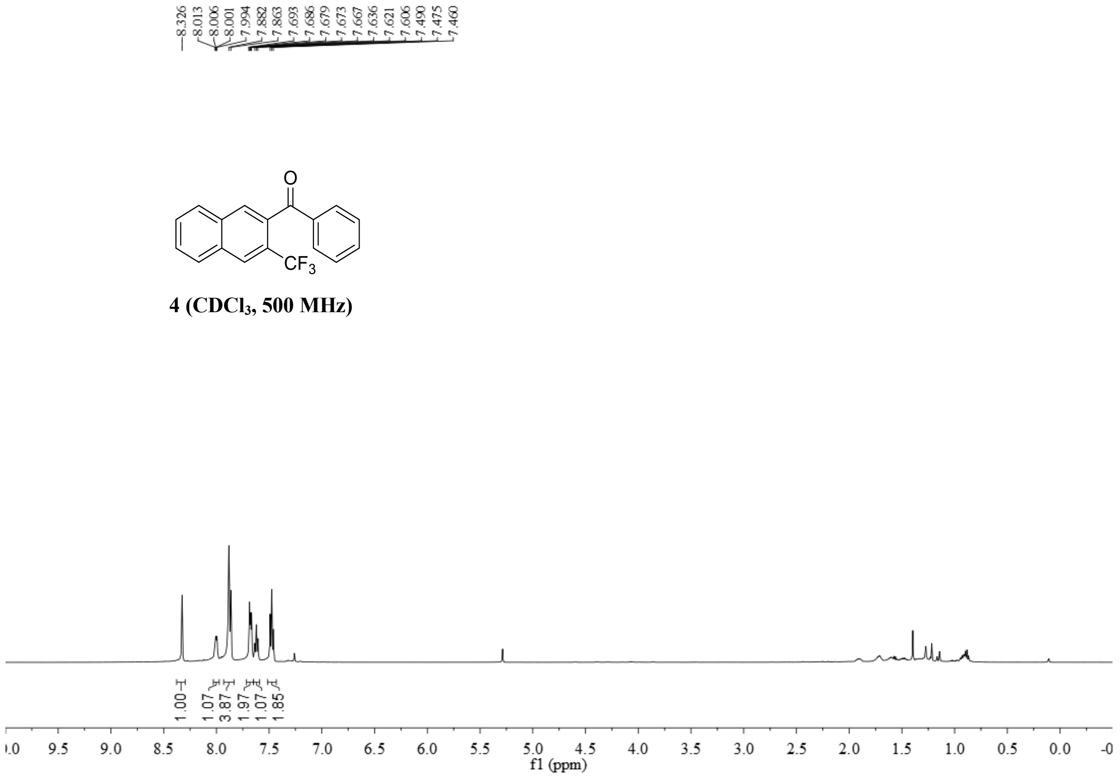

4 ($CDCl_3$, 500 MHz)

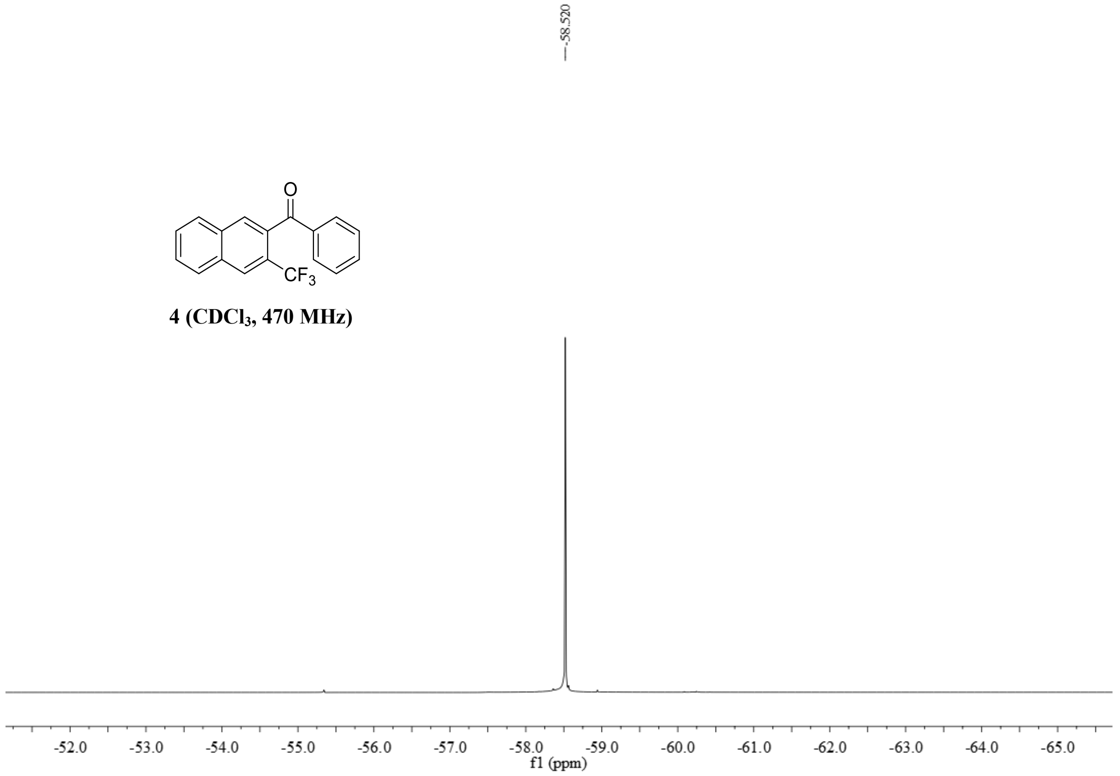

4 ($CDCl_3$, 470 MHz)

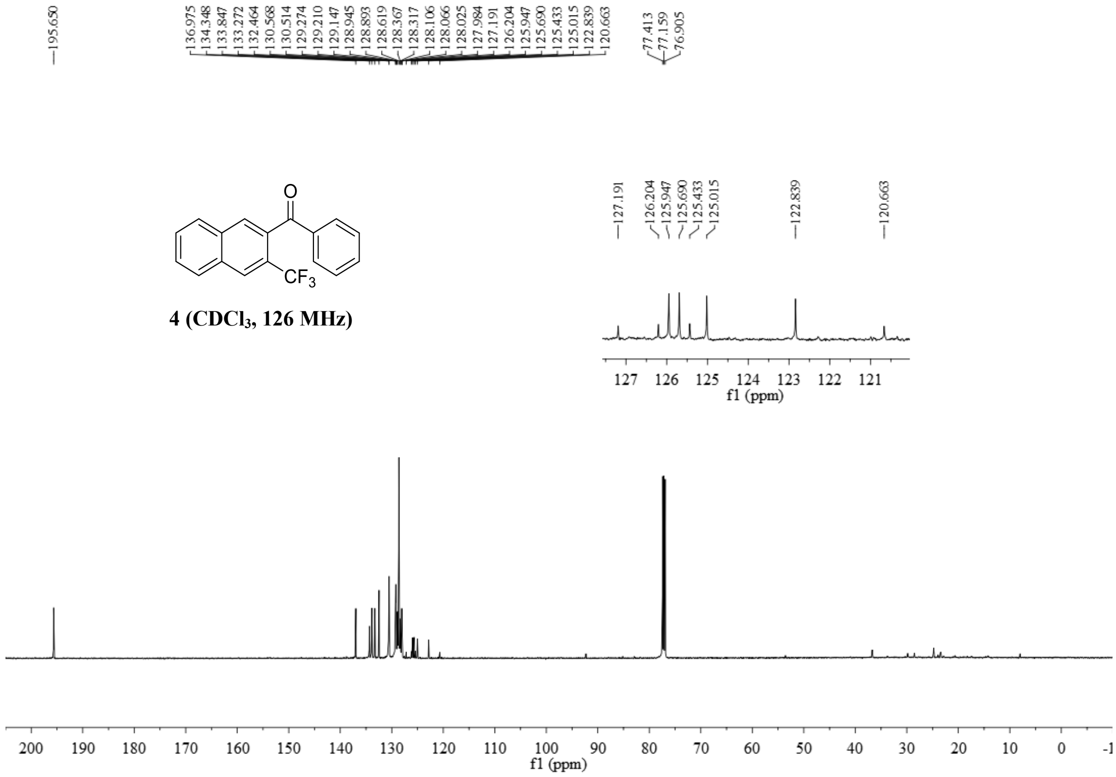

4 (CDCl$_3$, 126 MHz)

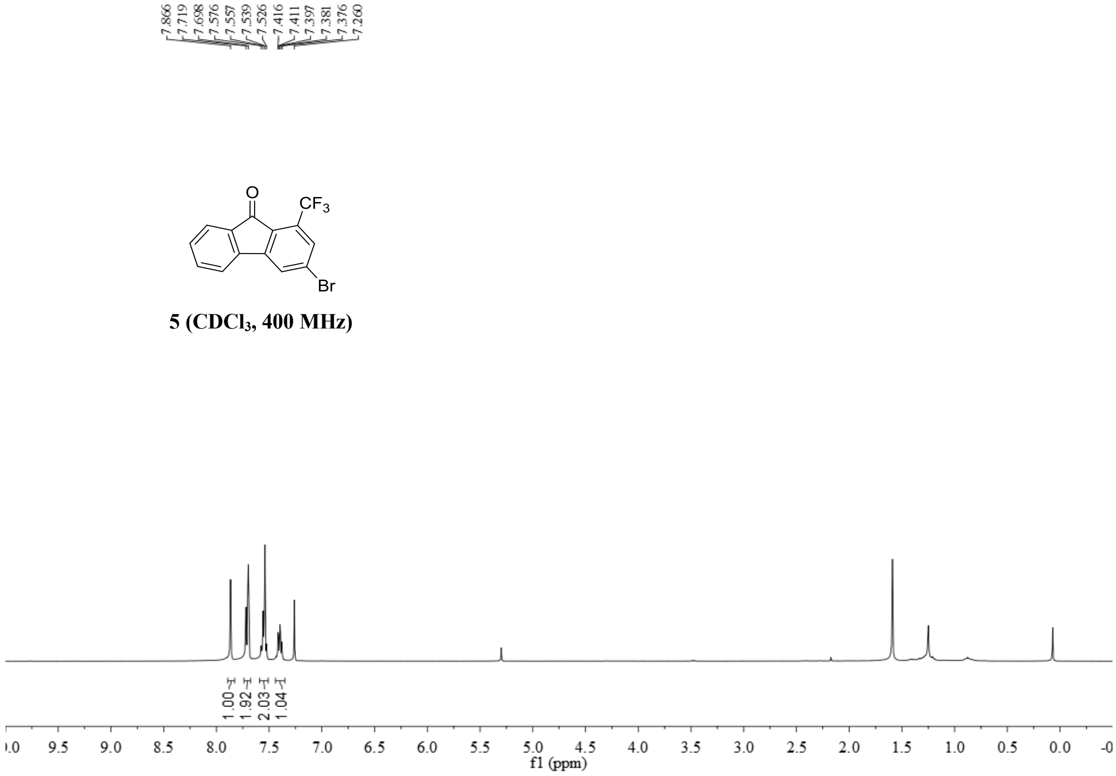

5 (CDCl$_3$, 400 MHz)

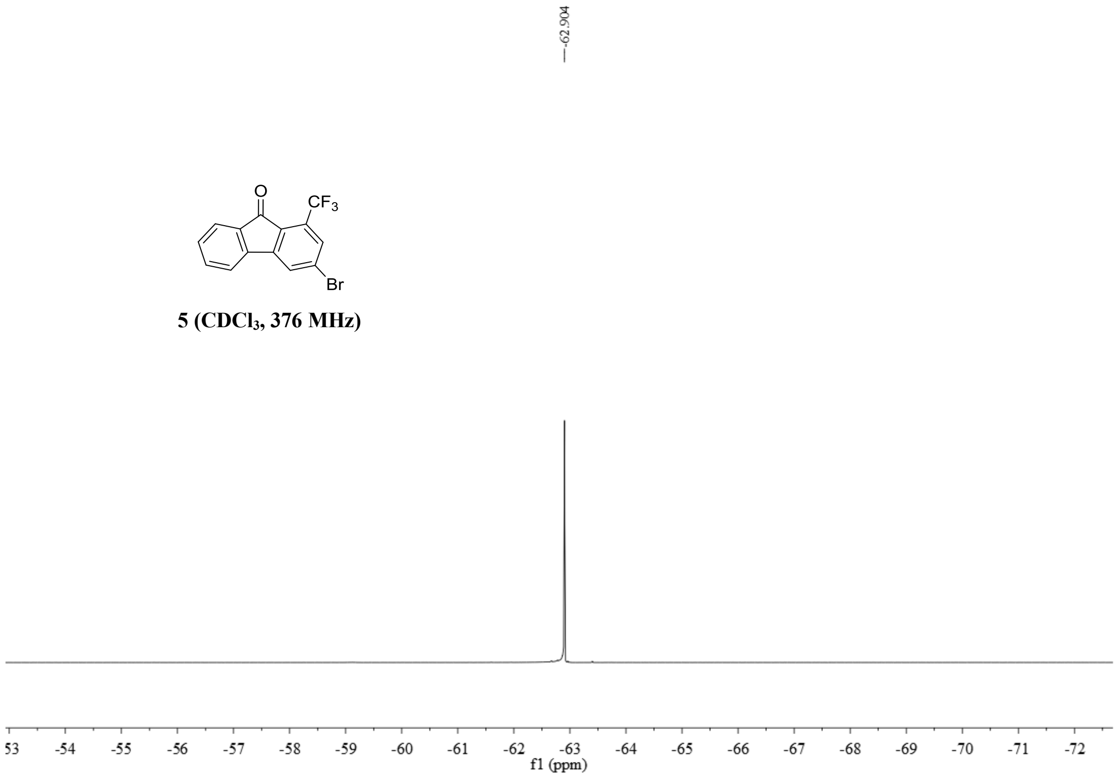

5 ($CDCl_3$, 376 MHz)

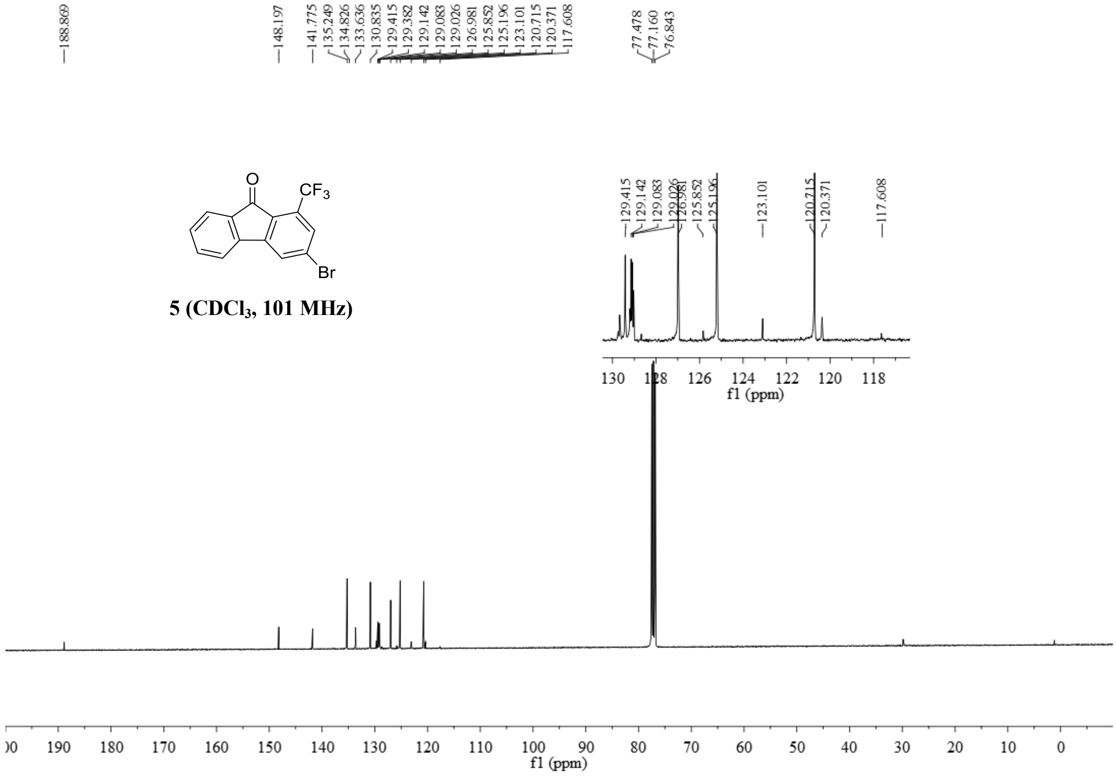

5 ($CDCl_3$, 101 MHz)

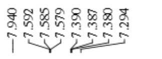

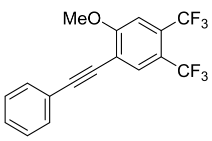

6 (CDCl$_3$, 500 MHz)

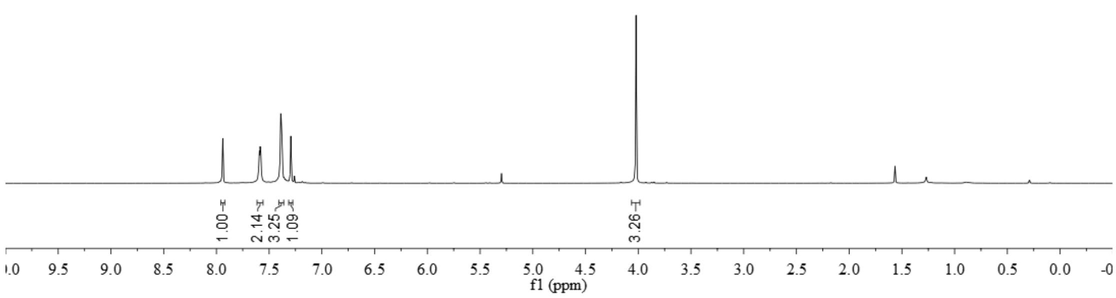

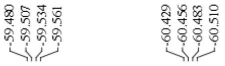

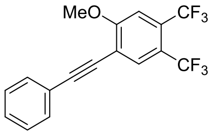

6 (CDCl$_3$, 470 MHz)

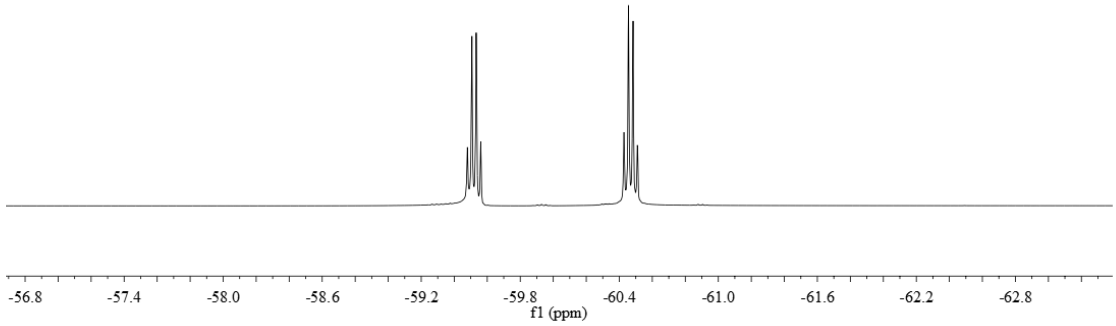

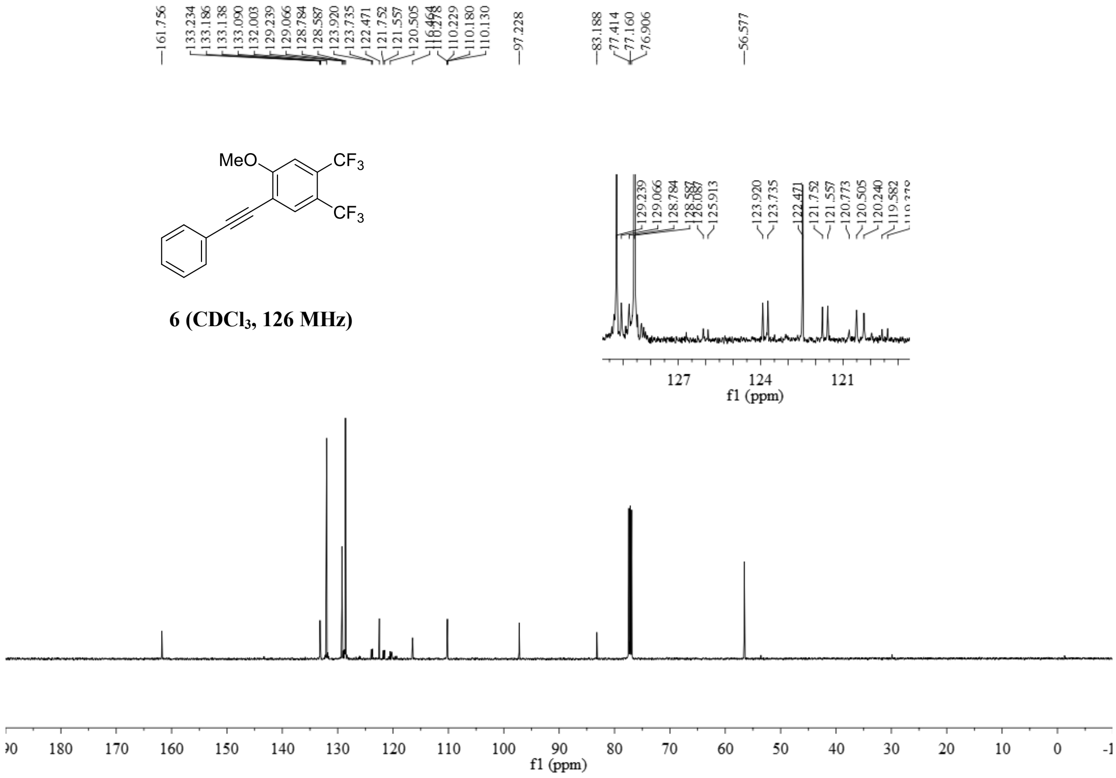

6 ($CDCl_3$, 126 MHz)

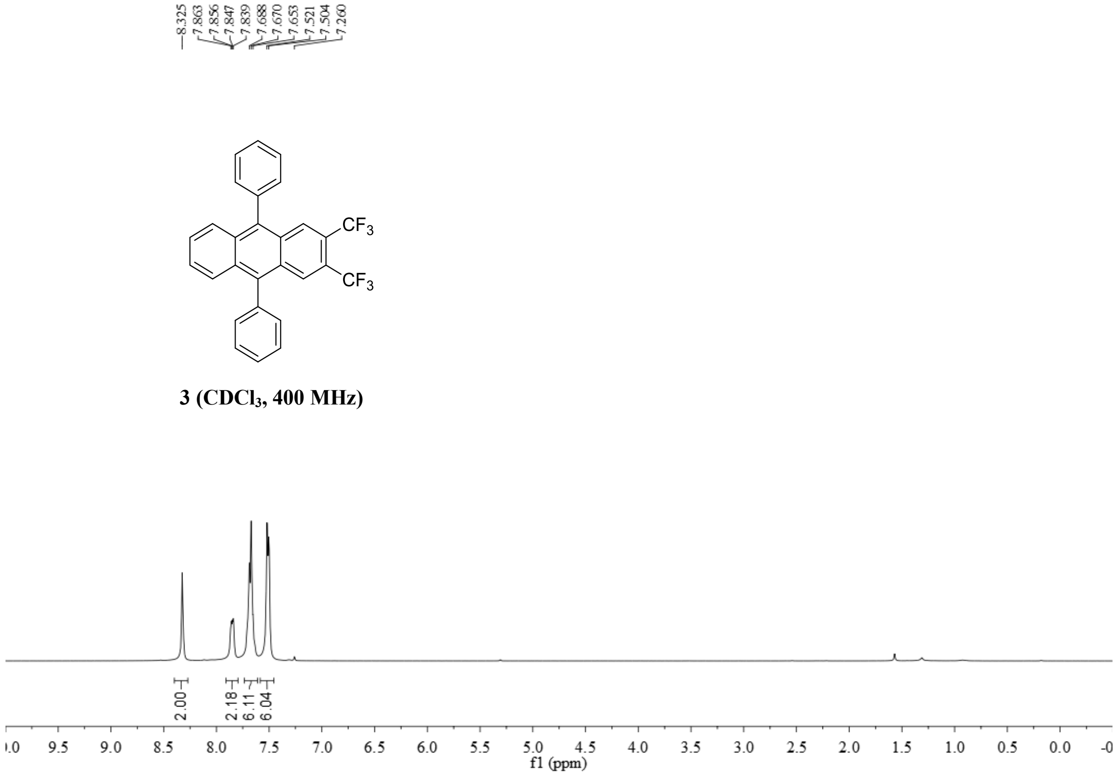

3 ($CDCl_3$, 400 MHz)

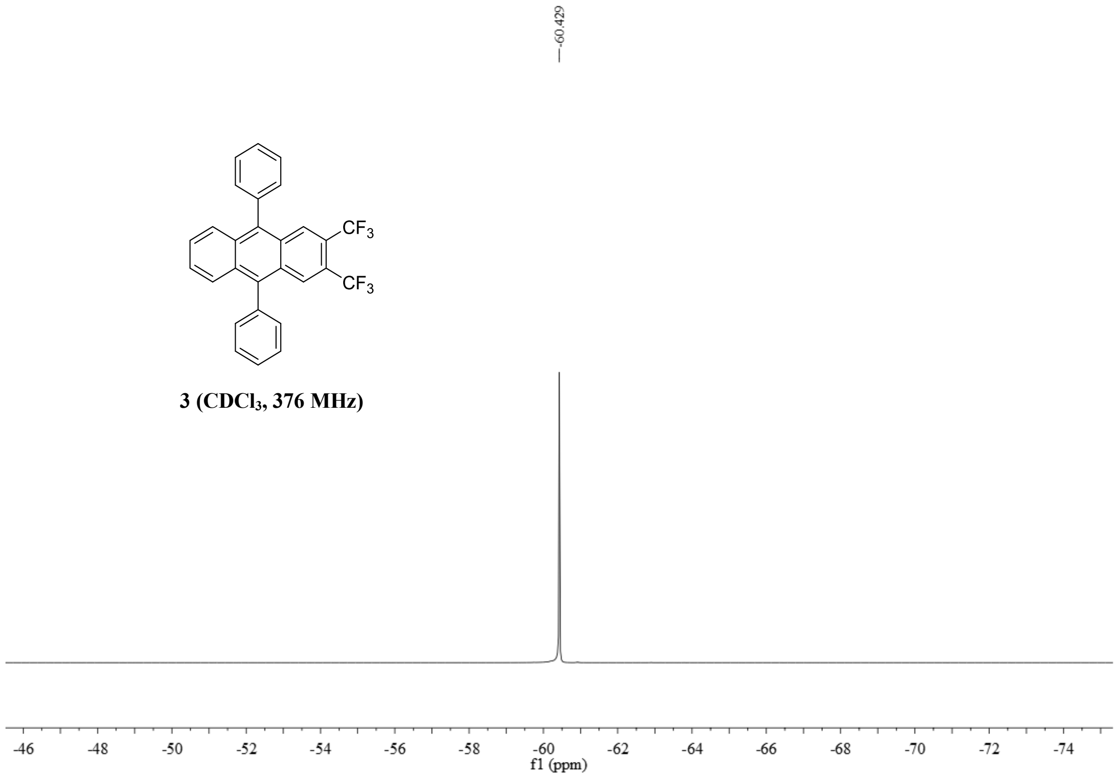
-60.429
CF3
CF3
3 (CDCl3, 376 MHz)
-46 -48 -50 -52 -54 -56 -58 -60 -62 -64 -66 -68 -70 -72 -74
f1 (ppm)

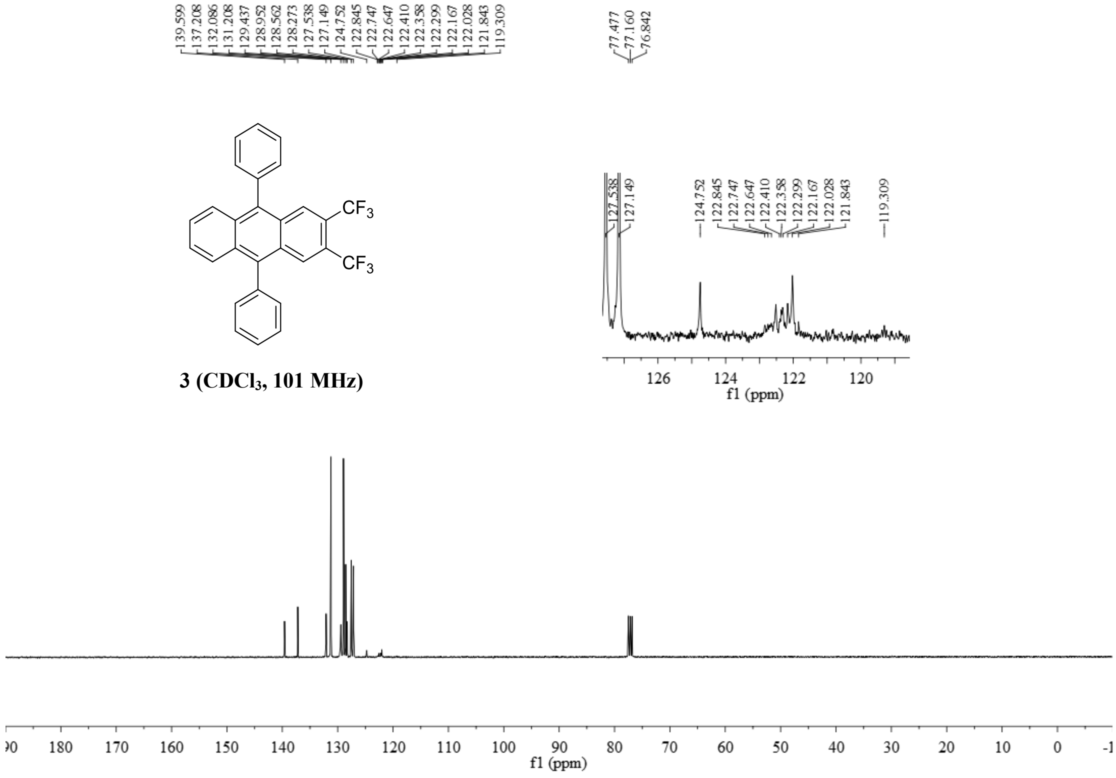
139.599
137.208
132.086
131.208
129.437
128.952
128.562
128.273
127.538
127.149
124.752
122.845
122.747
122.647
122.410
122.358
122.299
122.167
122.028
121.843
119.309
77.477
77.160
76.842
CF3
CF3
3 (CDCl3, 101 MHz)
127.538
127.149
124.752
122.845
122.747
122.647
122.410
122.358
122.299
122.167
122.028
121.843
119.309
126 124 122 120
f1 (ppm)
180 170 160 150 140 130 120 110 100 90 80 70 60 50 40 30 20 10 0
f1 (ppm)

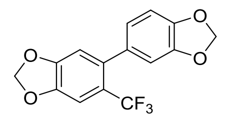

2d'' (CDCl$_3$, 500 MHz)

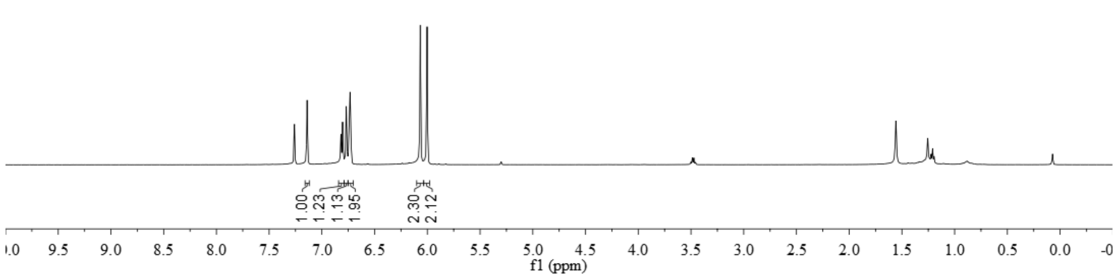

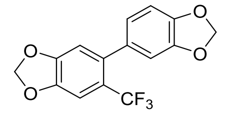

2d'' (CDCl$_3$, 470 MHz)

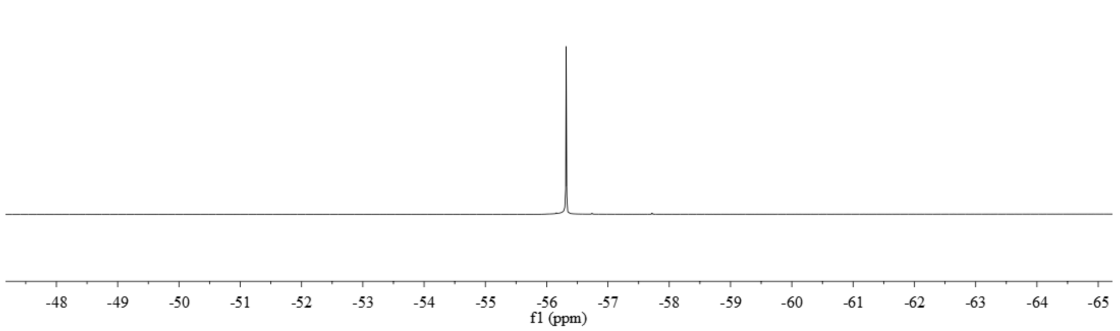

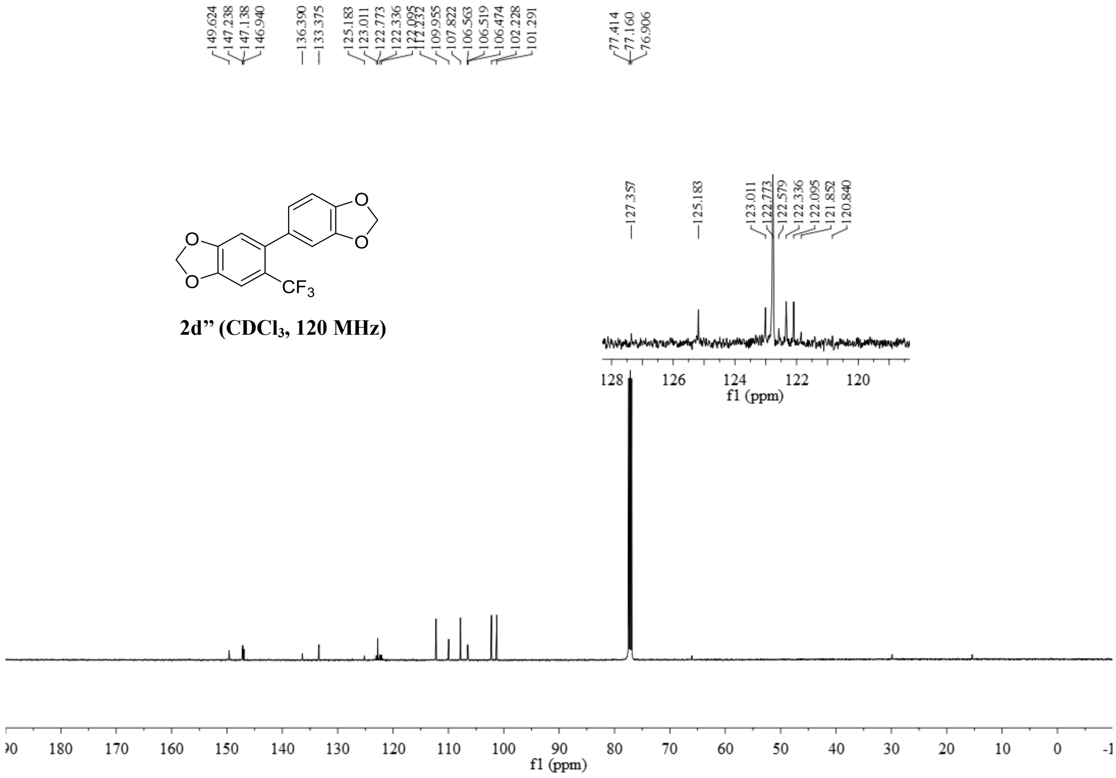

2d'' ($CDCl_3$, 120 MHz)

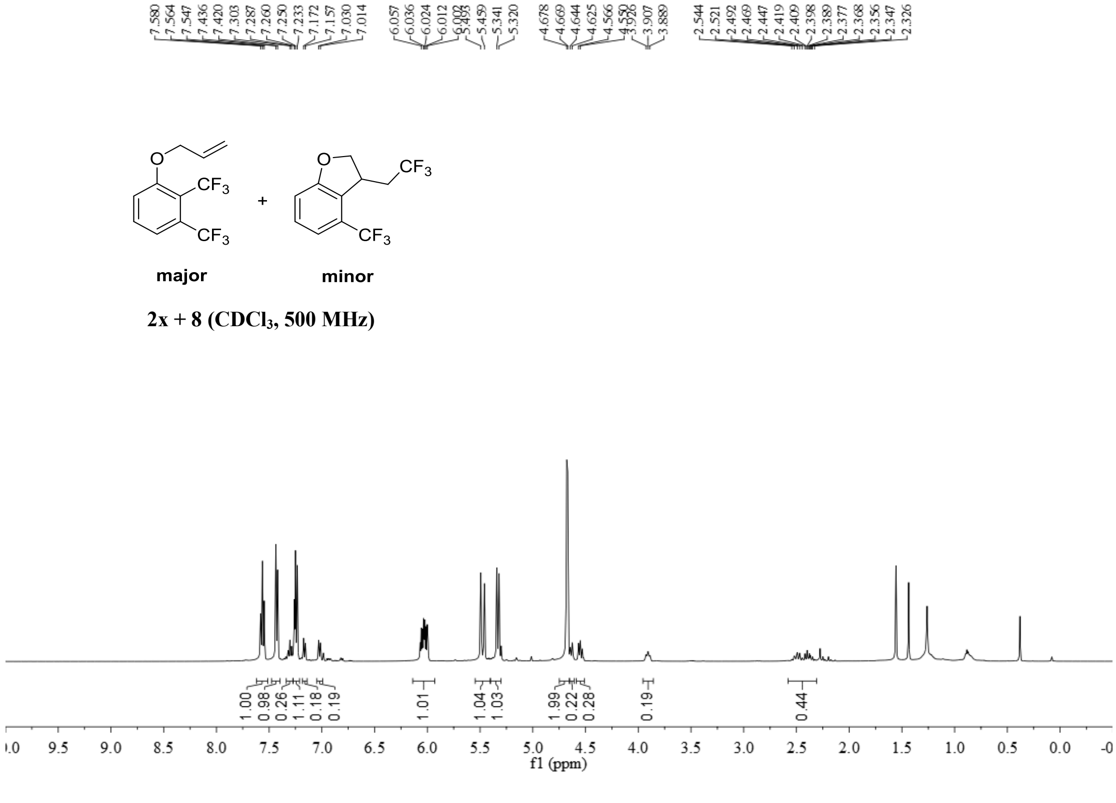

2x + 8 ($CDCl_3$, 500 MHz)

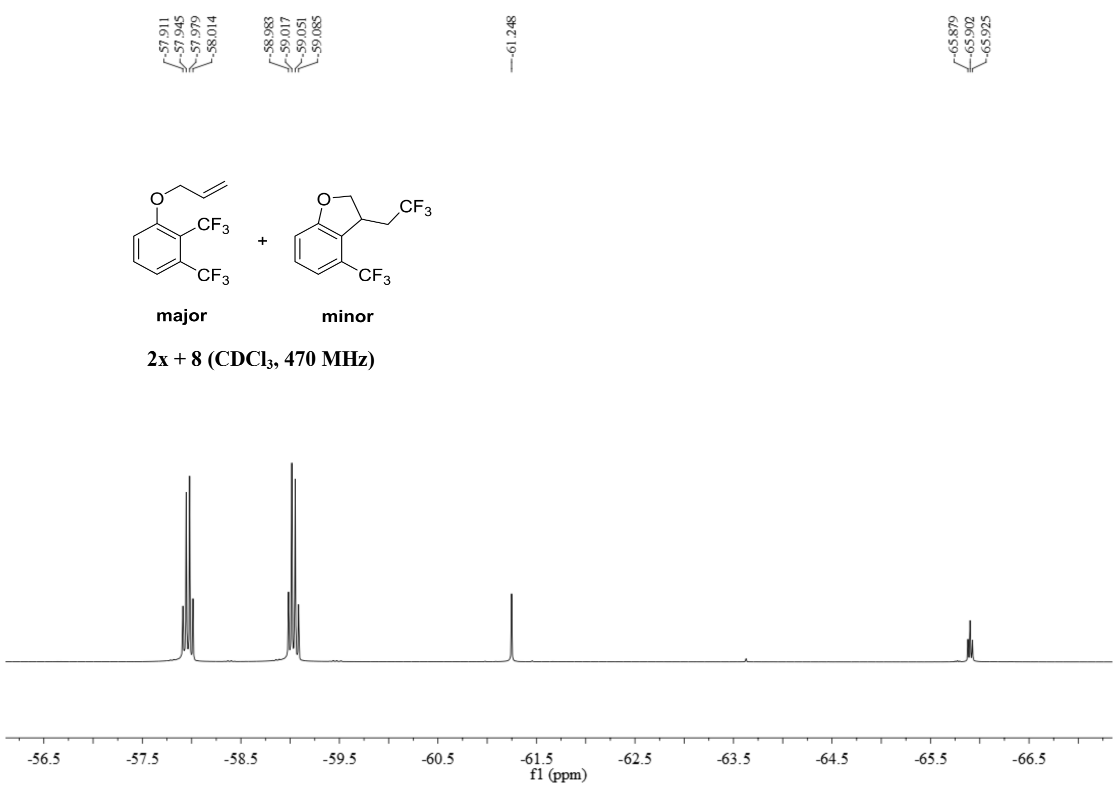

-57.911
-57.945
-57.979
-58.014
-58.983
-59.017
-59.051
-59.085
-61.248
-65.879
-65.902
-65.925
CF_3
CF_3
+
CF_3
CF_3
major
minor
2x + 8 ($CDCl_3$, 470 MHz)
f1 (ppm)

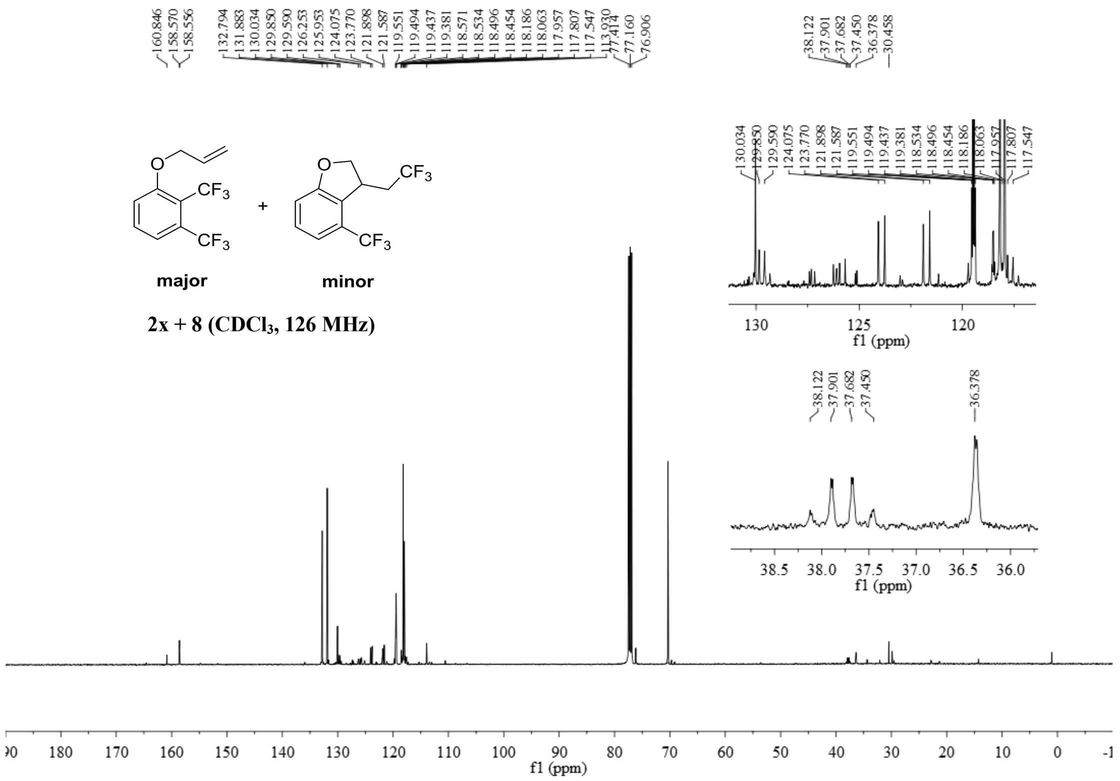

160.846
158.570
158.556
132.794
131.883
130.034
129.850
129.590
126.253
125.953
124.075
123.770
121.898
121.587
119.551
119.494
119.437
119.381
118.571
118.534
118.496
118.454
118.186
118.063
117.957
117.807
117.547
113.930
77.414
77.160
76.906
38.122
37.901
37.682
37.450
36.378
30.458
CF_3
CF_3
+
CF_3
CF_3
major
minor
2x + 8 ($CDCl_3$, 126 MHz)
f1 (ppm)
